# TGF-Δ isoforms in cancer: Immunohistochemical expression and Smad-pathway-activity-analysis in thirteen major tumor types with a critical appraisal of antibody specificity and immunohistochemistry assay validity

**DOI:** 10.18632/oncotarget.5780

**Published:** 2015-09-22

**Authors:** Markus J. Riemenschneider, Maria Hirblinger, Arabel Vollmann-Zwerenz, Peter Hau, Martin A. Proescholdt, Frank Jaschinski, Tanja Rothhammer-Hampl, Katja Wosikowski, Michel Janicot, Eugen Leo

**Affiliations:** ^1^ Department of Neuropathology, Regensburg University Hospital, Regensburg, Germany; ^2^ Wilhelm Sander Neuro-Oncology Unit, Regensburg University Hospital, Regensburg, Germany; ^3^ Department of Neurology, Regensburg University, Regensburg, Germany; ^4^ Department of Neurosurgery, Regensburg University Hospital, Regensburg, Germany; ^5^ Isarna Therapeutics GmbH, Munich, Germany

**Keywords:** TGF-Δ1/2, p-Smad2/3, cancer, glioma, carcinoma, Pathology Section

## Abstract

The literature on TGF-Δ in cancer including data on the expression or activation of TGF-Δ pathway components in specific tumors types is steadily growing. However, no systematic and uniform analysis exists reporting expression levels of the main TGF-Δ pathway components across the most frequent tumor types. We used a standardized immunohistochemical assay investigating TGF-Δ isoform expression and pathway activation across 13 different tumor types and corresponding non-neoplastic tissues. The study was performed on tissue microarrays allowing for the parallel analysis of a total of 1638 human tumor samples. TGF-Δ1, TGF-Δ2 and p-Smad2/3 were substantially expressed in multiple cancers widening the options for TGF-Δ isoform directed therapies. Of note, TGF-Δ antigens appear to be expressed in an individual manner pointing towards a need for patient preselection for TGF-β isoform specific treatment. Yet, a thorough investigation of antibody specificity and assay validity revealed that immunohistochemistry did not correlate with other detection methods on mRNA or protein level in all instances. As such, with the currently available means (i.e. antibodies tested) a stratification of patients within clinical trials for TGF-Δ directed antisense therapies based upon TGF-β immunohistochemistry alone has to be interpreted with caution and should be carefully evaluated in combination with other parameters.

## INTRODUCTION

Transforming growth factor-Δ comprises three isoforms (TGF-Δ1, TGF-Δ2 and TGF-Δ3) and has been identified as a potent regulator of cell growth, differentiation, and migration in nearly all cell types and tissues.[1] Although the downstream effects of TGF-Δ signaling are largely context dependent, its signaling is at least partially conserved in many cell types.[2] Two of the main downstream mediators of TGF-Δ are the ‘Mothers against decapentaplegic homolog transcription factor’ 2 (Smad2) and Smad3 that -when phosphorylated- hetero-oligomerize with Smad4 and translocate to the nucleus where they mediate gene expression or repression.[2, 3] TGF-Δ signaling has been identified to play a major role in cancer. [4-7] Particularly in advanced tumors, the TGF-Δ signaling pathway appears to be severely dysregulated. In malignant gliomas, for example, TGF-Δ2 is associated with advanced disease stage and poor prognosis.[8]

Consequently, a targeted strategy to modulate TGF-β2 signaling was developed.[9] The antisense oligonucleotide trabedersen (AP 12009) that specifically blocks TGF-β2 mRNA was tested in several clinical trials in high-grade glioma patients with recurrent or refractory tumor disease.[10, 11] Intratumoral administration of the drug resulted in responses and promising survival data.[9] A subsequent phase III study in anaplastic astrocytoma was terminated due to low patient recruitment, partly due to factors unrelated to the treatment but also probably reflective of the challenging way of administration of the drug (intratumoral infusion over months). Encouraging signs of clinical efficacy with favorable survival outcomes for TGF-Δ2 antisense treated patients were observed for pancreatic cancer and malignant melanoma treated intravenously.[12, 13]

To potentially guide the design of further trials with an underlying ratio on TGF-Δ isoform expression/pathway activation we investigated a broad spectrum of different human neoplasms in an unbiased manner. While scattered data on the expression or activation of single TGF-Δ pathway components in specific tumors types are included in many publications,[4, 14] no analysis is available investigating systematically and uniformly the expression of the main TGF-Δ pathway components across main tumor types. By using the same standardized immunohistochemical assays on tumor tissue arrays (leading to a high and statistically evaluable number of patients) we aimed to compare the degree of TGF-Δ expression/pathway activation across the different entities and to derive information on which tumor entities might be best suited for TGF-Δ directed therapies. We also aimed to assess the degree of inter-individual TGF-Δ expression differences within the tumor types. We argued that these insights might aid to decide on whether histological tumor diagnosis alone is sufficient to indicate therapy response potential or whether an additional upfront molecular test might be necessary to more specifically identify candidate patients for TGF-Δ isoform specific therapeutic intervention. Finally, we challenged the role of immunohistochemistry in this context and compared it to alternative testing procedures on protein and mRNA level.

## RESULTS

### Comparative analysis of TGF-Δ isoform expression and pathway activation in thirteen different tumor types

A total of 16 tumor tissue arrays were analyzed for TGF-Δ1 and -2 expression as well as for Smad2/3 phosphorylation, reflective of pathway activation. The raw data for the semiquantitative assessment of the staining of all arrays are provided in Suppl. Table 1. The subsequent evaluation of immunohistochemical staining results on an array-by-array basis is documented in detail in Suppl. Figure 2. Suppl. Figure 2 also provides accurate information on the number of patients and the histological tumor types contained on the individual arrays. Taken together, tissue samples from 1638 patients were analyzed in this study. The 16 arrays corresponded to 13 different tumor types. They comprised tumors of the male and female genital tract (breast, ovary and prostate cancer), tumors of the respiratory system (lung cancer and mesothelioma), gastrointestinal tumors (pancreatic, liver and colon cancer), squamous cell carcinomas (head and neck cancer), neuroectodermal tumors (malignant melanoma, brain tumors) and tumors of the lymphatic system (lymphoma and myeloma). For pancreatic cancer, brain cancer and lymphoma two arrays were evaluated, respectively.

To compare TGF-Δ expression across the different arrays/tumor types we computed mean protein expression levels and percentages of positive tumors for the individual tumor types (Table 1). As for the percentages of immunoreactive tumors, we first calculated the percentage of patients that showed any, i.e. also low (staining score >0, % positive tumors), immunoreactivity. However, in most entities this score provided antigen immunoreactivity frequencies of about 90% and thus did not appear to reach optimal selectivity in terms of assessing expression differences in TGF-Δ pathway proteins. We then calculated percentages of tumors that reached an expression score of >1 (% relevantly positive tumors) assuming that this score could add in more sharply delineating differences between the individual tumor entities.

**Table 1:** Comparative analysis of 16 tumor arrays (13 different tumor tissue types) in respect to TGF-Δ isoform expression and pathway activation

	% positive tumors (score >0)	TGF-Δ1	TGF-Δ2 (Acris)	TGF-Δ2 (SC)	p-Smad2	summarized score
1	BR10010: **Breast cancer** and matched metastatic carcinoma tissue array	99	92	90	95	376
2	MS801: **Mesothelioma** tissue array with normal mesothelium	97	84	95	93	369
3	OV2086: **Ovary cancer** survey tissue array	98	90	90	87	365
4	PA1921: Mid-advanced stage **pancreatic cancer** tissue array	89	86	91	95	361
5	LC20813: **Lung cancer** tissue array	97	85	83	86	351
6	LY2086: **Lymphoma** tumor tissue array	97	72	89	91	349
7	HN483: Multiple **head and neck cancer** with normal tissue array	92	62	88	100	342
8	CO1503: **Colon Cancer** tissue array	89	71	93	88	341
9	PR8010: **Prostate cancer** tissue array	83	77	83	95	338
10	GL803a: **Brain tumor** and adjacent tissue array	96	58	92	91	337
11	BC03119: **Liver carcinoma** and normal tissue	95	61	85	91	332
12	LM803: **Lymphoma** and normal lymph node tissue array	92	77	68	93	330
13	BM483: Tumor tissue array **(Myeloma)**	69	62	92	100	323
14	PA2081a: **Pancreatic** disease spectrum tissue array	77	77	77	84	315
15	GL2083a: **Brain tumor** tissue array	72	38	77	91	278
16	ME2082b: **Malignant Melanoma** tissue array	47	34	84	77	242

	% relevantly positive tumors (score >0)	TGF-Δ1	TGF-Δ2 (Acris)	TGF-Δ2 (SC)	p-Smad2	summarized score
1	BR10010: **Breast cancer** and matched metastatic carcinoma tissue array	52	50	47	65	214
2	MS801: **Mesothelioma** tissue array with normal mesothelium	40	38	57	67	202
3	BM483: Tumor tissue array **(Myeloma)**	23	15	83	73	194
4	BC03119: **Liver carcinoma** and normal tissue	49	9	51	62	171
5	LC20813: **Lung cancer** tissue array	36	32	44	57	169
6	OV2086: **Ovary cancer** survey tissue array	24	33	40	60	157
7	LY2086: **Lymphoma** tumor tissue array	42	2	41	55	140
8	PA1921: Mid-advanced stage **pancreatic cancer** tissue array	24	32	31	52	139
9	HN483: Multiple **head and neck cancer** with normal tissue array	16	14	50	54	134
10	GL803a: **Brain tumor** and adjacent tissue array	43	7	30	50	130
11	ME2082b: **Malignant Melanoma** tissue array	22	10	44	49	125
12	CO1503: **Colon Cancer** tissue array	32	13	28	46	119
13	PA2081a: **Pancreatic** disease spectrum tissue array	17	18	21	48	104
14	PR8010: **Prostate cancer** tissue array	21	6	23	53	103
15	LM803: **Lymphoma** and normal lymph node tissue array	16	12	25	37	90
16	GL2083a: **Brain tumor** tissue array	6	3	31	46	86

	mean protein expression levels	TGF-Δ1	TGF-Δ2 (Acris)	TGF-Δ2 (SC)	p-Smad2	summarized score
1	MS801: **Mesothelioma** tissue array with normal mesothelium	1.5	1.4	1.7	1.9	6.4
2	BR10010: **Breast cancer** and matched metastatic carcinoma tissue array	1.7	1.4	1.3	1.6	6.0
3	BM483: Tumor tissue array **(Myeloma)**	1.0	0.8	2.0	2.0	5.9
4	OV2086: **Ovary cancer** survey tissue array	1.3	1.3	1.5	1.6	5.6
5	LC20813: **Lung cancer** tissue array	1.4	1.3	1.3	1.5	5.6
6	BC03119: **Liver carcinoma** and normal tissue	1.6	0.7	1.6	1.7	5.5
7	LY2086: **Lymphoma** tumor tissue array	1.6	0.7	1.4	1.7	5.3
8	PA1921: Mid-advanced stage **pancreatic cancer** tissue array	1.2	1.2	1.3	1.5	5.2
9	HN483: Multiple **head and neck cancer** with normal tissue array	1.1	0.8	1.4	1.6	4.9
10	CO1503: **Colon Cancer** tissue array	1.3	0.8	1.3	1.4	4.8
11	PA2081a: **Pancreatic** disease spectrum tissue array	1.0	1.0	1.1	1.6	4.7
12	GL803a: **Brain tumor** and adjacent tissue array	1.5	0.6	1.3	1.3	4.7
13	PR8010: **Prostate cancer** tissue array	1.1	0.8	1.1	1.6	4.7
14	LM803: **Lymphoma** and normal lymph node tissue array	1.1	0.9	1.1	1.3	4.4
15	ME2082b: **Malignant Melanoma** tissue array	0.7	0.5	1.4	1.5	4.0
16	GL2083a: **Brain tumor** tissue array	0.8	0.4	1.2	1.4	3.8

Color code for the single antigens and the summarized protein expression score: Median value of an individual tissue type around the median of all tissue types = white; expression more frequent/elevated in comparison to the median = shades of green, the higher, the darker; expression less frequent/decreased in comparison to the median = shades of red, the higher, the darker

To visualize differences between the arrays/tumor types, for each specific antigen the median value of an array (either expression level or percentage of immunoreactive tumors) was compared to the median value of this antigen across all tumor arrays. The median value for the individual array was then color-coded according to its deviation from the median value across all arrays and Table 1 formatted accordingly. To finally rank the arrays according to either % positive tumors (score >0), % relevantly positive tumors (score >1) or mean protein expression levels, we then calculated a combined score by summarizing the scores of all four (TGF-Δ1, TGF-Δ2(Acris), TGF-Δ2(SC) and p-Smad2/3) immunohistochemical assays for each single tissue type. We assumed that this score might give a reasonable overall impression of the extent of TGF-Δ signaling pathway-related protein expression in the individual tumor types (Table 1).

The different analyses produced widely overlapping results. Highest (or most widespread) TGF-Δ pathway protein immunoreactivity was observed in breast cancer and mesothelioma. These two tumor types (in all comparisons) ranked in position 1 or 2. Other tumor entities with high TGF-Δ pathway protein expression were ovary cancer, myeloma, lung cancer, lymphoma, pancreatic and head and neck cancer. Tumor entities that demonstrated comparably low TGF-Δ pathway protein expression were melanomas, brain cancers and prostate cancers (Table 1).

### TGF-Δ isoform expression and pathway activation in non-neoplastic tissues

To compare the expression of TGF-Δ pathway proteins in non-neoplastic tissues in a comparable fashion, we evaluated the non-neoplastic tissues that were contained on some of the tumor arrays. To generate data from non-neoplastic tissues corresponding to all cancer types, we additionally stained the FDA normal organ tissue array (FDA808ci-1). This array contained 75 cores from 75 patients and comprised a variety of non-neoplastic tissues spotted in triplicates in their majority. The raw data for the semiquantitative assessment of staining of the non-neoplastic tissues are provided in Suppl. Table 1.

Table 2 visualizes the extent of expression of the different antigens in the different non-neoplastic tissue types. As described before, for each antigen mean protein expression levels of an individual non-neoplastic tissue type were compared to the median expression of this specific antigen over all non-neoplastic tissue types. Arrays were then ranked again according to a summarized score including the scores of all four immunohistochemical assays for each single tissue type (Table 2).

**Table 2:** Comparative analysis of TGF-Δ isoform expression and pathway activation in different non-neoplastic tissues

	mean protein expression levels in non-neoplastic tissues	TGF-Δ1	TGF-Δ2 (Acris)	TGF-Δ2 (SC)	p-Smad2	summarized score
1	HN483: Non-neoplastic tissue from HN483 **(Head and neck cancer)**	0.3	0.6	1.5	1.1	3.5
2	GL803a: **Brain tissue (Cerebrum)** from FDA array	1.0	0.7	1.0	1.0	3.7
3	GL2083a: **Brain tissue (Cerebrum)** from FDA array	1.0	0.7	1.0	1.0	3.7
4	PR8010: **Prostate tissue** from FDA array	1.0	1.3	1.0	0.7	4.0
5	ME2082b: Non-neoplastic tissue from ME2082b **(Malignant Melanoma)**	1.2	0.8	1.3	1.0	4.3
6	OV2086: **Ovary tissue from FDA array**	0.7	0.7	1.7	1.3	4.4
7	BR10010: **Breast tissue** from FDA array	1.7	1.0	1.0	1.0	4.7
8	MS801: Non-neoplastic tissue from MS801 **(Mesothelioma)**	2.5	0.9	0.9	0.8	5.2
9	LY2086: **Lymph node tissue** from FDA array	2.0	0.0	1.7	1.7	5.4
10	LM803: **Lymph node tissue** from FDA array	2.0	0.0	1.7	1.7	5.4
11	BM483: **Bone marrow** from FDA array	2.0	0.3	1.3	2.0	5.6
12	LC20813: **Lung tissue** from FDA array	1.0	1.3	2.0	1.3	5.6
13	PA1921: **Normal pancreatic tissue** from FDA array	2.0	2.0	1.7	1.3	7.0
14	PA2081a: **Normal pancreatic tissue** from FDA array	2.0	2.0	1.7	1.3	7.0
15	CO1503: **Colon tissue** from FDA array	1.5	2.0	2.0	2.0	7.5
16	BCO3119: **Liver tissue** from FDA array	2.3	2.3	3.0	2.0	9.6

Color code for the single antigens and the summarized protein expression score: Expression levels of an individual tissue type around the median of all tissue types = white; expression elevated in comparison to the median = shades of red, the higher, the darker; expression decreased in comparison to the median = shades of green, the higher, the darker

TGF-Δ pathway protein expression appeared highest in liver tissue, followed by colon and pancreatic tissue. Thus, it seems that the gastrointestinal tract has particularly high TGF-Δ expression already in a non-cancerous setting. Squamous epithelia from head and neck had the lowest basal TGF-Δ expression. Other non-neoplastic tissue types with rather low TGF-Δ pathway protein expression were brain tissue, prostate tissue, skin tissue, ovary tissue and breast tissue (Table 2).

**Figure 1:**
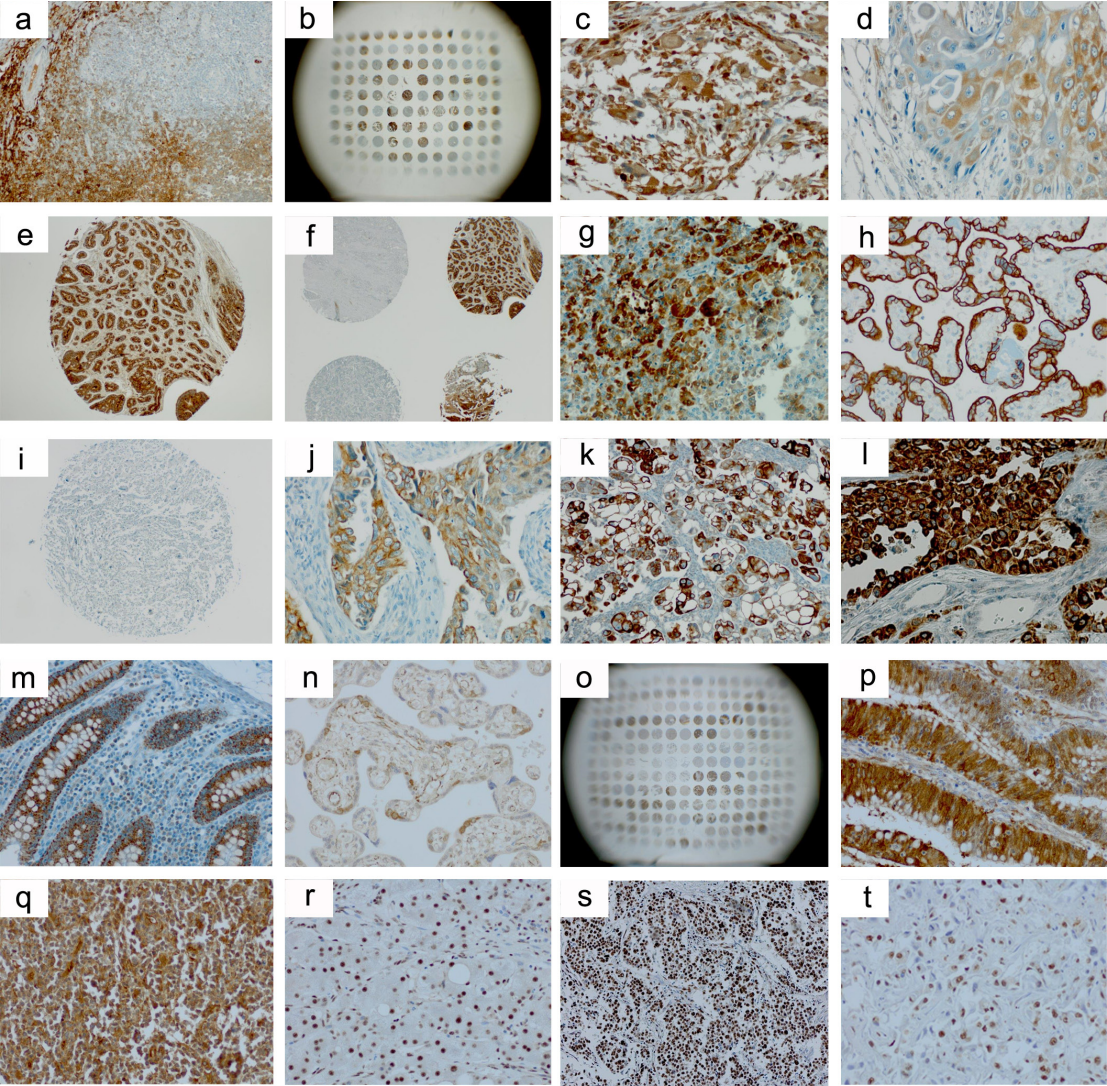
Selected immunohistochemical images from the stainings of the tissue arrays **a.**-**g.** TGF-Δ1 (Acris, #DM1047); **h.**-**m.**, TGF-Δ2 (Acris, #AP15815PU); **n.**-**q.**, TGF-Δ2 (Santa Cruz, #sc-90); **r.**-**t.**, p-Smad2/3 (Cell Signaling, #3101). **a.**, tonsil positive control; **b.**, BC03119 (liver), array overview; **c.**, GL803a (brain tumor), core C9; **d.**, HN483 (head and neck cancer), core B1; **e.**. PR8010 (prostate cancer), core C9; **f.**, PR8010 (prostate cancer), cores C/D8/9; **g.**, normal pancreatic tissue (FDA array); **h.**, plazenta positive control; **i.**, GL803a (brain tumor), core C9; **j.**, LC20831 (lung cancer), core C2; **k.**, MS801 (mesothelioma), core B5; **l.**, BR10010a (breast cancer), core D4; **m.**, normal colon tissue (FDA array); **n.**, plazenta positive control; **o.**, OV2086 (ovary cancer), array overview; **p.**, CO1503 (colon cancer), core C4; **q.**, LY2086 (lymphoma), core C8; **r.**, cirrothic liver positive control, **s.**, PA1921 (mid-advanced stage pancreatic cancer), core C5, **t.**, BC03119 (liver), core A7. There is notable interindividual heterogeneity of antigen expression also within the same tumor entity **b.**, **f.**, **o.** TGF-Δ2 immunostaining appears more specific with the TGF-Δ Acris **h.**-**m.** than with the TGF-Δ2 Santa Cruz **n.**-**q.** antibody and is comparably low in the brain tumor arrays **i.** Non-neoplastic tissues of the gastrointestinal tract **g.**, **m.** exhibit high TGF-Δ expression levels also outside of the neoplastic setting.

### Integrative analysis of TGF-Δ isoform expression and pathway activation in tumor and non-neoplastic tissues

We then aimed to compare the extent of TGF-Δ isoform expression and pathway activation in the tumor arrays with the staining results from the corresponding non-neoplastic tissues. Table 3 combines the summarized protein expression scores calculated from the mean protein expression levels. Tissue types were ranked according to their differences in expression levels between matching neoplastic and non-neoplastic tissues.

**Table 3:** Integrative analysis of TGF-Δ isoform expression and pathway activation in tumor and corresponding non-neoplastic tissues

difference tumor/nonneoplastic	non- neoplastic tissues		tumors	
summarized score			mean protein expression levels	
1.3	3.5	4.9	HN483: Multiple **head and neck cancer** with normal tissue array	1
1.3	4.7	6.0	BR10010: **Breast cancer** and matched metastatic carcinoma tissue array	2
1.2	5.2	6.4	M5801: **Mesothelioma** tissue array with normal mesothelium	3
1.2	4.4	5.6	OV2086: **Ovary cancer** survey tissue array	4
1.0	3.7	4.7	GL803a: **Brain tumor** and adjacent tissue array	5
0.7	4.0	4.7	PR8010: **Prostate cancer** tissue array	6
0.3	5.6	5.9	BM483: Tumor tissue array (**Myeloma**)	7
0.1	3.7	3.8	GL2083a: **Brain tumor** tissue array	8
0.0	5.6	5.6	LC20813: **Lung cancer** tissue array	9
−0.1	5.4	5.3	LY2086: **Lymphoma** tumor tissue array	10
−0.3	4.3	4.0	ME2082b: **Malignant Melanoma** tissue array	11
−1.0	5.4	4.4	LM803: **Lymphoma** and normal lymph node tissue array	12
−1.8	7.0	5.2	PA1921: Mid-advanced stage **pancreatic cancer** tissue array	13
−2.3	7.0	4.7	PA2081a: **Pancreatic** disease spectrum tissue array	14
−2.7	7.5	4.8	C01503: **Colon Cancer** tissue array	15
−4.1	9.6	5.5	BCO3119: **Liver carcinoma** and normal tissue	16

Tables are formatted according to a single array's deviation from the median of all arrays. For the tumors green designates comparably high and for the non-neoplastic tissues comparably low expression. For the differences between tumor and non-neoplastic tissues green designates high and red low expression in the tumors compared to the corresponding non-neoplastic tissues

Tumors with comparably high TGF-Δ isoform expression/pathway activation and comparably low TGF-Δ isoform expression/pathway activation in the corresponding non-neoplastic tissues were squamous cell carcinomas of the head and neck, mesothelioma and tumors of the female genital tract (breast and ovary cancer). Also brain tumors, prostate cancer and myeloma ranked rather high in this comparison. Due to high protein expression levels already in the non-neoplastic setting, tumors from the gastrointestinal tract (pancreatic, colon and liver cancer) ranked low in this specific comparison.

### Assessment of antibody specificity and assay validity

We next performed a thorough work-up for antibody specificity and assay validity. An additional TGF-Δ3 antibody was included for this analysis to detect all three TGF-Δ isoforms. By the suppliers, both TGF-Δ2 antibodies were described to react with the precursor and mature form of TGF-Δ2 and to cross-react to a lesser extent with TGF-Δ3 and not with TGF-Δ1. No statement was made on isoform specificity of the TGF-Δ1 antibody and TGF-Δ3 was considered to be isoform specific with no cross-reactivity at all. In our hands, using recombinant TGF-Δ1, -Δ2 and Δ3 protein we could not detect a signal with the TGF-Δ1 and the TGF-Δ2 (Acris) antibody on Western blots (not shown). However, the TGF-Δ2 (SC) and the TGF-Δ3 antibody appeared to specifically detect the respective recombinant proteins (Suppl. Figure 1).

We next used Panc-1 cells treated with TGF-Δ directed antisense oligonucleotides to further assess isoform-specific immunoreactivity. Cells were treated with LNA-modified antisense oligonucleotides gapmer according to the experimental conditions described in the Materials and Methods section. Efficacy of the oligonucleotide treatment was tested by determination of secreted TGF-Δ1 and TGF-Δ2 in cell supernatants by ELISA (Table 4a). The results of the ELISA confirmed the expected downregulation of secreted TGF-Δ1 and -Δ2 by the respective oligonucleotides (Table 4b). We then generated pellets from the antisense oligonucleotide treated cells, processed them to paraffin blocks and stained them by immunocytochemistry. The antibody panel was again extended to a total of two different TGF-Δ1, 4 different TGF-Δ2 and 2 different TGF-Δ3 antibodies including the antibodies that had been employed for staining the tissue arrays. However, for none of the antibodies we could observe relevant differences in staining intensity or number of immunostained cells comparing the different treatment conditions (Suppl. Figure 2).

**Table 4:** Panc-1 cells treated with TGF-Δ antisense oligonucleotides

a)				
	TGF-Δ1		TGF-Δ2	
	mean	SD	mean	SD
Vehicle	333	42	1323	157
ASPH_0047	704	93	181	32
ASPH_0047+1047	85	52	218	21
ASPH_1047	51	50	1188	34
ASPH_1132	50	77	91	7
LNA-scrambled	592	42	1590	98

b)				
Sample #	Treatment	Downregulation expected of:
05630001H	Vehicle	none
05630002H	Vehicle	none
05630004H	LNA-scrambled	none
05630005H	LNA-scrambled	none
05630007H	ASPH_0047	TGF- Δ 2
05630008H	ASPH_0047	TGF- Δ 2
05630010H	ASPH_1047	TGF- Δ 1
05630011H	ASPH_1047	TGF- Δ 1
0563001311	ASPH_0047_1047	TGF- Δ1 and TGF- Δ 2
05630014H	ASPH_0047_1047	TGF- Δ 1 and TGF- Δ 2

a) TGF-Δ protein levels determined in Panc-1 cell supernatants by ELISA. Mean and SD (in pg/ml) of triplicate measurements are shown. b) Overview of the tissue blocks generated that should allow for assessing the differential expression of TGF-Δ isotypes by means of immunocytochemistry

**Figure 2:**
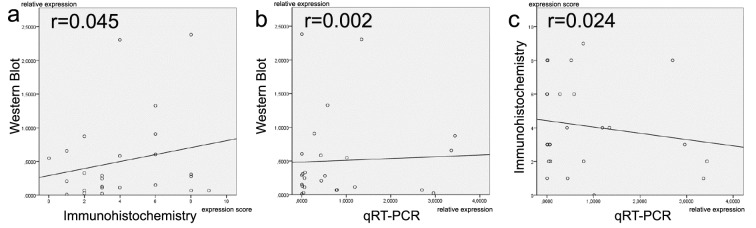
Comparison of TGF-Δ2 expression levels between different methodical platforms (Western blot, immunohistochemistry and qRT-PCR) There is a lack of linear correlation in all comparisons **a.**-**c.** Proteins and mRNAs were extracted from the same cell populations.

Finally, we aimed to perform cross-platform comparisons for TGF-Δ2 expression by using immunohistochemistry, Western blotting and qRT-PCR (Acris antibody and Δ2-specific primers). The comparisons were made in 32 anaplastic glioma tissues samples from which RNA- and protein isolates had been obtained from the same cell fraction to allow best possible comparison between the different platforms. There was no linear correlation between either Western blot or immunohistochemistry (*r* = 0.045), Western Blot or qRT-PCR (*r* = 0.002), and immunohistochemistry or qRT-PCR (*r* = 0.024) (Figure 2).

## DISCUSSION

Scattered data on the expression or activation of single TGF-Δ pathway components in specific tumors types have been published over the past years in various publications.[4, 14] Our comprehensive investigation was designed to compare the expression of the TGF-Δ-isoforms and phosphorylation of downstream pathway components across a large spectrum of human neoplastic and non-neoplastic tissue samples by using a standardized immunohistochemical approach. In order to identify those cancer patients that have significant presence of individual TGF-β isoforms and have distinct pathway activation and consequently may more likely benefit from a TGF-Δ-isoform directed therapeutic intervention, we screened 1638 cancer samples from 13 different tumor types. This data is fully disclosed in this publication and may serve as a valuable reference resource for researchers in different context situations.

A specific challenge to our endeavor was to integrate the enormous amount of data obtained by evaluating the tissue arrays and to come to meaningful conclusions that might help to recognize patterns of target expression. We here propose a priority list on tumors that in comparison between the different entities had peak TGF-Δ expression/activation levels and in addition showed relatively low signaling pathway activation in corresponding non-neoplastic tissue samples (Tables 1, 2 and 3). As such, our data might aid for decision-making in the design of future clinical trials with TGF-Δ directed antisense molecules.

In contrast to this putative clearness of the results, there are also a number of limitations to the interpretation of the data. First, it has to be discussed why some tumor types performed differently depending on which parameters were used for comparison. Myeloma and liver cancer tissues, for example, had high mean expression scores and also ranked high in the percentage of positive tumors. Nevertheless, in the comparison including the only weakly stained tumor cores (% positive tumors) these arrays ranked in less prominent positions. This discrepancy may be due to the fact that other arrays contained a higher number of weakly stained tumors while myeloma and liver cancer tissues staining for TGF-Δ antigens had either no or strong and convincing immunoreactivity. The differentiated way at looking at the summarized data from multiple perspectives (Tables 1, 2 and 3) might therefore shed light on the complexity of TGF-Δ signaling in the different tumor entities.

Another notable observation was that different arrays representing the same tumor type (the two pancreatic cancer arrays and the lymphoma arrays) performed quite differently in our analysis. Here, the most likely explanation is the different spectrum of tumor subentities represented on the respective arrays. As to the lymphoma arrays, on LY2086 a magnitude of diffuse large B-cell non-Hodgkin's lymphomas are spotted, while on LM803 mainly Hodgkin's lymphomas are represented. It thus appears that diffuse large B-cell non-Hodgkin's lymphomas, the main subtype of aggressive lymphomas, have higher TGF-Δ pathway activation. The situation becomes somewhat more complex when comparing both pancreatic cancer tissue arrays. Here, the inflammatory samples on PA2081a were not included in the score and are therefore not causative for the difference. Instead, it seems that the stage of disease makes the difference: While PA2081a contains pancreatic cancer of all stages including very early ones, PA1921 rather homogenously represents a mid-advanced stage of the disease with local growth outside the pancreas but no invasion into large blood vessels/major nerves or spread to distant sites. This mid-advanced stage of the disease appears to correlate with a comparatively high TGF-Δ isoform expression/pathway activation, in line with published literature indicating the increasing role of TGF-β in tumor progression.[15].

While the comparison of TGF-Δ expression and pathway activation between tumors and corresponding non-neoplastic tissues (Table 3) may shed light on the extent of protein expression caused by the cancerous setting, these findings should not be over-interpreted. One interesting aspect in this comparison pertains to the brain tumors. They demonstrate astonishingly low TGF-Δ expression/activation levels but there is an equally low TGF-Δ expression in non-neoplastic brain tissue. Thus, even low TGF-Δ pathway expression differences in these tumors might be “druggable” and of oncogenic relevance. On the other hand, the high expression/activation levels in non-neoplastic tissues of the gastrointestinal tract (liver, colon, pancreas) do not necessarily exclude that in the corresponding neoplastic tissues there still might be a relevant oncogenic effect of TGF-Δ signaling. Most importantly in this context, there is the TGF-Δ pathway paradox to be mentioned that describes a possible diversity in TGF-Δ function[16-18]. Especially in benign epithelia and early-stage tumors, TGF-Δ can act as a potent inducer of growth arrest, while in advanced tumors TGF-Δ promotes tumor growth and progression. Thus, also in entities that have high TGF-Δ expression already in the non-neoplastic setting, TGF-Δ directed therapies in advanced cancers with TGF-Δ expression might be beneficial due to a switch in TGF-Δ function.

One of the most meaningful results of our analysis was that across all tumor entities TGF-Δ signaling was subject to a large inter-individual heterogeneity. The fraction of cancer patients expressing TGF-Δ1 and TGF-Δ2 at intermediate to high levels varied from 30 up to 60% at maximum. Thus, in the context of TGF-Δ targeted therapies a predictive testing procedure would be strongly recommendable for the design of future clinical trials. An immunohistochemical predictive test in terms of feasibility would be suited for broad acceptance as it can be easily performed in any pathology department. Yet, a suitable immunohistochemical test should ideally correlate with the expression on the mRNA level that reflects, in case of an antisense oligonucleotide, the presence of the drug's direct target. Our investigations have brought forth several distinct uncertainties in this regard. For some arrays there were large discrepancies between staining intensities for the two TGF-Δ2 antibodies (e.g. LY2086, GL803a, GL2083a). Usually, the Acris TGF-Δ2 antibody (staining with this antibody appeared to generate less background staining) provided lower staining scores than the Santa Cruz TGF-Δ2 antibody. Also, the astonishingly low expression levels in brain tumors did not fit with the existing literature, which in part is also based on immunohistochemistry, but includes additional RNA-based analyses.[8, 9]

We thus further assessed antibody specificity and assay validity of the four antibodies used for staining the arrays and extended our antibody panel to an even larger selection of commonly used commercially available antibodies. Disturbingly, on cell pellets treated with different TGF-Δ antisense oligonucleotides (Table 4) none of the antibodies could detect any meaningful differences (Suppl. Figure 2). Also, in cross-platform validations using the TGF-Δ2 Acris antibody (that appeared most specific in the immunhistochemical stains) we could not detect any linear correlation between immunohistochemistry, Western blotting or qRT-PCR (Figure 2). In line with these findings, a ranking of tumor entities based on TGF-Δ ligand mRNA expression using publically available data from the Gene Expression Omnibus (GEO) repository (Suppl. Figure 3) did not well overlap with our protein expression (immunohistochemistry)-based ranks of the respective tumor types. Nevertheless, the GEO data have to be interpreted with caution as far fewer patients per tumor entity were contained than on our tissue arrays (e.g. 10 melanomas on the mRNA array vs. 128 melanomas on our tissue array) and mRNA expression intensities were prone to high mean variations (box plots, Suppl. Figure 3).

Although RNA- and protein-levels do not have to correlate in linear fashion, these results dampen the enthusiasm about immunohistochemistry as a valid tool to assess TGF-Δ-isoform expression/pathway activation with great precision. Nevertheless, though our laboratories are highly experienced in optimizing immunocytochemical assays there are limitations to the immunocytochemical staining of cell pellets that might in part explain these results. Western blots (as published by the suppliers) usually demonstrate good isoform reactivity for TGF-Δ2 antibodies (with some crossreactivity for TGF-Δ3). We could confirm isoform specificity for the TGF-Δ2(SC) antibody and additionally demonstrate isoform-specificity for the TGF-Δ3(Acris) antibody (Suppl. Table 1) which is contrary to the negative results from the cell pellets. Also, in terms of the discrepancies between protein and mRNA expression levels in the cross-platform validations there are factors apart from merely technical aspects that might obscure correlations. Differences in half-lives and modes of degradation might add to explain for non-uniform RNA and protein levels. Nevertheless, as far as therapeutic strategies are concerned that rely on antisense techniques, proof of the target (mRNA) knock-down appears essential and immunohistochemistry alone might not be a sufficient tool in this regard.

Taken together, this analysis presents the largest standardized screen on TGF-Δ isoform expression/pathway activation by using immunohistochemistry on human cancer and non-neoplastic tissue arrays so far performed. We find TGF-Δ1 and -Δ2 expressed to substantial amounts in multiple cancers, widening the options for TGF-Δ isoform directed therapies. A generally high p-Smad2/3 activity suggests a major role of the pathway in many cancer types. TGF-Δ antigens appear to be expressed in an individual manner pointing towards a need for patient preselection when considering TGF-β-isoform specific treatment. However, with the currently available antibodies uncertainties regarding assay validity and TGF-Δ isoform specificity do exist, and consequently the potential predictive suitability of immunohistochemistry will require further validation. Thus, patient stratification for clinical trials with TGF-β antisense molecules should not be solely based upon TGF-Δ immunohistochemistry results but should encompass investigations on mRNA levels and possibly other existing pathway activity assessments.

## MATERIALS AND METHODS

### Tissue arrays

Tissue arrays were obtained from US Biomax, Inc. (Rockville, MD, USA). The arrays covered a broad spectrum of main cancer entities from such diverse organ systems as the male and female genital tract, the respiratory system, gastrointestinal tumors, squamous cell carcinomas (head and neck cancer), neuroectodermal tumors (comprising malignant melanoma and brain cancers) as well as tumors of the lymphatic system. Taken together 16 tumor tissue arrays comprising 13 different tumor types were analyzed: 1. #BR10010: Breast cancer and matched metastatic carcinoma tissue array, 2. #OV2086: Ovary cancer survey tissue array, 3. #PR8010: Prostate cancer tissue array, 4. #LC20813: Lung cancer tissue array, 5. #MS801: Mesothelioma tissue array with normal mesothelium, 6a. #PA1921: Mid-advanced stage pancreatic cancer tissue array, 6b. #PA2081a: Pancreatic disease spectrum tissue array, 7. #BC03119: Liver carcinoma and normal tissue, 8. #CO1503: Colon Cancer tissue array, 9. #HN483: Multiple head and neck cancer with normal tissue array, 10. #ME2082b: Malignant Melanoma tissue array, 11a. #GL803a: Brain tumor and adjacent tissue array, 11b. #GL2083a: Brain tumor tissue array, 12a. #LY2086: Lymphoma tumor tissue array, 12b. #LM803: Lymphoma and normal lymph node tissue array, 13. #BM483: Tumor tissue array (Myeloma). The arrays contained different numbers of cores (ranging from 48-208), either spotted as single or duplicate cores per patient. More specific information on the single entities represented on each array is supplied in Suppl. Tables 1 and 2. Some arrays -aside from primary tumor tissues- included inflammatory, metastatic or non-neoplastic control tissue enabling additional comparisons.

Apart from the 16 tumor arrays we included an FDA array (US Biomax, Inc.) in our analysis that contained healthy tissues from multiple organ systems: 14. #FDA808ci-1_FDA normal organ tissue array. This array contained non-neoplastic tissue samples corresponding to the neoplastic tissues studied on the other arrays. As such, TGF-Δ signaling pathway components could not only be studied in comparison between different tumor entities but also in relation to the expression levels in corresponding non-neoplastic tissues.

### Immunhistochemistry

Immunohistochemical staining of the tissue arrays was performed according to standard protocols.[19] In brief, slides were deparaffinized and, after heat-induced antigen retrieval and blocking, incubated with the primary antibody. The primary antibodies and staining conditions were as follows: *TGF-Δ1*: Acris, #DM1047, dilution 1:100, pretreatment in EDTA-buffer for 36 min.; *TGF-Δ2*: Acris, #AP15815PU-S, 1:25, EDTA-buffer for 36 min.; Santa Cruz, #sc-90, 1:25, citrate-buffer for 30 min.; *p-Smad2/3*: Cell Signaling, #3101, 1:200, citrate-buffer for 20 min. Immunoreactivity was detected using 3,3′-diaminobenzidine tetrahydrochloride (DAB) as a chromogen. Stainings for p-Smad2/3 and TGF-Δ2 (for the Santa Cruz antibody) were performed manually using the EnVision^TM^ Detection System (Dako, # K406511-2). Stainings for TGF-Δ1 and TGF-Δ2 (Acris antibody) were performed on the Benchmark Ultra Autostainer (Ventana/Roche, Mannheim, Germany) using the reagents and detection systems supplied by the vendor. As positive controls we used human placenta tissue for both TGF-Δ2 antibodies, human tonsil tissue for TGF-Δ1 staining and cirrhotic liver tissue for p-Smad2/3 detection.

### Evaluation of array immunohistochemical staining and statistical analyses

To assess the extent of staining for each individual tissue core on the arrays, a semiquantitative score ranging from 0 (no staining at all) to 3 (strong staining in the majority of the core) was employed (Suppl. Table 1). Slides were scored by two observers (M.J.R. and M.H.). Statistical analyses assessed means and standard deviations of the expression of the individual antigens as well as the percentage of positive tumors (score >0) and the percentage of relevantly positive tumors (score >1) in the different tumor entities. Where applicable, expression differences between individual sample subgroups (such as primary tumors and metastasis or neoplastic and non-neoplastic samples) were assessed for significance using student's t-test analyses (Suppl. Table 2). A subsequent comparative and integrative analysis of all tissue arrays was performed. To compare the extent of expression (either number of positive patients or mean expression scores) between the different arrays, the median expression value of each individual antigen was calculated across all arrays and the individual arrays were then ranked according to their deviation from that median expression. Expression differences were visualized on a color scheme and tables formatted accordingly (Tables 1, 2 and 3).

### Experiments performed for assessing antibody specificity and assay validity

TGF-Δ isoform reactivity was assessed by Western blotting using recombinant TGF-Δ1 (#100-21/2μg), TGF-Δ2 (#100-35/1μg) and TGF-Δ3 (#100-36E/2μg) protein (PeproTech, Rocky Hill, NJ, USA). Western blotting was performed according to standard protocols by detection of chemiluminescence.[19] In addition to the antibodies that were used for staining of the arrays we included a TGF-Δ3 antibody (#AP15833PU; Acris antibodies, Herford, Germany).

In a second approach, we used Panc-1 cells treated with TGF-Δ directed antisense oligonucleotides to assess isoform-specific immunoreactivity. In short, 4×10^5^ cells of the human pancreatic cancer cell line Panc-1 were seeded on 10 cm dishes per condition in cell culture medium containing either vehicle (saline) or LNA-modified antisense oligonucleotides. The following 6 experimental conditions were applied: 1) Vehicle-treated cells; 2) Panc-1 cells treated with 10-μM LNA-scrambled (control oligonucleotide); 3) Panc-1 cells treated with 10-μM ASPH_0047 (LNA-modified antisense oligonucleotide gapmer selectively targeting TGF-β2 mRNA); 4) Panc-1 cells treated with 10-μM ASPH_1047 (LNA-modified antisense oligonucleotide gapmer selectively targeting TGF-β1 mRNA); 5) Panc-1 cells treated with combination of ASPH_1047 and ASPH_0047 (10 μM, each); 6) Panc-1 cells treated with 10-μM ASPH_1132 (LNA-modified antisense oligonucleotide gapmer targeting all 3 TGF-β isoforms, TGF-β1, -β2 and -β3 mRNA). Each test was performed in triplicate. After incubation for 3 days in a cell culture incubator, cell culture supernatant was removed and replaced by fresh antisense oligonucleotide-containing cell culture medium. Four days after start of the second treatment, cell supernatants were removed for analysis of secreted TGF-Δ1 and -Δ2 protein by ELISA. For TGF-Δ2, the human TGF-Δ2 Quantikine ELISA Kit (#DB250; R&D Systems, Minneapolis, MN) and for TGF-Δ1, the human TGF-Δ1 Quantikine ELISA Kit (#DB100B; R&D Systems) were used. In addition, cell pellets were generated and processed to paraffin blocks that were utilized for immunocytochemistry. The antibody panel again was extended by the following antibodies: TGF-Δ1, #MAB240, R&D Systems; TGF-Δ2, #MAB612, R&D Systems; TGF-Δ2, #ab36495, Abcam, Cambridge, UK; TGF-Δ3, #HPA27923, Sigma, St. Louis, MO. As such, in this experiment 2 different TGF-Δ1, 4 different TGF-Δ2 and 2 different TGF-Δ3 antibodies were used. The staining conditions are available on request.

Finally, we aimed to perform cross-platform comparisons of TGF-Δ2 expression by using immunohistochemistry, Western blotting and qRT-PCR. We used the TGF-Δ2 Acris antibody (#AP15815PU-S) and designed Δ2-specific primers (for: CACCATAAAGACAGGAACCTG; rev: GGAGGTGCCATCAATACCTGC, PCR conditions available on request). The comparison was made in 32 anonymized tissues samples from patients with anaplastic gliomas (29 glioblastoma, WHO grade IV and 3 anaplastic astrocytoma, WHO grade III; Departments of Neuropathology and Neurosurgery Regensburg). RNA- and protein isolates were obtained from the same cell fraction to allow best possible comparison between the different platforms. FFPE material used for immunohistochemistry was from tissue fractions adjacent to the frozen material that was used for RNA and protein extraction. Methods were performed as described before. Evaluation of immunohistochemistry was semi-quantitative and for this purpose with a composite numerical score based on the percentage of positive stained tumor cells multiplied by staining intensity, potentially ranging from 0 to 12. We reasoned that such a score would be particularly suited for the comparison to quantitative lysate-based approaches such as Western blot and qRT-PCR. The percentage of labeled cells was scored as follows: 0 (no or minimal reactivity, similar to nonneoplastic brain tissue), 1 (<10%), 2 (10-50%), 3 (50-90%), 4 (>90%). Staining intensity was graded as 0 (negative), 1 (weak), 2 (moderate), or 3 (strong).

## SUPPLEMENTARY MATERIAL FIGURES AND TABLES


