# Supplementary material for: TGF-Δ isoforms in cancer: Immunohistochemical expression and Smad-pathway-activity-analysis in thirteen major tumor types with a critical appraisal of antibody specificity and immunohistochemistry assay validity
Source: Oncotarget. 2015 Sep 22;6(29):26770–81. doi: 10.18632/oncotarget.5780 (PMC4694951; doi:10.18632/oncotarget.5780)
Supplement: Supplementary file 1 [file oncotarget-06-26770-s001.pdf]

TGF-ß isoforms in cancer: Immunohistochemical expression- and Smad-pathway-activity-analysis in thirteen major tumor types with a critical appraisal of antibody specificity and immunohistochemistry assay validity

Supplementary Material

Suppl. Table 1. Raw data of the staining of the tissue arrays. This table reports immunohistochemistry scores for each individual core of the 16 tumor arrays and the non-neoplastic tissue array. Arrays were stained with antibodies against TGF-ß1 and p-Smad2/3 as well as two different TGF-ß2 antibodies (Acris and Santa Cruz, SC). n.a. = not applicable, n.d. = not done.

1. BR10010: ***Breast cancer*** *and matched metastatic carcinoma tissue array*

position	sex	age	organ	pathology	type	TGFß1	TGFß2 (Acris)	TGFß2 (SC)	p-SMAD 2/3
A1	F	59	Breast	Invasive ductal carcinoma	Malignant	1	1	0	1
A2	F	66	Breast	Invasive ductal carcinoma	Malignant	2	2	1	2
A3	F	58	Breast	Invasive ductal carcinoma	Malignant	1	0	1	2
A4	F	55	Breast	Invasive ductal carcinoma	Malignant	2	n.a.	0	1
A5	F	39	Breast	Invasive ductal carcinoma	Malignant	n.a.	n.a.	0	2
A6	F	70	Breast	Invasive ductal carcinoma	Malignant	n.a.	n.a.	1	2
A7	F	52	Breast	Invasive ductal carcinoma	Malignant	n.a.	1	1	3
A8	F	72	Breast	Invasive ductal carcinoma	Malignant	n.a.	1	1	1
A9	F	60	Breast	Invasive ductal carcinoma	Malignant	n.a.	n.a.	0	1
A10	F	55	Breast	Invasive ductal carcinoma	Malignant	n.a.	n.a.	0	1
B1	F	28	Breast	Invasive ductal carcinoma	Malignant	1	2	1	1
B2	F	56	Breast	Invasive ductal carcinoma	Malignant	2	2	1	1
B3	F	40	Breast	Invasive ductal carcinoma	Malignant	1	2	1	2
B4	F	51	Breast	Invasive ductal carcinoma	Malignant	1	2	2	2
B5	F	40	Breast	Invasive ductal carcinoma	Malignant	1	1	2	1
B6	F	42	Breast	Invasive ductal carcinoma	Malignant	1	0	1	1
B7	F	41	Breast	Invasive ductal carcinoma	Malignant	1	1	1	2
B8	F	48	Breast	Invasive ductal carcinoma	Malignant	1	2	2	2
B9	F	52	Breast	Invasive ductal carcinoma (sparse)	Malignant	1	0	1	1
B10	F	59	Breast	Invasive ductal carcinoma	Malignant	0	n.a.	0	1
C1	F	42	Breast	Invasive ductal carcinoma	Malignant	3	1	1	2
C2	F	31	Breast	Invasive ductal carcinoma	Malignant	1	2	2	1
C3	F	53	Breast	Invasive ductal carcinoma	Malignant	1	2	2	2
C4	F	48	Breast	Invasive ductal carcinoma	Malignant	3	1	2	1
C5	F	58	Breast	Invasive ductal carcinoma	Malignant	2	1	1	2
C6	F	46	Breast	Invasive ductal carcinoma	Malignant	2	1	2	2
C7	F	80	Breast	Invasive ductal carcinoma	Malignant	1	3	3	2
C8	F	52	Breast	Invasive ductal carcinoma	Malignant	2	2	1	1

C9	F	44	Breast	Invasive ductal carcinoma	Malignant	2	1	1	3
C10	F	61	Breast	Invasive ductal carcinoma	Malignant	2	0	0	0
D1	F	56	Breast	Invasive ductal carcinoma	Malignant	3	2	2	2
D2	F	53	Breast	Invasive ductal carcinoma	Malignant	1	2	1	1
D3	F	75	Breast	Invasive ductal carcinoma	Malignant	1	2	3	3
D4	F	53	Breast	Invasive ductal carcinoma	Malignant	2	3	3	2
D5	F	50	Breast	Invasive ductal carcinoma	Malignant	3	1	2	3
D6	F	49	Breast	Invasive ductal carcinoma	Malignant	1	2	2	3
D7	F	49	Breast	Invasive ductal carcinoma	Malignant	1	1	2	2
D8	F	47	Breast	Invasive ductal carcinoma	Malignant	1	1	1	2
D9	F	39	Breast	Invasive ductal carcinoma	Malignant	3	1	1	2
D10	F	57	Breast	Invasive ductal carcinoma	Malignant	3	2	1	1
E1	F	50	Breast	Invasive ductal carcinoma	Malignant	2	2	2	2
E2	F	42	Breast	Invasive ductal carcinoma	Malignant	1	1	2	2
E3	F	48	Breast	Invasive ductal carcinoma	Malignant	3	1	1	1
E4	F	53	Breast	Invasive ductal carcinoma	Malignant	1	2	2	1
E5	F	33	Breast	Invasive ductal carcinoma	Malignant	1	2	2	3
E6	F	53	Breast	Invasive ductal carcinoma	Malignant	3	1	1	1
E7	F	49	Breast	Invasive micropapillary carcinoma	Malignant	2	1	2	2
E8	F	78	Breast	Invasive lobular carcinoma	Malignant	2	1	1	1
E9	F	39	Breast	Invasive lobular carcinoma	Malignant	2	1	1	1
E10	F	45	Breast	Neuroendocrine carcinoma	Malignant	3	1	0	0
F1	F	59	Lymph node	Metastatic carcinoma from breast (lymph node tissue)	Metastasis	1	3	3	3
F2	F	66	Lymph node	Metastatic carcinoma from breast	Metastasis	2	3	2	2
F3	F	58	Lymph node	Metastatic carcinoma from breast	Metastasis	1	3	2	3
F4	F	55	Lymph node	Metastatic carcinoma from breast	Metastasis	2	0	1	2
F5	F	39	Lymph node	Metastatic carcinoma from breast	Metastasis	1	2	1	3
F6	F	70	Lymph node	Metastatic carcinoma from breast	Metastasis	2	2	2	1
F7	F	52	Lymph node	Metastatic carcinoma from breast	Metastasis	2	1	1	2
F8	F	72	Lymph node	Metastatic carcinoma from breast	Metastasis	1	1	2	2
F9	F	60	Lymph node	Metastatic carcinoma from breast	Metastasis	2	1	2	2
F10	F	55	Lymph node	Metastatic carcinoma from breast	Metastasis	2	1	1	2
G1	F	28	Lymph node	Metastatic carcinoma from breast	Metastasis	2	1	3	2
G2	F	56	Lymph node	Metastatic carcinoma from breast	Metastasis	2	2	3	2
G3	F	40	Lymph node	Metastatic carcinoma from breast	Metastasis	1	2	1	1
G4	F	51	Lymph node	Metastatic carcinoma from breast	Metastasis	1	2	1	1
G5	F	40	Lymph node	Metastatic carcinoma from breast	Metastasis	1	3	2	2
G6	F	42	Lymph node	Metastatic carcinoma from breast	Metastasis	2	1	0	1
G7	F	41	Lymph node	Metastatic carcinoma from breast	Metastasis	3	2	2	2
G8	F	48	Lymph node	Metastatic carcinoma from breast	Metastasis	1	2	2	2

G9	F	52	Lymph node	Metastatic carcinoma from breast	Metastasis	1	2	1	2
G10	F	59	Lymph node	Metastatic carcinoma from breast	Metastasis	2	2	2	3
H1	F	42	Lymph node	Metastatic carcinoma from breast	Metastasis	2	1	1	3
H2	F	31	Lymph node	Metastatic carcinoma from breast	Metastasis	2	2	3	2
H3	F	53	Lymph node	Metastatic carcinoma from breast	Metastasis	1	2	2	3
H4	F	48	Lymph node	Metastatic carcinoma from breast	Metastasis	1	3	2	2
H5	F	58	Lymph node	Metastatic carcinoma from breast	Metastasis	2	1	1	3
H6	F	46	Lymph node	Metastatic carcinoma from breast	Metastasis	2	2	2	3
H7	F	80	Lymph node	Metastatic carcinoma from breast	Metastasis	3	2	3	2
H8	F	52	Lymph node	Metastatic carcinoma from breast	Metastasis	1	3	1	2
H9	F	44	Lymph node	Metastatic carcinoma from breast	Metastasis	1	1	1	3
H10	F	61	Lymph node	Metastatic carcinoma from breast	Metastasis	2	1	1	1
I1	F	56	Lymph node	Metastatic carcinoma from breast	Metastasis	1	2	2	0
I2	F	53	Lymph node	Metastatic carcinoma from breast	Metastasis	2	3	3	1
I3	F	75	Lymph node	Metastatic carcinoma from breast	Metastasis	1	2	2	2
I4	F	53	Lymph node	Metastatic carcinoma from breast	Metastasis	1	3	2	2
I5	F	50	Lymph node	Metastatic carcinoma from breast	Metastasis	1	1	2	2
I6	F	49	Lymph node	Metastatic carcinoma from breast	Metastasis	2	1	1	2
I7	F	49	Lymph node	Metastatic carcinoma from breast	Metastasis	1	1	2	1
I8	F	47	Lymph node	Metastatic carcinoma from breast	Metastasis	2	1	2	2
I9	F	39	Lymph node	Metastatic carcinoma from breast	Metastasis	2	1	1	2
I10	F	57	Lymph node	Metastatic carcinoma from breast	Metastasis	1	0	1	1
J1	F	50	Lymph node	Metastatic carcinoma from breast	Metastasis	2	2	1	0
J2	F	42	Lymph node	Metastatic carcinoma from breast	Metastasis	1	2	2	0
J3	F	48	Lymph node	Metastatic carcinoma from breast	Metastasis	2	0	1	1
J4	F	53	Lymph node	Metastatic carcinoma from breast	Metastasis	2	1	1	2
J5	F	33	Lymph node	Metastatic carcinoma from breast	Metastasis	2	n.a.	1	2
J6	F	53	Lymph node	Metastatic carcinoma from breast (sparse)	Metastasis	2	n.a.	2	3
J7	F	49	Lymph node	Metastatic carcinoma from breast	Metastasis	1	n.a.	2	2
J8	F	78	Lymph node	Metastatic carcinoma from breast	Metastasis	1	n.a.	2	2
J9	F	39	Lymph node	Metastatic carcinoma from breast	Metastasis	1	n.a.	1	2
J10	F	45	Lymph node	Metastatic carcinoma from breast	Metastasis	3	n.a.	0	2

*2. OV2086: **Ovary cancer** survey tissue array*

position	sex	age	organ	pathology	type	TGFß1	TGFß2 (Acris)	TGFß2 (SC)	p-SMAD 2/3
A1	F	50	Ovary	Serous papillary adenocarcinoma	Malignant	1	2	1	0
A2	F	50	Ovary	Serous papillary adenocarcinoma	Malignant	2	2	2	1
A3	F	45	Ovary	Serous adenocarcinoma	Malignant	n.a.	n.a.	n.a.	n.a.
A4	F	45	Ovary	Serous adenocarcinoma	Malignant	3	1	0	1
A5	F	58	Ovary	Serous papillary adenocarcinoma	Malignant	1	2	1	2
A6	F	58	Ovary	Serous papillary adenocarcinoma	Malignant	1	2	1	1
A7	F	34	Ovary	Serous papillary adenocarcinoma	Malignant	1	2	1	1
A8	F	34	Ovary	Serous papillary adenocarcinoma	Malignant	1	2	2	0
A9	F	53	Ovary	Serous adenocarcinoma with necrosis	Malignant	1	0	3	0
A10	F	53	Ovary	Serous adenocarcinoma with necrosis	Malignant	1	n.a.	2	0
A11	F	40	Ovary	Serous papillary adenocarcinoma (sparse)	Malignant	2	n.a.	0	0
A12	F	40	Ovary	Serous papillary adenocarcinoma	Malignant	1	n.a.	0	0
A13	F	26	Ovary	Serous adenocarcinoma	Malignant	1	n.a.	2	0
A14	F	26	Ovary	Serous adenocarcinoma	Malignant	1	n.a.	2	0
A15	F	60	Ovary	Serous papillary adenocarcinoma	Malignant	n.a.	n.a.	n.a.	n.a.
A16	F	60	Ovary	Serous papillary adenocarcinoma	Malignant	1	n.a.	1	0
B1	F	45	Ovary	Serous adenocarcinoma	Malignant	1	2	1	0
B2	F	45	Ovary	Serous adenocarcinoma	Malignant	1	2	1	1
B3	F	53	Ovary	Serous papillary adenocarcinoma	Malignant	1	2	3	2
B4	F	53	Ovary	Serous papillary adenocarcinoma	Malignant	1	2	3	2
B5	F	61	Ovary	Serous papillary adenocarcinoma	Malignant	3	3	2	1
B6	F	61	Ovary	Serous papillary adenocarcinoma	Malignant	3	3	1	2
B7	F	59	Ovary	Serous adenocarcinoma	Malignant	2	1	1	1
B8	F	59	Ovary	Serous adenocarcinoma	Malignant	n.a.	n.a.	n.a.	n.a.
B9	F	27	Ovary	Serous papillary adenocarcinoma	Malignant	1	0	1	1
B10	F	27	Ovary	Serous papillary adenocarcinoma	Malignant	1	0	1	1
B11	F	54	Ovary	Serous adenocarcinoma	Malignant	1	1	1	1
B12	F	54	Ovary	Serous adenocarcinoma	Malignant	2	1	1	1
B13	F	64	Ovary	Serous papillary adenocarcinoma	Malignant	1	1	2	0
B14	F	64	Ovary	Serous papillary adenocarcinoma	Malignant	1	1	2	0
B15	F	52	Ovary	Serous papillary adenocarcinoma	Malignant	1	2	2	0
B16	F	52	Ovary	Serous papillary adenocarcinoma	Malignant	1	2	1	0
C1	F	58	Ovary	Serous papillary adenocarcinoma	Malignant	3	2	1	1
C2	F	58	Ovary	Serous papillary adenocarcinoma	Malignant	3	2	1	1
C3	F	51	Ovary	Serous papillary adenocarcinoma	Malignant	1	2	3	2
C4	F	51	Ovary	Serous papillary adenocarcinoma	Malignant	1	2	3	2
C5	F	66	Ovary	Serous papillary adenocarcinoma	Malignant	1	1	2	3
C6	F	66	Ovary	Serous papillary adenocarcinoma	Malignant	1	1	2	2
C7	F	47	Ovary	Serous papillary adenocarcinoma	Malignant	1	1	2	2
C8	F	47	Ovary	Serous papillary adenocarcinoma	Malignant	1	1	3	2
C9	F	57	Ovary	Serous papillary adenocarcinoma	Malignant	1	2	0	3
C10	F	57	Ovary	Serous papillary adenocarcinoma	Malignant	1	2	1	3
C11	F	48	Ovary	Serous adenocarcinoma	Malignant	1	1	2	3
C12	F	48	Ovary	Serous adenocarcinoma	Malignant	2	1	2	3
C13	F	63	Ovary	Serous papillary adenocarcinoma	Malignant	2	0	2	3
C14	F	63	Ovary	Serous papillary adenocarcinoma	Malignant	n.a.	n.a.	n.a.	n.a.

C15	F	37	Ovary	Serous adenocarcinoma (sparse) with necrosis	Malignant	1	1	1	1
C16	F	37	Ovary	Serous adenocarcinoma with necrosis	Malignant	1	1	1	0
D1	F	58	Ovary	Serous adenocarcinoma	Malignant	1	n.a.	2	2
D2	F	58	Ovary	Serous adenocarcinoma	Malignant	1	3	2	2
D3	F	68	Ovary	Serous adenocarcinoma with necrosis	Malignant	1	2	2	2
D4	F	68	Ovary	Serous adenocarcinoma with necrosis	Malignant	1	2	2	2
D5	F	50	Ovary	Serous adenocarcinoma	Malignant	1	1	2	3
D6	F	50	Ovary	Serous adenocarcinoma with necrosis	Malignant	1	1	2	2
D7	F	36	Ovary	Serous adenocarcinoma	Malignant	1	1	2	1
D8	F	36	Ovary	Serous adenocarcinoma	Malignant	1	1	2	2
D9	F	54	Ovary	Serous adenocarcinoma	Malignant	3	1	2	2
D10	F	54	Ovary	Serous adenocarcinoma	Malignant	3	1	2	2
D11	F	58	Ovary	Serous adenocarcinoma with necrosis	Malignant	1	2	3	2
D12	F	58	Ovary	Serous adenocarcinoma with necrosis	Malignant	1	2	3	3
D13	F	41	Ovary	Serous papillary adenocarcinoma	Malignant	1	2	3	3
D14	F	41	Ovary	Serous papillary adenocarcinoma	Malignant	1	3	3	3
D15	F	57	Ovary	Serous papillary adenocarcinoma	Malignant	1	2	3	2
D16	F	57	Ovary	Serous papillary adenocarcinoma	Malignant	1	2	3	1
E1	F	50	Ovary	Serous adenocarcinoma	Malignant	1	1	1	2
E2	F	50	Ovary	Serous adenocarcinoma	Malignant	1	1	1	2
E3	F	59	Ovary	Serous adenocarcinoma	Malignant	1	2	2	2
E4	F	59	Ovary	Serous adenocarcinoma	Malignant	1	2	2	2
E5	F	44	Ovary	Serous adenocarcinoma	Malignant	1	1	3	1
E6	F	44	Ovary	Serous adenocarcinoma	Malignant	1	1	3	1
E7	F	53	Ovary	Serous adenocarcinoma	Malignant	2	2	3	2
E8	F	53	Ovary	Serous adenocarcinoma	Malignant	2	2	3	2
E9	F	52	Ovary	Serous adenocarcinoma (sparse) with necrosis	Malignant	2	1	1	1
E10	F	52	Ovary	Serous adenocarcinoma with necrosis	Malignant	2	1	2	2
E11	F	52	Ovary	Serous adenocarcinoma	Malignant	2	2	1	2
E12	F	52	Ovary	Serous adenocarcinoma	Malignant	2	2	2	2
E13	F	41	Ovary	Serous adenocarcinoma	Malignant	1	1	1	3
E14	F	41	Ovary	Serous adenocarcinoma	Malignant	1	1	1	3
E15	F	60	Ovary	Serous adenocarcinoma	Malignant	2	1	2	2
E16	F	60	Ovary	Serous adenocarcinoma	Malignant	3	2	2	1
F1	F	51	Ovary	Serous adenocarcinoma	Malignant	1	1	1	3
F2	F	51	Ovary	Serous adenocarcinoma	Malignant	1	1	1	3
F3	F	70	Ovary	Serous adenocarcinoma	Malignant	1	2	1	3
F4	F	70	Ovary	Serous adenocarcinoma	Malignant	2	2	1	3
F5	F	48	Ovary	Serous adenocarcinoma	Malignant	1	2	3	3
F6	F	48	Ovary	Serous adenocarcinoma	Malignant	1	2	2	2
F7	F	62	Ovary	Serous adenocarcinoma	Malignant	2	1	3	2
F8	F	62	Ovary	Serous adenocarcinoma	Malignant	2	1	3	2
F9	F	47	Ovary	Endometrioid adenocarcinoma	Malignant	1	1	1	2
F10	F	47	Ovary	Endometrioid adenocarcinoma	Malignant	1	1	1	2
F11	F	49	Ovary	Endometrioid adenocarcinoma with necrosis	Malignant	1	0	1	2
F12	F	49	Ovary	Endometrioid adenocarcinoma with necrosis	Malignant	1	0	1	1
F13	F	43	Ovary	Endometrioid adenocarcinoma	Malignant	2	1	1	2
F14	F	43	Ovary	Endometrioid adenocarcinoma	Malignant	2	1	1	1
F15	F	51	Ovary	Endometrioid adenocarcinoma	Malignant	1	1	1	2
F16	F	51	Ovary	Endometrioid adenocarcinoma	Malignant	2	1	1	2
G1	F	41	Ovary	Endometrioid adenocarcinoma (sparse)	Malignant	2	1	1	2

G2	F	41	Ovary	Endometrioid adenocarcinoma	Malignant	2	1	1	2
G3	F	43	Ovary	Endometrioid adenocarcinoma	Malignant	2	1	1	3
G4	F	43	Ovary	Endometrioid adenocarcinoma	Malignant	2	1	1	2
G5	F	55	Ovary	Endometrioid adenocarcinoma	Malignant	1	1	0	1
G6	F	55	Ovary	Endometrioid adenocarcinoma	Malignant	1	1	0	1
G7	F	53	Ovary	Endometrioid adenocarcinoma	Malignant	1	0	1	3
G8	F	53	Ovary	Endometrioid adenocarcinoma	Malignant	1	1	1	2
G9	F	63	Ovary	Mucinous adenocarcinoma	Malignant	1	0	0	1
G10	F	63	Ovary	Mucinous adenocarcinoma	Malignant	1	1	0	0
G11	F	60	Ovary	Mucinous adenocarcinoma (necrotic tissue)	Malignant	1	1	0	0
G12	F	60	Ovary	Mucinous adenocarcinoma	Malignant	1	1	1	1
G13	F	40	Ovary	Mucinous adenocarcinoma	Malignant	1	2	1	2
G14	F	40	Ovary	Mucinous adenocarcinoma (fibrous tissue and blood vessel)	Malignant	2	0	1	1
G15	F	45	Ovary	Mucinous adenocarcinoma (necrotic tissue)	Malignant	0	0	0	0
G16	F	45	Ovary	Mucinous adenocarcinoma	Malignant	1	2	1	1
H1	F	46	Ovary	Mucinous adenocarcinoma	Malignant	1	1	n.a.	0
H2	F	46	Ovary	Mucinous adenocarcinoma	Malignant	1	1	0	0
H3	F	48	Ovary	Mucinous adenocarcinoma	Malignant	1	1	1	2
H4	F	48	Ovary	Mucinous adenocarcinoma	Malignant	1	1	1	1
H5	F	49	Ovary	Mucinous adenocarcinoma	Malignant	1	1	1	2
H6	F	49	Ovary	Mucinous adenocarcinoma	Malignant	1	1	1	2
H7	F	65	Ovary	Mucinous papillary adenocarcinoma	Malignant	2	1	1	1
H8	F	65	Ovary	Mucinous papillary adenocarcinoma	Malignant	2	1	1	1
H9	F	67	Ovary	Mucinous adenocarcinoma (sparse)	Malignant	3	1	1	1
H10	F	67	Ovary	Mucinous adenocarcinoma	Malignant	3	1	1	1
H11	F	39	Ovary	Mucinous adenocarcinoma	Malignant	1	2	1	1
H12	F	39	Ovary	Mucinous adenocarcinoma	Malignant	1	2	1	2
H13	F	51	Ovary	Mucinous adenocarcinoma with necrosis	Malignant	2	1	0	1
H14	F	51	Ovary	Mucinous adenocarcinoma with necrosis	Malignant	2	1	0	1
H15	F	41	Ovary	Mucinous adenocarcinoma	Malignant	2	1	1	2
H16	F	41	Ovary	Mucinous adenocarcinoma	Malignant	1	1	0	1
I1	F	48	Ovary	Mucinous adenocarcinoma	Malignant	0	1	2	1
I2	F	48	Ovary	Mucinous adenocarcinoma	Malignant	0	1	2	1
I3	F	60	Ovary	Mucinous adenocarcinoma	Malignant	1	2	3	3
I4	F	60	Ovary	Mucinous adenocarcinoma	Malignant	1	2	3	3
I5	F	29	Ovary	Mucinous adenocarcinoma	Malignant	1	2	1	1
I6	F	29	Ovary	Mucinous adenocarcinoma	Malignant	1	2	1	2
I7	F	60	Ovary	Adult granulosa cell tumor	Malignant	2	2	3	3
I8	F	60	Ovary	Adult granulosa cell tumor	Malignant	2	2	3	3
I9	F	33	Ovary	Adult granulosa cell tumor	Malignant	1	1	0	2
I10	F	33	Ovary	Adult granulosa cell tumor	Malignant	1	1	1	3
I11	F	31	Ovary	Adult granulosa cell tumor	Malignant	1	0	1	2
I12	F	31	Ovary	Adult granulosa cell tumor	Malignant	1	0	1	2
I13	F	51	Ovary	Adult granulosa cell tumor	Malignant	2	1	0	1
I14	F	51	Ovary	Adult granulosa cell tumor	Malignant	n.a.	n	n.a.	n.a.
I15	F	54	Ovary	Adult granulosa cell tumor	Malignant	1	1	1	2
I16	F	54	Ovary	Adult granulosa cell tumor	Malignant	1	1	1	2
J1	F	65	Ovary	Adult granulosa cell tumor	Malignant	1	1	2	1
J2	F	65	Ovary	Adult granulosa cell tumor	Malignant	1	1	2	3
J3	F	49	Ovary	Adult granulosa cell tumor	Malignant	1	1	1	2
J4	F	49	Ovary	Adult granulosa cell tumor	Malignant	1	1	1	1

J5	F	59	Ovary	Adult granulosa cell tumor	Malignant	1	1	3	2
J6	F	59	Ovary	Adult granulosa cell tumor	Malignant	1	1	3	2
J7	F	60	Ovary	Adult granulosa cell tumor	Malignant	2	1	2	3
J8	F	60	Ovary	Adult granulosa cell tumor	Malignant	3	1	3	3
J9	F	59	Ovary	Adult granulosa cell tumor	Malignant	1	1	2	2
J10	F	59	Ovary	Adult granulosa cell tumor	Malignant	1	1	1	2
J11	F	39	Ovary	Granulosa-theca cell tumor	Malignant	1	2	2	2
J12	F	39	Ovary	Granulosa-theca cell tumor	Malignant	1	2	2	2
J13	F	49	Ovary	Granulosa-theca cell tumor	Malignant	1	2	3	2
J14	F	49	Ovary	Granulosa-theca cell tumor	Malignant	1	2	3	2
J15	F	59	Ovary	Granulosa-theca cell tumor	Malignant	1	1	1	2
J16	F	59	Ovary	Granulosa-theca cell tumor	Malignant	1	1	1	2
K1	F	50	Ovary	Theca cell tumor	Malignant	1	1	1	1
K2	F	50	Ovary	Theca cell tumor	Malignant	1	1	2	2
K3	F	48	Ovary	Theca cell tumor	Malignant	1	1	3	3
K4	F	48	Ovary	Theca cell tumor	Malignant	1	1	3	3
K5	F	44	Ovary	Theca cell tumor	Malignant	0	1	1	2
K6	F	44	Ovary	Theca cell tumor	Malignant	0	1	1	2
K7	F	15	Ovary	Endodermal sinus carcinoma	Malignant	1	1	1	1
K8	F	15	Ovary	Endodermal sinus carcinoma	Malignant	1	1	1	2
K9	F	26	Ovary	Endodermal sinus carcinoma	Malignant	1	1	0	2
K10	F	26	Ovary	Endodermal sinus carcinoma	Malignant	1	1	1	2
K11	F	20	Ovary	Endodermal sinus carcinoma	Malignant	1	1	1	1
K12	F	20	Ovary	Endodermal sinus carcinoma	Malignant	n.a.	1	n.a.	n.a.
K13	F	14	Ovary	Endodermal sinus carcinoma	Malignant	n.a.	n.a.	n.a.	n.a.
K14	F	14	Ovary	Endodermal sinus carcinoma	Malignant	1	n.a.	1	1
K15	F	32	Ovary	Endodermal sinus carcinoma	Malignant	1	n.a.	1	2
K16	F	32	Ovary	Endodermal sinus carcinoma	Malignant	1	n.a.	1	2
L1	F	62	Ovary	Squamous cell carcinoma from malignant transformation of teratoma	Malignant	1	1	2	1
L2	F	62	Ovary	Squamous cell carcinoma from malignant transformation of teratoma	Malignant	1	1	2	1
L3	F	26	Ovary	Undifferentiated carcinoma	Malignant	2	3	3	2
L4	F	26	Ovary	Undifferentiated carcinoma	Malignant	1	3	3	2
L5	F	50	Ovary	Squamous cell carcinoma from malignant transformation of teratoma	Malignant	1	0	1	2
L6	F	50	Ovary	Squamous cell carcinoma from malignant transformation of teratoma	Malignant	1	0	1	2
L7	F	54	Ovary	Clear cell carcinoma	Malignant	1	1	1	1
L8	F	54	Ovary	Clear cell carcinoma	Malignant	1	1	1	1
L9	F	57	Ovary	Transitional cell carcinoma	Malignant	2	n.a.	1	2
L10	F	57	Ovary	Transitional cell carcinoma	Malignant	2	n.a.	1	2
L11	F	30	Ovary	Sertoli cell tumor	Malignant	1	n.a.	2	2
L12	F	30	Ovary	Sertoli cell tumor	Malignant	1	n.a.	2	2
L13	F	49	Ovary	Sertoli cell tumor	Malignant	1	n.a.	1	0
L14	F	49	Ovary	Sertoli cell tumor	Malignant	1	n.a.	1	0
L15	F	59	Ovary	Sertoli-leydig cell tumor	Malignant	1	n.a.	1	3
L16	F	59	Ovary	Sertoli-leydig cell tumor	Malignant	1	n.a.	1	3
M1	F	72	Ovary	Dysgerminoma	Malignant	1	0	0	0
M2	F	72	Ovary	Dysgerminoma	Malignant	1	0	0	0
M3	F	19	Ovary	Dysgerminoma	Malignant	1	0	0	2
M4	F	19	Ovary	Dysgerminoma	Malignant	1	0	1	2
M5	F	13	Ovary	Mixed germ cell tumors	Malignant	1	n.a.	1	2

M6	F	13	Ovary	Mixed germ cell tumors	Malignant	1	n.a.	1	2
M7	F	14	Ovary	Gynandroblastoma	Malignant	1	n.a.	1	3
M8	F	14	Ovary	Gynandroblastoma	Malignant	1	n.a.	1	2
M9	F	16	Ovary	Immature teratoma	Malignant	1	n.a.	1	1
M10	F	16	Ovary	Immature teratoma ï¼ˆsparse)	Malignant	1	n.a.	1	1
M11	F	49	Ovary	Immature teratoma (mature teratoma)	Malignant	1	n.a.	1	0
M12	F	49	Ovary	Immature teratoma (mature teratoma)	Malignant	1	n.a.	1	0
M13	F	46	Ovary	Fibrosarcoma	Malignant	n.a.	n.a.	n.a.	1
M14	F	46	Ovary	Fibrosarcoma	Malignant	1	n.a.	1	1
M15	F	47	Ovary	Steroid cell tumor, not otherwise specified	Malignant	1	n.a.	2	2
M16	F	47	Ovary	Steroid cell tumor, not otherwise specified	Malignant	1	n.a.	2	2

3. PR8010: ***Prostate cancer*** *tissue array*

position	sex	age	organ	pathology	type	TGFß1	TGFß2 (Acris)	TGFß2 (SC)	p-SMAD 2/3
A1	M	71	Prostate	Adenocarcinoma	Malignant	0	0	1	1
A2	M	60	Prostate	Adenocarcinoma (sparse)	Malignant	1	n.a.	2	1
A3	M	74	Prostate	Adenocarcinoma	Malignant	0	n.a.	1	1
A4	M	66	Prostate	Adenocarcinoma (hyperplasia)	Malignant	0	n.a.	0	1
A5	M	70	Prostate	Adenocarcinoma	Malignant	n.a.	n.a.	1	1
A6	M	81	Prostate	Adenocarcinoma	Malignant	n.a.	n.a.	1	2
A7	M	59	Prostate	Adenocarcinoma (sparse)	Malignant	n.a.	n.a.	1	1
A8	M	60	Prostate	Adenocarcinoma	Malignant	n.a.	n.a.	1	1
A9	M	76	Prostate	Adenocarcinoma	Malignant	n.a.	n.a.	1	1
A10	M	69	Prostate	Adenocarcinoma (sparse)	Malignant	n.a.	n.a.	0	1
B1	M	71	Prostate	Adenocarcinoma	Malignant	1	1	1	1
B2	M	66	Prostate	Adenocarcinoma	Malignant	0	1	0	3
B3	M	63	Prostate	Adenocarcinoma	Malignant	1	2	1	1
B4	M	65	Prostate	Adenocarcinoma	Malignant	0	1	1	2
B5	M	72	Prostate	Adenocarcinoma	Malignant	1	1	0	3
B6	M	56	Prostate	Adenocarcinoma	Malignant	2	0	1	1
B7	M	78	Prostate	Adenocarcinoma	Malignant	1	1	1	3
B8	M	75	Prostate	Adenocarcinoma	Malignant	1	1	1	3
B9	M	60	Prostate	Adenocarcinoma	Malignant	1	n.a.	0	2
B10	M	73	Prostate	Adenocarcinoma (hyperplasia)	Malignant	n.a.	n.a.	2	2
C1	M	65	Prostate	Adenocarcinoma	Malignant	1	1	0	2
C2	M	61	Prostate	Adenocarcinoma	Malignant	1	1	1	2
C3	M	62	Prostate	Adenocarcinoma	Malignant	2	1	1	2
C4	M	69	Prostate	Adenocarcinoma	Malignant	1	2	1	1
C5	M	73	Prostate	Adenocarcinoma	Malignant	2	2	1	1
C6	M	72	Prostate	Adenocarcinoma (hyperplasia)	Malignant	1	1	1	2
C7	M	76	Prostate	Adenocarcinoma	Malignant	1	1	1	2
C8	M	64	Prostate	Adenocarcinoma	Malignant	1	1	1	2
C9	M	71	Prostate	Adenocarcinoma	Malignant	3	1	1	3
C10	M	60	Prostate	Adenocarcinoma	Malignant	1	1	2	1
D1	M	73	Prostate	Adenocarcinoma	Malignant	1	1	1	3
D2	M	71	Prostate	Adenocarcinoma	Malignant	1	0	1	1
D3	M	70	Prostate	Adenocarcinoma	Malignant	1	1	0	2
D4	M	64	Prostate	Adenocarcinoma	Malignant	1	0	1	1
D5	M	69	Prostate	Adenocarcinoma	Malignant	1	1	0	2

D6	M	73	Prostate	Adenocarcinoma (hyperplasia)	Malignant	1	1	2	2
D7	M	73	Prostate	Adenocarcinoma	Malignant	0	1	1	2
D8	M	64	Prostate	Adenocarcinoma	Malignant	0	0	1	3
D9	M	20	Prostate	Adenocarcinoma	Malignant	3	1	0	1
D10	M	66	Prostate	Adenocarcinoma	Malignant	2	1	1	1
E1	M	72	Prostate	Adenocarcinoma	Malignant	1	1	0	2
E2	M	58	Prostate	Adenocarcinoma	Malignant	1	1	3	2
E3	M	62	Prostate	Adenocarcinoma	Malignant	3	1	2	2
E4	M	70	Prostate	Adenocarcinoma	Malignant	1	1	1	3
E5	M	82	Prostate	Adenocarcinoma	Malignant	1	0	1	1
E6	M	51	Prostate	Adenocarcinoma	Malignant	1	1	1	2
E7	M	72	Prostate	Adenocarcinoma	Malignant	2	1	1	3
E8	M	64	Prostate	Adenocarcinoma	Malignant	1	1	1	1
E9	M	80	Prostate	Adenocarcinoma	Malignant	0	1	2	2
E10	M	65	Prostate	Adenocarcinoma	Malignant	2	0	2	1
F1	M	72	Prostate	Adenocarcinoma	Malignant	0	1	1	2
F2	M	73	Prostate	Adenocarcinoma	Malignant	1	1	0	1
F3	M	68	Prostate	Adenocarcinoma	Malignant	1	0	1	2
F4	M	79	Prostate	Adenocarcinoma (hyperplasia)	Malignant	3	0	2	0
F5	M	26	Prostate	Adenocarcinoma	Malignant	n.a.	0	0	1
F6	M	70	Prostate	Adenocarcinoma	Malignant	1	n.a.	n.a.	n.a.
F7	M	64	Prostate	Adenocarcinoma	Malignant	2	1	2	2
F8	M	64	Prostate	Adenocarcinoma	Malignant	0	1	3	2
F9	M	62	Prostate	Adenocarcinoma	Malignant	1	1	1	2
F10	M	75	Prostate	Adenocarcinoma	Malignant	1	1	1	2
G1	M	76	Prostate	Adenocarcinoma	Malignant	1	0	1	2
G2	M	78	Prostate	Adenocarcinoma	Malignant	1	1	2	1
G3	M	62	Prostate	Adenocarcinoma	Malignant	1	1	1	1
G4	M	67	Prostate	Adenocarcinoma	Malignant	1	0	2	1
G5	M	73	Prostate	Adenocarcinoma	Malignant	1	1	2	2
G6	M	82	Prostate	Adenocarcinoma	Malignant	1	0	2	2
G7	M	55	Prostate	Adenocarcinoma	Malignant	1	2	2	2
G8	M	40	Prostate	Adenocarcinoma (hyperplasia)	Malignant	2	1	3	3
G9	M	75	Prostate	Adenocarcinoma	Malignant	1	1	1	0
G10	M	75	Prostate	Adenocarcinoma	Malignant	1	1	1	1
H1	M	69	Prostate	Adenocarcinoma	Malignant	1	1	0	0
H2	M	75	Prostate	Adenocarcinoma	Malignant	3	1	1	0
H3	M	77	Prostate	Adenocarcinoma	Malignant	1	0	1	1
H4	M	75	Prostate	Adenocarcinoma	Malignant	0	1	1	2
H5	M	66	Prostate	Adenocarcinoma	Malignant	1	1	1	1
H6	M	87	Prostate	Adenocarcinoma	Malignant	0	1	1	1
H7	M	60	Prostate	Adenocarcinoma	Malignant	2	0	1	1
H8	M	81	Prostate	Transitional cell carcinoma	Malignant	2	1	2	2
H9	M	28	Prostate	Normal prostate tissue	Normal	1	n.a.	2	1
H10	M	37	Prostate	Normal prostate tissue	Normal	1	n.a.	2	1

*4. LC20813: **Lung cancer** tissue array*

position	sex	age	organ	pathology	type	TGFß1	TGFß2 (Acris)	TGFß2 (SC)	p-SMAD 2/3
A1	M	72	Lung	Squamous cell carcinoma	Malignant	1	n.a.	1	0
A3	M	55	Lung	Squamous cell carcinoma	Malignant	1	n.a.	1	0
A4	F	50	Lung	Squamous cell carcinoma	Malignant	n.a.	n.a.	1	0
A6	M	63	Lung	Squamous cell carcinoma (sparse)	Malignant	n.a.	n.a.	1	0
A7	M	73	Lung	Squamous cell carcinoma	Malignant	n.a.	n.a.	0	0
A8	M	53	Lung	Squamous cell carcinoma	Malignant	n.a.	n.a.	0	0
A9	M	69	Lung	Squamous cell carcinoma	Malignant	n.a.	n.a.	0	0
A10	M	66	Lung	Squamous cell carcinoma	Malignant	n.a.	n.a.	0	0
A11	M	61	Lung	Squamous cell carcinoma	Malignant	n.a.	n.a.	1	0
A12	M	64	Lung	Squamous cell carcinoma	Malignant	n.a.	n.a.	1	0
A13	M	48	Lung	Squamous cell carcinoma	Malignant	n.a.	n.a.	0	0
A14	M	59	Lung	Squamous cell carcinoma	Malignant	n.a.	n.a.	0	0
A15	M	51	Lung	Squamous cell carcinoma	Malignant	n.a.	n.a.	0	0
A16	F	70	Lung	Squamous cell carcinoma	Malignant	n.a.	n.a.	0	0
B1	M	63	Lung	Squamous cell carcinoma	Malignant	1	n.a.	2	2
B2	M	55	Lung	Squamous cell carcinoma	Malignant	2	n.a.	1	1
B3	M	76	Lung	Squamous cell carcinoma	Malignant	2	n.a.	2	1
B4	M	68	Lung	Squamous cell carcinoma	Malignant	2	n.a.	2	1
B5	M	45	Lung	Squamous cell carcinoma	Malignant	1	n.a.	2	1
B6	M	54	Lung	Squamous cell carcinoma	Malignant	3	n.a.	3	2
B7	M	50	Lung	Squamous cell carcinoma	Malignant	2	n.a.	2	1
B8	M	57	Lung	Squamous cell carcinoma	Malignant	n.a.	n.a.	2	2
B9	F	43	Lung	Squamous cell carcinoma	Malignant	n.a.	n.a.	1	2
B10	M	46	Lung	Squamous cell carcinoma	Malignant	n.a.	n.a.	3	2
B11	M	72	Lung	Squamous cell carcinoma	Malignant	n.a.	n.a.	1	1
B12	M	62	Lung	Squamous cell carcinoma	Malignant	n.a.	n.a.	1	2
B13	M	64	Lung	Squamous cell carcinoma with necrosis	Malignant	n.a.	n.a.	1	1
B14	M	61	Lung	Squamous cell carcinoma with necrosis	Malignant	n.a.	n.a.	1	1
B15	M	54	Lung	Squamous cell carcinoma	Malignant	n.a.	n.a.	0	0
B16	M	61	Lung	Squamous cell carcinoma	Malignant	n.a.	n.a.	0	0
C1	M	35	Lung	Squamous cell carcinoma	Malignant	1	n.a.	3	2
C2	M	53	Lung	Squamous cell carcinoma	Malignant	2	n.a.	3	3
C3	M	76	Lung	Squamous cell carcinoma (sparse) with necrosis	Malignant	1	n.a.	0	1
C4	M	53	Lung	Squamous cell carcinoma	Malignant	1	n.a.	1	2
C5	M	66	Lung	Squamous cell carcinoma	Malignant	2	n.a.	1	1
C6	M	76	Lung	Squamous cell carcinoma (lung tissue)	Malignant	1	n.a.	2	2
C7	M	70	Lung	Squamous cell carcinoma (tumoral necrosis)	Malignant	1	n.a.	0	0
C8	M	64	Lung	Squamous cell carcinoma	Malignant	1	n.a.	1	1
C9	M	68	Lung	Squamous cell carcinoma (bronchus, fibrous tissue and blood vessel)	Malignant	n.a.	n.a.	2	2
C10	M	25	Lung	Squamous cell carcinoma	Malignant	n.a.	n.a.	2	2
C11	M	66	Lung	Squamous cell carcinoma	Malignant	n.a.	n.a.	1	1
C12	M	66	Lung	Squamous cell carcinoma	Malignant	n.a.	n.a.	1	2
C13	M	53	Lung	Squamous cell carcinoma	Malignant	n.a.	n.a.	1	2
C14	M	39	Lung	Squamous cell carcinoma	Malignant	n.a.	n.a.	2	2
C15	M	60	Lung	Squamous cell carcinoma	Malignant	n.a.	n.a.	0	1
C16	M	59	Lung	Squamous cell carcinoma (cartilage, chronic inflammation of fibrous tissue and blood	Malignant	1	n.a.	0	0

				vessel)					
D1	M	62	Lung	Squamous cell carcinoma	Malignant	2	n.a.	1	2
D2	M	54	Lung	Squamous cell carcinoma	Malignant	2	n.a.	3	3
D3	M	67	Lung	Squamous cell carcinoma	Malignant	1	1	1	2
D4	M	54	Lung	Squamous cell carcinoma	Malignant	n.a.	1	2	1
D5	M	49	Lung	Squamous cell carcinoma	Malignant	1	n.a.	2	3
D6	M	46	Lung	Squamous cell carcinoma	Malignant	1	n.a.	1	2
D7	M	56	Lung	Squamous cell carcinoma	Malignant	1	n.a.	1	3
D8	F	55	Lung	Squamous cell carcinoma	Malignant	1	n.a.	2	2
D9	M	45	Lung	Squamous cell carcinoma	Malignant	1	n.a.	1	2
D10	M	47	Lung	Squamous cell carcinoma	Malignant	1	n.a.	1	2
D11	M	72	Lung	Squamous cell carcinoma	Malignant	1	n.a.	1	2
D12	M	66	Lung	Squamous cell carcinoma	Malignant	n.a.	n.a.	1	2
D13	M	62	Lung	Squamous cell carcinoma	Malignant	n.a.	n.a.	2	3
D14	M	48	Lung	Squamous cell carcinoma	Malignant	2	n.a.	1	3
D15	M	57	Lung	Squamous cell carcinoma	Malignant	1	n.a.	1	2
D16	M	70	Lung	Squamous cell carcinoma	Malignant	1	n.a.	0	1
E1	M	55	Lung	Squamous cell carcinoma	Malignant	1	1	1	3
E2	M	64	Lung	Squamous cell carcinoma	Malignant	1	2	2	2
E3	M	67	Lung	Squamous cell carcinoma	Malignant	n.a.	1	1	1
E4	M	75	Lung	Squamous cell carcinoma	Malignant	1	n.a.	n.a.	n.a.
E5	F	56	Lung	Squamous cell carcinoma	Malignant	3	n.a.	1	1
E6	M	77	Lung	Squamous cell carcinoma	Malignant	2	n.a.	2	1
E7	F	51	Lung	Squamous cell carcinoma	Malignant	1	1	2	2
E8	M	64	Lung	Squamous cell carcinoma (lung tissue)	Malignant	1	n.a.	2	1
E9	M	65	Lung	Squamous cell carcinoma with necrosis	Malignant	1	n.a.	1	1
E10	M	63	Lung	Squamous cell carcinoma	Malignant	1	n.a.	1	3
E11	M	52	Lung	Squamous cell carcinoma	Malignant	3	n.a.	3	3
E12	F	55	Lung	Squamous cell carcinoma	Malignant	1	n.a.	3	3
E13	M	71	Lung	Squamous cell carcinoma	Malignant	3	n.a.	3	3
E14	M	52	Lung	Squamous cell carcinoma	Malignant	1	n.a.	1	3
E15	F	63	Lung	Adenosquamous carcinoma	Malignant	1	n.a.	1	2
E16	F	67	Lung	Adenosquamous carcinoma	Malignant	2	n.a.	0	1
F1	F	46	Lung	Adenosquamous carcinoma	Malignant	2	2	1	2
F2	M	61	Lung	Adenosquamous carcinoma	Malignant	1	2	1	2
F3	M	54	Lung	Adenosquamous carcinoma	Malignant	n.a.	n.a.	n.a.	n.a.
F4	F	55	Lung	Adenosquamous carcinoma	Malignant	1	1	1	2
F5	M	68	Lung	Adenosquamous carcinoma	Malignant	3	1	2	1
F6	F	54	Lung	Papillary adenocarcinoma	Malignant	2	2	2	2
F7	M	61	Lung	Adenocarcinoma	Malignant	1	1	2	1
F8	F	60	Lung	Adenocarcinoma	Malignant	1	2	2	2
F9	F	62	Lung	Adenocarcinoma	Malignant	1	0	2	2
F10	M	38	Lung	Adenocarcinoma	Malignant	1	1	0	1
F11	F	42	Lung	Adenocarcinoma	Malignant	3	1	1	3
F12	F	56	Lung	Adenocarcinoma	Malignant	1	2	2	2
F13	M	59	Lung	Adenocarcinoma	Malignant	3	2	2	2
F14	M	33	Lung	Adenosquamous carcinoma	Malignant	1	0	2	3
F15	F	68	Lung	Adenocarcinoma	Malignant	1	0	1	2
F16	F	49	Lung	Adenocarcinoma with necrosis	Malignant	2	0	0	2
G1	F	56	Lung	Adenocarcinoma (chronic inflammation of fibrous tissue and blood vessel)	Malignant	2	1	1	1
G2	M	60	Lung	Papillary adenocarcinoma	Malignant	1	2	2	2

G3	M	39	Lung	Papillary adenocarcinoma	Malignant	2	1	1	2
G4	M	58	Lung	Papillary adenocarcinoma	Malignant	3	2	2	1
G5	F	56	Lung	Papillary adenocarcinoma	Malignant	1	1	2	2
G6	M	55	Lung	Papillary adenocarcinoma	Malignant	1	2	1	1
G7	M	62	Lung	Papillary adenocarcinoma	Malignant	1	2	1	2
G8	F	64	Lung	Papillary adenocarcinoma	Malignant	1	2	2	3
G9	M	72	Lung	Adenocarcinoma	Malignant	1	1	2	2
G10	F	53	Lung	Adenocarcinoma	Malignant	1	2	2	2
G11	M	65	Lung	Papillary adenocarcinoma	Malignant	1	2	2	2
G12	F	52	Lung	Papillary adenocarcinoma	Malignant	3	1	2	2
G13	F	47	Lung	Papillary adenocarcinoma	Malignant	1	3	1	2
G14	F	71	Lung	Papillary adenocarcinoma	Malignant	3	1	2	2
G15	M	49	Lung	Adenocarcinoma	Malignant	3	0	2	1
G16	M	58	Lung	Adenocarcinoma	Malignant	2	1	1	1
H1	M	62	Lung	Adenocarcinoma	Malignant	1	1	2	1
H2	F	54	Lung	Adenocarcinoma	Malignant	1	0	0	2
H3	M	38	Lung	Adenocarcinoma with necrosis	Malignant	2	0	0	1
H4	M	68	Lung	Adenocarcinoma	Malignant	1	1	2	1
H5	F	64	Lung	Adenocarcinoma	Malignant	2	3	2	2
H6	F	41	Lung	Adenocarcinoma	Malignant	n.a.	n.a.	n.a.	n.a.
H7	M	40	Lung	Adenocarcinoma	Malignant	2	3	1	2
H8	F	58	Lung	Adenocarcinoma with necrosis	Malignant	1	1	1	1
H9	M	64	Lung	Adenocarcinoma with necrosis	Malignant	1	1	1	1
H10	F	56	Lung	Papillary adenocarcinoma	Malignant	3	1	1	1
H11	F	57	Lung	Papillary adenocarcinoma	Malignant	1	3	2	2
H12	F	62	Lung	Adenocarcinoma with necrosis	Malignant	2	1	3	2
H13	M	60	Lung	Adenocarcinoma	Malignant	1	2	3	2
H14	F	64	Lung	Adenocarcinoma	Malignant	2	1	2	2
H15	M	82	Lung	Adenocarcinoma	Malignant	2	1	2	1
H16	M	60	Lung	Adenocarcinoma	Malignant	3	1	0	0
I1	F	50	Lung	Adenocarcinoma	Malignant	1	0	1	1
I2	M	46	Lung	Adenocarcinoma	Malignant	0	0	1	1
I3	M	69	Lung	Adenocarcinoma (sparse)	Malignant	n.a.	n.a.	n.a.	n.a.
I4	M	51	Lung	Adenocarcinoma	Malignant	1	3	2	2
I5	M	70	Lung	Adenocarcinoma	Malignant	2	1	2	2
I6	M	67	Lung	Adenocarcinoma	Malignant	1	2	2	2
I7	M	36	Lung	Adenocarcinoma	Malignant	2	2	2	2
I8	M	49	Lung	Adenocarcinoma	Malignant	3	0	2	2
I9	M	60	Lung	Adenocarcinoma	Malignant	1	1	2	2
I10	F	39	Lung	Adenocarcinoma	Malignant	2	1	1	1
I11	M	65	Lung	Adenocarcinoma	Malignant	3	1	2	1
I12	F	69	Lung	Adenocarcinoma	Malignant	2	1	2	2
I13	M	75	Lung	Adenocarcinoma	Malignant	1	1	2	3
I14	M	75	Lung	Adenocarcinoma	Malignant	1	1	2	2
I15	F	61	Lung	Adenocarcinoma	Malignant	2	1	2	2
I16	M	44	Lung	Adenocarcinoma	Malignant	1	1	0	1
J1	M	59	Lung	Adenocarcinoma	Malignant	1	n.a.	2	2
J2	F	68	Lung	Adenocarcinoma	Malignant	1	2	2	2
J3	M	65	Lung	Adenocarcinoma with necrosis	Malignant	1	1	1	1
J4	M	65	Lung	Adenosquamous carcinoma	Malignant	1	2	1	3
J5	M	39	Lung	Adenocarcinoma	Malignant	1	2	1	2

J6	M	74	Lung	Adenocarcinoma	Malignant	2	2	2	2
J7	M	50	Lung	Adenocarcinoma	Malignant	n.a.	n.a.	n.a.	n.a.
J8	F	36	Lung	Adenocarcinoma	Malignant	1	3	3	3
J9	M	46	Lung	Adenocarcinoma	Malignant	1	2	2	2
J10	M	69	Lung	Adenocarcinoma	Malignant	3	0	2	2
J11	M	30	Lung	Adenocarcinoma	Malignant	2	1	2	2
J12	M	65	Lung	Adenocarcinoma	Malignant	1	1	2	2
J13	M	52	Lung	Adenocarcinoma	Malignant	1	2	0	2
J14	M	66	Lung	Adenocarcinoma	Malignant	2	0	1	1
J15	M	47	Lung	Adenocarcinoma	Malignant	2	1	2	2
J16	F	52	Lung	Adenocarcinoma	Malignant	1	1	2	0
K1	F	58	Lung	Adenocarcinoma	Malignant	1	n.a.	0	1
K2	F	46	Lung	Adenocarcinoma	Malignant	1	1	1	1
K3	M	69	Lung	Adenocarcinoma	Malignant	1	1	2	2
K4	M	58	Lung	Small cell carcinoma	Malignant	0	0	0	2
K5	M	51	Lung	Small cell carcinoma	Malignant	2	1	2	1
K6	F	63	Lung	Small cell carcinoma	Malignant	0	2	0	0
K7	M	63	Lung	Small cell carcinoma	Malignant	1	0	3	3
K8	M	60	Lung	Small cell carcinoma	Malignant	1	0	0	2
K9	M	63	Lung	Small cell carcinoma	Malignant	1	1	1	1
K10	M	66	Lung	Small cell carcinoma	Malignant	1	2	0	1
K11	M	71	Lung	Small cell carcinoma	Malignant	1	1	1	2
K12	M	60	Lung	Small cell carcinoma	Malignant	1	1	1	2
K13	F	31	Lung	Small cell carcinoma	Malignant	2	1	1	1
K14	M	61	Lung	Small cell carcinoma	Malignant	2	n.a.	n.a.	n.a.
K15	F	52	Lung	Small cell carcinoma	Malignant	2	1	1	1
K16	F	69	Lung	Small cell carcinoma	Malignant	1	1	1	0
L1	F	43	Lung	Small cell carcinoma	Malignant	0	n.a.	0	1
L2	M	56	Lung	Small cell carcinoma	Malignant	1	1	0	2
L3	M	62	Lung	Small cell carcinoma	Malignant	1	1	1	3
L4	F	42	Lung	Small cell carcinoma	Malignant	n.a.	n.a.	n.a.	n.a.
L5	F	59	Lung	Small cell carcinoma (fibrous tissue and blood vessel)	Malignant	n.a.	0	1	1
L6	F	55	Lung	Small cell carcinoma	Malignant	1	1	1	2
L7	M	28	Lung	Small cell carcinoma	Malignant	1	1	0	2
L8	M	39	Lung	Small cell carcinoma	Malignant	n.a.	1	1	1
L9	M	61	Lung	Large cell carcinoma	Malignant	n.a.	1	2	2
L10	F	72	Lung	Large cell carcinoma	Malignant	n.a.	2	3	2
L11	M	46	Lung	Large cell carcinoma	Malignant	1	1	2	2
L12	F	64	Lung	Large cell carcinoma	Malignant	2	0	2	1
L13	F	55	Lung	Bronchioloalveolar carcinoma	Malignant	1	2	2	2
L14	M	52	Lung	Bronchioloalveolar carcinoma	Malignant	1	3	0	0
L15	M	55	Lung	Bronchioloalveolar carcinoma	Malignant	1	3	2	0
L16	F	64	Lung	Bronchioloalveolar carcinoma	Malignant	n.a.	1	2	0
M1	M	59	Lung	Mucinous bronchioloalveolar carcinoma	Malignant	n.a.	1	0	0
M2	F	50	Lung	Mucinous bronchioloalveolar carcinoma (sparse)	Malignant	n.a.	n.a.	n.a.	n.a.
M3	M	27	Lung	Mucinous bronchioloalveolar carcinoma	Malignant	n.a.	2	2	2
M4	M	48	Lung	Mucinous bronchioloalveolar carcinoma	Malignant	n.a.	n.a.	2	2
M5	M	56	Lung	Mucoepidermoid carcinoma	Malignant	n.a.	n.a.	2	1
M6	M	51	Lung	Mucoepidermoid carcinoma	Malignant	n.a.	n.a.	1	1
M7	M	48	Lung	Mucoepidermoid carcinoma	Malignant	n.a.	n.a.	1	1

M8	M	58	Lung	Mucoepidermoid carcinoma	Malignant	n.a.	n.a.	2	2
M9	M	49	Lung	Atypical carcinoid	Malignant	n.a.	n.a.	1	2
M10	M	47	Lung	Atypical carcinoid	Malignant	n.a.	1	1	2
M11	M	67	Lung	Atypical carcinoid	Malignant	n.a.	1	1	2
M12	M	65	Lung	Large cell neuroendocrine carcinoma	Malignant	n.a.	2	2	3
M13	M	43	Lung	Mixed large cell neuroendocrine carcinoma	Malignant	n.a.	n.a.	1	2
M14	M	58	Lung	Giant cell carcinoma	Malignant	n.a.	n.a.	1	1
M15	F	36	Lung	Basal cell carcinoma	Malignant	n.a.	n.a.	1	0
M16	M	66	Lung	Pleomorphic carcinoma	Malignant	n.a.	n.a.	1	0

*5. MS801: **Mesothelioma** tissue array with normal mesothelium*

position	sex	age	organ	pathology	type	TGFß1	TGFß2 (Acris)	TGFß2 (SC)	p-SMAD 2/3
A1	M	34	Pleura	Nonmalignant mesothelioma of left thoracic cavity	Benign	1	0	1	1
A2	M	34	Pleura	Nonmalignant mesothelioma of left thoracic cavity	Benign	1	0	1	1
A3	M	67	Bone	Malignant mesothelioma of right thoracic cavity	Malignant	1	1	1	1
A4	M	67	Bone	Malignant mesothelioma of right thoracic cavity	Malignant	1	1	2	2
A5	F	51	Blood vessel	Malignant mesothelioma of pulmonary artery	Malignant	0	1	2	1
A6	F	51	Blood vessel	Malignant mesothelioma of pulmonary artery	Malignant	0	1	2	0
A7	F	70	Abdominal cavity	Malignant mesothelioma	Malignant	2	2	2	0
A8	F	70	Abdominal cavity	Malignant mesothelioma	Malignant	1	2	2	1
A9	M	60	Abdominal cavity	Malignant mesothelioma	Malignant	1	2	1	0
A10	M	60	Abdominal cavity	Malignant mesothelioma	Malignant	1	2	0	0
B1	M	5	Abdominal cavity	Malignant mesothelioma	Malignant	1	1	1	3
B2	M	5	Abdominal cavity	Malignant mesothelioma	Malignant	1	1	2	3
B3	M	60	Abdominal cavity	Malignant mesothelioma	Malignant	3	3	2	3
B4	M	60	Abdominal cavity	Malignant mesothelioma with necrosis	Malignant	3	3	2	3
B5	F	47	Abdominal cavity	Malignant mesothelioma (fibrofatty tissue)	Malignant	2	3	3	2
B6	F	47	Abdominal cavity	Malignant mesothelioma (sparse)	Malignant	1	2	3	2
B7	M	33	Abdominal cavity	Malignant mesothelioma	Malignant	2	1	1	2
B8	M	33	Abdominal cavity	Malignant mesothelioma	Malignant	1	1	1	2
B9	F	38	Abdominal cavity	Malignant mesothelioma	Malignant	1	3	1	2
B10	F	38	Abdominal cavity	Malignant mesothelioma	Malignant	1	3	1	1
C1	F	71	Mesentery	Malignant mesothelioma	Malignant	1	1	1	3
C2	F	71	Mesentery	Malignant mesothelioma	Malignant	1	1	2	3
C3	F	29	Epiploon	Malignant mesothelioma	Malignant	2	3	2	2
C4	F	29	Epiploon	Malignant mesothelioma	Malignant	2	3	2	2
C5	M	63	Retroperitoneum	Malignant mesothelioma	Malignant	3	0	1	3
C6	M	63	Retroperitoneum	Malignant mesothelioma	Malignant	3	0	2	3
C7	M	48	Cardiac pericardium	Malignant mesothelioma	Malignant	2	0	3	3
C8	M	48	Cardiac pericardium	Malignant mesothelioma	Malignant	1	0	3	3
C9	M	43	Cardiac pericardium	Malignant mesothelioma	Malignant	1	2	3	2
C10	M	43	Cardiac pericardium	Malignant mesothelioma	Malignant	1	2	2	2
D1	F	32	Abdominal cavity	Malignant mesothelioma	Malignant	2	1	2	1
D2	F	32	Abdominal cavity	Malignant mesothelioma	Malignant	1	1	2	1

D3	F	48	Pleura	Malignant mesothelioma	Malignant	3	0	3	3
D4	F	48	Pleura	Malignant mesothelioma	Malignant	1	0	3	3
D5	F	23	Lung	Malignant mesothelioma	Malignant	1	1	0	2
D6	F	23	Lung	Malignant mesothelioma	Malignant	1	0	0	3
D7	F	18	Lung	Malignant mesothelioma	Malignant	1	1	1	3
D8	F	18	Lung	Malignant mesothelioma	Malignant	1	0	1	3
D9	F	56	Mediastinum	Malignant mesothelioma	Malignant	1	1	1	2
D10	F	56	Mediastinum	Malignant mesothelioma	Malignant	1	0	1	2
E1	F	58	Pleura	Malignant mesothelioma	Malignant	3	2	1	1
E2	F	58	Pleura	Malignant mesothelioma (sparse)	Malignant	2	1	1	2
E3	F	22	Pleura	Malignant mesothelioma	Malignant	1	1	3	1
E4	F	22	Pleura	Malignant mesothelioma	Malignant	1	1	3	1
E5	F	70	Pleura	Malignant mesothelioma	Malignant	2	1	3	2
E6	F	70	Pleura	Malignant mesothelioma	Malignant	2	2	2	2
E7	M	47	Pleura	Malignant mesothelioma	Malignant	2	1	2	2
E8	M	47	Pleura	Malignant mesothelioma	Malignant	1	1	1	2
E9	M	49	Pleura	Malignant mesothelioma	Malignant	2	1	2	2
E10	M	49	Pleura	Malignant mesothelioma	Malignant	3	1	1	1
F1	F	64	Pleura	Malignant mesothelioma	Malignant	1	3	2	1
F2	F	64	Pleura	Malignant mesothelioma	Malignant	1	3	2	1
F3	M	49	Pleura	Malignant mesothelioma	Malignant	2	1	3	3
F4	M	49	Pleura	Malignant mesothelioma	Malignant	2	1	3	3
F5	M	83	Pleura	Malignant mesothelioma	Malignant	2	1	1	1
F6	M	83	Pleura	Malignant mesothelioma	Malignant	2	1	1	1
F7	M	50	Cardiac pericardium	Malignant mesothelioma	Malignant	1	2	1	2
F8	M	50	Cardiac pericardium	Malignant mesothelioma	Malignant	1	2	1	2
F9	M	43	Cardiac pericardium	Malignant mesothelioma	Malignant	1	3	2	2
F10	M	43	Cardiac pericardium	Malignant mesothelioma	Malignant	1	3	1	1
G1	F	18	Pleura	Normal mesothelium tissue	Normal	3	1	1	0
G2	F	18	Pleura	Normal mesothelium tissue	Normal	3	1	1	1
G3	F	21	Epiploon	Normal mesothelium tissue	Normal	n.a.	0	1	1
G4	F	21	Epiploon	Normal mesothelium tissue	Normal	n.a.	0	1	2
G5	F	21	Epiploon	Normal mesothelium tissue	Normal	n.a.	n.a.	n.a.	n.a.
G6	F	21	Epiploon	Normal mesothelium tissue	Normal	n.a.	n.a.	n.a.	n.a.
G7	M	28	Epiploon	Normal mesothelium tissue (epiploon tissue)	Normal	n.a.	0	1	1
G8	M	28	Epiploon	Normal mesothelium tissue (epiploon tissue)	Normal	2	0	1	1
G9	M	48	Pleura	Normal mesothelium tissue	Normal	3	1	1	0
G10	M	48	Pleura	Normal mesothelium tissue	Normal	3	1	1	0
H1	F	18	Pleura	Normal mesothelium tissue (lung and pleura tissue)	Normal	3	1	0	0
H2	F	18	Pleura	Normal mesothelium tissue	Normal	3	1	1	1
H3	F	21	Pleura	Normal mesothelium tissue	Normal	0	1	1	1
H4	F	21	Pleura	Normal mesothelium tissue	Normal	1	1	1	1
H5	M	40	Lung	Normal pleura mesothelium (sparse) and lung tissue	Normal	3	2	1	1
H6	M	40	Lung	Normal pleura mesothelium (sparse) and lung tissue	Normal	3	1	n.a.	n.a.
H7	M	22	Lung	Normal mesothelium tissue (sparse)	Normal	n.a.	2	n.a.	n.a.
H8	M	22	Lung	Normal mesothelium tissue (lung tissue)	Normal	n.a.	2	n.a.	n.a.
H9	M	35	Lung	Normal pleura mesothelium (sparse) and lung tissue	Normal	3	1	n.a.	n.a.

H10	M	35	Lung	Normal pleura mesothelium (sparse) and lung tissue	Normal	3	1	n.a.	n.a.

6a. PA1921: **Mid-advanced stage pancreatic cancer** tissue array

**position**	**sex**	**age**	**organ**	**pathology**	**type**	**TGFß1**	**TGFß2 (Acris)**	**TGFß2 (SC)**	**p-SMAD 2/3**
A1	F	48	Pancreas	Duct adenocarcinoma (chronic inflammation of pancreas tissue)	Malignant	1	1	0	1
A2	F	41	Pancreas	Duct adenocarcinoma	Malignant	0	0	1	2
A3	M	57	Pancreas	Duct adenocarcinoma	Malignant	2	1	1	1
A4	F	42	Pancreas	Duct adenocarcinoma	Malignant	1	1	1	1
A5	F	47	Pancreas	Duct adenocarcinoma	Malignant	1	2	2	2
A6	F	54	Pancreas	Duct adenocarcinoma	Malignant	n.a	2	1	1
A7	F	40	Pancreas	Duct adenocarcinoma	Malignant	n.a	1	2	2
A8	F	54	Pancreas	Duct adenocarcinoma	Malignant	n.a	1	1	1
A9	F	48	Pancreas	Duct adenocarcinoma	Malignant	n.a	n.a.	1	1
A10	F	41	Pancreas	Duct adenocarcinoma (sparse)	Malignant	n.a	n.a.	0	1
A11	M	57	Pancreas	Duct adenocarcinoma	Malignant	n.a	n.a.	1	1
A12	F	42	Pancreas	Duct adenocarcinoma	Malignant	n.a	n.a.	1	1
A13	F	47	Pancreas	Duct adenocarcinoma (sparse)	Malignant	n.a	1	1	1
A14	F	54	Pancreas	Duct adenocarcinoma	Malignant	n.a	1	0	1
A15	F	40	Pancreas	Duct adenocarcinoma	Malignant	n.a	1	0	1
A16	F	54	Pancreas	Duct adenocarcinoma	Malignant	n.a	1	0	0
B1	F	51	Pancreas	Duct adenocarcinoma	Malignant	1	1	1	1
B2	M	54	Pancreas	Duct adenocarcinoma	Malignant	1	2	1	2
B3	M	60	Pancreas	Duct adenocarcinoma	Malignant	1	1	1	1
B4	M	47	Pancreas	Duct adenocarcinoma	Malignant	1	1	1	2
B5	M	39	Pancreas	Duct adenocarcinoma	Malignant	2	1	1	1
B6	M	54	Pancreas	Duct adenocarcinoma	Malignant	1	0	1	1
B7	F	62	Pancreas	Duct adenocarcinoma	Malignant	1	1	1	1
B8	F	64	Pancreas	Duct adenocarcinoma	Malignant	1	1	1	1
B9	F	51	Pancreas	Duct adenocarcinoma	Malignant	1	1	1	1
B10	M	54	Pancreas	Duct adenocarcinoma	Malignant	n.a	2	1	1
B11	M	60	Pancreas	Duct adenocarcinoma	Malignant	n.a	1	1	1
B12	M	47	Pancreas	Duct adenocarcinoma	Malignant	n.a	1	1	2
B13	M	39	Pancreas	Duct adenocarcinoma	Malignant	1	1	1	1
B14	M	54	Pancreas	Duct adenocarcinoma	Malignant	1	0	1	1
B15	F	62	Pancreas	Duct adenocarcinoma	Malignant	1	1	1	1
B16	F	64	Pancreas	Duct adenocarcinoma (sparse)	Malignant	1	1	0	0
C1	F	67	Pancreas	Duct adenocarcinoma	Malignant	1	2	1	1
C2	M	65	Pancreas	Duct adenocarcinoma	Malignant	1	2	1	1
C3	M	57	Pancreas	Duct adenocarcinoma	Malignant	3	1	1	2
C4	M	48	Pancreas	Duct adenocarcinoma	Malignant	0	3	2	2
C5	M	76	Pancreas	Duct adenocarcinoma	Malignant	2	3	3	3
C6	F	43	Pancreas	Duct adenocarcinoma (fibrofatty tissue)	Malignant	1	0	1	1
C7	M	57	Pancreas	Duct adenocarcinoma	Malignant	1	2	1	2
C8	M	49	Pancreas	Duct adenocarcinoma	Malignant	0	1	1	1
C9	F	67	Pancreas	Duct adenocarcinoma	Malignant	1	1	1	1
C10	M	65	Pancreas	Duct adenocarcinoma	Malignant	1	1	1	1
C11	M	57	Pancreas	Duct adenocarcinoma	Malignant	3	1	1	2

C12	M	48	Pancreas	Duct adenocarcinoma	Malignant	0	3	2	2
C13	M	76	Pancreas	Duct adenocarcinoma	Malignant	3	2	3	2
C14	F	43	Pancreas	Duct adenocarcinoma (chronic inflammation of fibrofatty tissue)	Malignant	1	0	1	1
C15	M	57	Pancreas	Duct adenocarcinoma	Malignant	1	2	1	1
C16	M	49	Pancreas	Duct adenocarcinoma	Malignant	1	1	0	1
D1	M	52	Pancreas	Duct adenocarcinoma	Malignant	1	2	1	2
D2	F	72	Pancreas	Duct adenocarcinoma	Malignant	2	2	2	2
D3	M	53	Pancreas	Duct adenocarcinoma	Malignant	1	1	0	2
D4	M	55	Pancreas	Duct adenocarcinoma	Malignant	1	2	2	2
D5	M	51	Pancreas	Duct adenocarcinoma	Malignant	1	1	1	2
D6	M	57	Pancreas	Duct adenocarcinoma (fibrofatty tissue)	Malignant	1	1	1	1
D7	M	49	Pancreas	Duct adenocarcinoma	Malignant	0	2	1	2
D8	M	64	Pancreas	Duct adenocarcinoma	Malignant	1	2	2	1
D9	M	52	Pancreas	Duct adenocarcinoma	Malignant	1	2	1	2
D10	F	72	Pancreas	Duct adenocarcinoma	Malignant	1	2	2	2
D11	M	53	Pancreas	Duct adenocarcinoma	Malignant	1	1	1	2
D12	M	55	Pancreas	Duct adenocarcinoma	Malignant	1	2	2	2
D13	M	51	Pancreas	Duct adenocarcinoma	Malignant	2	1	1	2
D14	M	57	Pancreas	Duct adenocarcinoma (chronic inflammation of fibrofatty tissue)	Malignant	1	2	1	2
D15	M	49	Pancreas	Duct adenocarcinoma	Malignant	1	2	1	2
D16	M	64	Pancreas	Duct adenocarcinoma	Malignant	2	2	1	1
E1	M	57	Pancreas	Duct adenocarcinoma	Malignant	1	1	1	0
E2	M	72	Pancreas	Duct adenocarcinoma (sparse)	Malignant	2	1	2	1
E3	M	42	Pancreas	Duct adenocarcinoma	Malignant	1	1	1	2
E4	M	55	Pancreas	Duct adenocarcinoma	Malignant	1	1	1	2
E5	M	47	Pancreas	Duct adenocarcinoma (pancreas duct tissue)	Malignant	3	1	2	2
E6	M	44	Pancreas	Duct adenocarcinoma	Malignant	1	0	2	2
E7	M	59	Pancreas	Duct adenocarcinoma	Malignant	1	2	3	2
E8	M	34	Pancreas	Duct adenocarcinoma	Malignant	0	1	2	1
E9	M	57	Pancreas	Duct adenocarcinoma	Malignant	1	2	1	2
E10	M	72	Pancreas	Duct adenocarcinoma	Malignant	3	2	2	2
E11	M	42	Pancreas	Duct adenocarcinoma	Malignant	1	1	1	2
E12	M	55	Pancreas	Duct adenocarcinoma	Malignant	1	2	1	2
E13	M	47	Pancreas	Duct adenocarcinoma	Malignant	1	1	3	2
E14	M	44	Pancreas	Duct adenocarcinoma	Malignant	2	0	2	2
E15	M	59	Pancreas	Duct adenocarcinoma (sparse)	Malignant	1	2	2	2
E16	M	34	Pancreas	Duct adenocarcinoma	Malignant	1	1	0	1
F1	M	61	Pancreas	Duct adenocarcinoma	Malignant	1	1	1	0
F2	F	39	Pancreas	Duct adenocarcinoma	Malignant	1	1	1	2
F3	M	44	Pancreas	Duct adenocarcinoma	Malignant	0	2	2	2
F4	M	59	Pancreas	Duct adenocarcinoma	Malignant	1	1	2	2
F5	F	67	Pancreas	Duct adenocarcinoma	Malignant	1	2	1	2
F6	F	72	Pancreas	Duct adenocarcinoma	Malignant	1	2	2	2
F7	F	41	Pancreas	Duct adenocarcinoma	Malignant	2	3	1	2
F8	M	51	Pancreas	Duct adenocarcinoma	Malignant	1	2	1	2
F9	M	61	Pancreas	Duct adenocarcinoma	Malignant	1	1	1	1
F10	F	39	Pancreas	Duct adenocarcinoma	Malignant	1	0	1	2
F11	M	44	Pancreas	Duct adenocarcinoma	Malignant	0	3	2	2
F12	M	59	Pancreas	Duct adenocarcinoma	Malignant	1	1	2	2
F13	F	67	Pancreas	Duct adenocarcinoma	Malignant	1	2	3	2

F14	F	72	Pancreas	Duct adenocarcinoma	Malignant	1	3	2	2
F15	F	41	Pancreas	Duct adenocarcinoma	Malignant	2	3	1	1
F16	M	51	Pancreas	Duct adenocarcinoma	Malignant	1	2	1	1
G1	M	41	Pancreas	Duct adenocarcinoma	Malignant	1	1	1	0
G2	F	58	Pancreas	Duct adenocarcinoma	Malignant	1	1	1	1
G3	M	60	Pancreas	Duct adenocarcinoma	Malignant	1	1	2	1
G4	M	41	Pancreas	Duct adenocarcinoma	Malignant	1	2	2	2
G5	F	68	Pancreas	Duct adenocarcinoma	Malignant	1	1	1	2
G6	M	52	Pancreas	Duct adenocarcinoma (sparse) with necrosis	Malignant	3	1	1	1
G7	F	51	Pancreas	Duct adenocarcinoma	Malignant	1	1	1	2
G8	F	76	Pancreas	Duct adenocarcinoma	Malignant	2	1	2	1
G9	M	41	Pancreas	Duct adenocarcinoma	Malignant	2	1	1	1
G10	F	58	Pancreas	Duct adenocarcinoma	Malignant	1	2	0	1
G11	M	60	Pancreas	Duct adenocarcinoma	Malignant	1	1	1	1
G12	M	41	Pancreas	Duct adenocarcinoma	Malignant	2	2	1	2
G13	F	68	Pancreas	Duct adenocarcinoma	Malignant	0	1	2	1
G14	M	52	Pancreas	Duct adenocarcinoma (sparse) with necrosis	Malignant	3	1	1	1
G15	F	51	Pancreas	Duct adenocarcinoma	Malignant	1	1	1	2
G16	F	76	Pancreas	Duct adenocarcinoma	Malignant	1	1	1	1
H1	F	62	Pancreas	Adenocarcinoma	Malignant	1	1	1	1
H2	M	51	Pancreas	Adenocarcinoma	Malignant	2	0	1	2
H3	F	60	Pancreas	Duct adenocarcinoma	Malignant	1	1	1	1
H4	M	76	Pancreas	Duct adenocarcinoma	Malignant	2	2	1	2
H5	M	78	Pancreas	Duct adenocarcinoma	Malignant	1	1	1	1
H6	F	53	Pancreas	Duct adenocarcinoma	Malignant	1	2	1	2
H7	F	48	Pancreas	Duct adenocarcinoma	Malignant	3	1	1	2
H8	M	55	Pancreas	Duct adenocarcinoma	Malignant	1	2	2	2
H9	F	62	Pancreas	Adenocarcinoma	Malignant	0	2	2	3
H10	M	51	Pancreas	Adenocarcinoma	Malignant	2	0	1	2
H11	F	60	Pancreas	Duct adenocarcinoma	Malignant	0	1	1	1
H12	M	76	Pancreas	Duct adenocarcinoma (fibrous tissue and blood vessel)	Malignant	3	1	1	2
H13	M	78	Pancreas	Duct adenocarcinoma	Malignant	0	2	2	2
H14	F	53	Pancreas	Duct adenocarcinoma	Malignant	2	1	1	2
H15	F	48	Pancreas	Duct adenocarcinoma	Malignant	1	2	2	1
H16	M	55	Pancreas	Duct adenocarcinoma	Malignant	1	2	1	1
I1	M	59	Pancreas	Duct adenocarcinoma	Malignant	1	1	1	1
I2	M	62	Pancreas	Duct adenocarcinoma	Malignant	1	1	2	2
I3	M	67	Pancreas	Adenocarcinoma (sparse)	Malignant	1	0	3	3
I4	F	66	Pancreas	Duct adenocarcinoma	Malignant	1	2	1	2
I5	M	49	Pancreas	Adenosquamous carcinoma (fibrous tissue and blood vessel)	Malignant	2	0	1	1
I6	M	50	Pancreas	Squamous cell carcinoma	Malignant	1	1	1	3
I7	M	73	Pancreas	Undifferentiated carcinoma	Malignant	3	1	2	2
I8	M	65	Pancreas	Undifferentiated carcinoma	Malignant	2	0	1	1
I9	M	59	Pancreas	Duct adenocarcinoma	Malignant	2	2	2	2
I10	M	62	Pancreas	Duct adenocarcinoma (sparse)	Malignant	1	1	1	2
I11	M	67	Pancreas	Adenocarcinoma	Malignant	1	0	3	3
I12	F	66	Pancreas	Duct adenocarcinoma	Malignant	1	2	1	2
I13	M	49	Pancreas	Adenosquamous carcinoma	Malignant	1	1	2	1
I14	M	50	Pancreas	Squamous cell carcinoma	Malignant	1	1	1	2
I15	M	73	Pancreas	Undifferentiated carcinoma	Malignant	1	1	1	1

I16	M	65	Pancreas	Undifferentiated carcinoma	Malignant	2	0	1	0
J1	F	56	Pancreas	Undifferentiated carcinoma	Malignant	1	0	1	1
J2	F	52	Pancreas	Carcinoid	Malignant	0	0	0	3
J3	M	51	Pancreas	Atypical carcinoid	Malignant	1	0	1	1
J4	M	42	Pancreas	Neuroendocrine carcinoma	Malignant	1	0	3	2
J5	F	52	Pancreas	Adenocarcinoma	Malignant	2	2	2	2
J6	F	45	Pancreas	Adenocarcinoma	Malignant	2	1	2	3
J7	M	49	Pancreas	Adenocarcinoma	Malignant	1	2	2	2
J8	M	65	Pancreas	Adenocarcinoma	Malignant	0	0	0	3
J9	F	56	Pancreas	Undifferentiated carcinoma	Malignant	1	1	1	2
J10	F	52	Pancreas	Carcinoid	Malignant	1	0	1	3
J11	M	51	Pancreas	Atypical carcinoid	Malignant	1	1	1	2
J12	M	42	Pancreas	Neuroendocrine carcinoma	Malignant	1	1	2	1
J13	F	52	Pancreas	Adenocarcinoma (chronic inflammation of pancreas tissue)	Malignant	2	1	2	2
J14	F	45	Pancreas	Adenocarcinoma	Malignant	2	1	2	3
J15	M	49	Pancreas	Adenocarcinoma	Malignant	1	2	2	2
J16	M	65	Pancreas	Adenocarcinoma	Malignant	0	1	1	2
K1	M	52	Pancreas	Adenocarcinoma	Malignant	1	0	1	1
K2	F	56	Pancreas	Adenocarcinoma	Malignant	1	1	1	2
K3	F	76	Pancreas	Adenocarcinoma	Malignant	1	1	1	2
K4	M	45	Pancreas	Adenocarcinoma	Malignant	2	1	2	2
K5	M	41	Pancreas	Adenocarcinoma	Malignant	1	1	2	1
K6	M	62	Pancreas	Adenocarcinoma	Malignant	1	0	2	2
K7	M	50	Pancreas	Adenocarcinoma	Malignant	1	0	1	2
K8	M	60	Pancreas	Adenocarcinoma	Malignant	2	2	3	3
K9	M	52	Pancreas	Adenocarcinoma	Malignant	1	2	1	1
K10	F	56	Pancreas	Adenocarcinoma	Malignant	1	1	1	2
K11	F	76	Pancreas	Adenocarcinoma (sparse) of liver	Malignant	1	2	2	2
K12	M	45	Pancreas	Adenocarcinoma	Malignant	2	2	1	1
K13	M	41	Pancreas	Adenocarcinoma	Malignant	1	1	2	1
K14	M	62	Pancreas	Adenocarcinoma	Malignant	1	0	2	2
K15	M	50	Pancreas	Adenocarcinoma	Malignant	1	1	1	1
K16	M	60	Pancreas	Adenocarcinoma	Malignant	0	3	1	2
L1	M	56	Pancreas	Duct adenocarcinoma	Malignant	2	0	0	0
L2	F	49	Pancreas	Adenosquamous carcinoma	Malignant	0	1	0	1
L3	M	25	Pancreas	Adenocarcinoma	Malignant	2	1	1	1
L4	M	38	Pancreas	Adenocarcinoma	Malignant	0	1	1	1
L5	M	42	Pancreas	Duct adenocarcinoma	Malignant	n.a	0	2	1
L6	M	59	Pancreas	Adenocarcinoma (pancreas tissue)	Malignant	n.a	1	2	1
L7	F	60	Pancreas	Duct adenocarcinoma	Malignant	n.a	1	0	0
L8	F	13	Pancreas	Solid pseudopapillary carcinoma	Malignant	n.a	1	1	1
L9	M	56	Pancreas	Duct adenocarcinoma	Malignant	n.a	1	0	1
L10	F	49	Pancreas	Adenosquamous carcinoma	Malignant	n.a	1	0	1
L11	M	25	Pancreas	Adenocarcinoma (sparse)	Malignant	n.a	2	2	1
L12	M	38	Pancreas	Adenocarcinoma	Malignant	n.a	1	1	1
L13	M	42	Pancreas	Duct adenocarcinoma	Malignant	n.a	1	1	1
L14	M	59	Pancreas	Adenocarcinoma	Malignant	n.a	1	2	2
L15	F	60	Pancreas	Duct adenocarcinoma	Malignant	n.a	1	1	1
L16	F	13	Pancreas	Solid pseudopapillary carcinoma	Malignant	n.a	1	1	0

*6b. PA2081a: **Pancreatic disease** spectrum tissue array*

Position	sex	age	organ	pathology	type	TGFß1	TGFß2 (Acris)	TGFß2 (SC)	p-SMAD 2/3
A1	M	60	Pancreas	Duct adenocarcinoma (chronic inflammation of fibrofatty tissue and blood vessel)	Malignant	1	0	1	1
A2	M	60	Pancreas	Duct adenocarcinoma (chronic pancreatitis)	Malignant	2	1	1	1
A3	F	72	Pancreas	Duct adenocarcinoma	Malignant	1	1	1	2
A4	F	72	Pancreas	Duct adenocarcinoma	Malignant	0	1	1	2
A5	M	57	Pancreas	Duct adenocarcinoma (pancreatic tissue)	Malignant	1	1	1	2
A6	M	57	Pancreas	Duct adenocarcinoma	Malignant	1	1	1	1
A7	M	59	Pancreas	Duct adenocarcinoma (tumoral necrosis)	Malignant	1	0	0	0
A8	M	59	Pancreas	Duct adenocarcinoma (tumoral necrosis)	Malignant	1	0	0	0
A9	M	65	Pancreas	Duct adenocarcinoma	Malignant	1	1	1	1
A10	M	65	Pancreas	Duct adenocarcinoma	Malignant	1	1	0	0
A11	F	47	Pancreas	Duct adenocarcinoma	Malignant	1	1	1	1
A12	F	47	Pancreas	Duct adenocarcinoma	Malignant	1	1	0	0
A13	M	34	Pancreas	Duct adenocarcinoma	Malignant	1	1	0	0
A14	M	34	Pancreas	Duct adenocarcinoma	Malignant	1	1	0	0
A15	M	42	Pancreas	Duct adenocarcinoma	Malignant	1	2	0	0
A16	M	42	Pancreas	Duct adenocarcinoma	Malignant	0	1	0	0
B1	F	58	Pancreas	Duct adenocarcinoma	Malignant	1	1	1	2
B2	F	58	Pancreas	Duct adenocarcinoma	Malignant	2	1	1	2
B3	M	55	Pancreas	Duct adenocarcinoma	Malignant	1	1	2	1
B4	M	55	Pancreas	Duct adenocarcinoma	Malignant	1	2	1	2
B5	M	53	Pancreas	Duct adenocarcinoma	Malignant	1	2	2	3
B6	M	53	Pancreas	Duct adenocarcinoma	Malignant	1	2	2	3
B7	M	55	Pancreas	Duct adenocarcinoma	Malignant	0	0	1	3
B8	M	55	Pancreas	Duct adenocarcinoma	Malignant	1	0	1	2
B9	F	55	Pancreas	Duct adenocarcinoma	Malignant	1	0	0	1
B10	F	55	Pancreas	Duct adenocarcinoma	Malignant	0	0	0	1
B11	M	64	Pancreas	Duct adenocarcinoma	Malignant	0	2	0	2
B12	M	64	Pancreas	Duct adenocarcinoma	Malignant	0	1	0	2
B13	F	72	Pancreas	Duct adenocarcinoma	Malignant	1	1	1	1
B14	F	72	Pancreas	Duct adenocarcinoma (sparse)	Malignant	2	1	1	1
B15	F	44	Pancreas	Duct adenocarcinoma	Malignant	3	1	0	0
B16	F	44	Pancreas	Duct adenocarcinoma	Malignant	3	1	0	0
C1	M	55	Pancreas	Duct adenocarcinoma (fibrofatty tissue and blood vessel)	Malignant	1	0	1	1
C2	M	55	Pancreas	Duct adenocarcinoma (fibrofatty tissue and blood vessel)	Malignant	1	0	1	1
C3	M	74	Pancreas	Duct adenocarcinoma	Malignant	3	1	1	1
C4	M	74	Pancreas	Duct adenocarcinoma	Malignant	2	1	1	1
C5	M	61	Pancreas	Duct adenocarcinoma	Malignant	0	2	2	2
C6	M	61	Pancreas	Duct adenocarcinoma	Malignant	1	2	2	2
C7	M	50	Pancreas	Duct adenocarcinoma (sparse)	Malignant	1	0	0	1
C8	M	50	Pancreas	Duct adenocarcinoma (sparse)	Malignant	0	1	0	1
C9	M	62	Pancreas	Duct adenocarcinoma	Malignant	1	1	1	2
C10	M	62	Pancreas	Duct adenocarcinoma	Malignant	1	1	1	2
C11	F	62	Pancreas	Duct adenocarcinoma	Malignant	1	1	1	2
C12	F	62	Pancreas	Duct adenocarcinoma	Malignant	1	1	1	2

C13	F	54	Pancreas	Duct adenocarcinoma	Malignant	0	2	1	1
C14	F	54	Pancreas	Duct adenocarcinoma	Malignant	0	2	1	1
C15	M	41	Pancreas	Duct adenocarcinoma	Malignant	2	2	2	1
C16	M	41	Pancreas	Duct adenocarcinoma	Malignant	2	1	1	0
D1	M	52	Pancreas	Duct adenocarcinoma	Malignant	0	1	1	1
D2	M	52	Pancreas	Duct adenocarcinoma	Malignant	0	1	1	1
D3	F	64	Pancreas	Duct adenocarcinoma	Malignant	1	1	1	1
D4	F	64	Pancreas	Duct adenocarcinoma	Malignant	0	1	1	1
D5	M	45	Pancreas	Duct adenocarcinoma	Malignant	1	3	2	2
D6	M	45	Pancreas	Duct adenocarcinoma	Malignant	1	2	2	2
D7	F	43	Pancreas	Duct adenocarcinoma	Malignant	1	1	3	2
D8	F	43	Pancreas	Duct adenocarcinoma	Malignant	1	1	3	2
D9	F	51	Pancreas	Duct adenocarcinoma	Malignant	1	1	2	3
D10	F	51	Pancreas	Duct adenocarcinoma	Malignant	1	1	2	3
D11	M	60	Pancreas	Duct adenocarcinoma (pancreatic tissue)	Malignant	1	1	1	3
D12	M	60	Pancreas	Duct adenocarcinoma (pancreatic tissue)	Malignant	1	1	0	2
D13	M	64	Pancreas	Duct adenocarcinoma	Malignant	0	1	1	2
D14	M	64	Pancreas	Duct adenocarcinoma	Malignant	0	1	1	2
D15	M	67	Pancreas	Duct adenocarcinoma	Malignant	1	0	1	1
D16	M	67	Pancreas	Duct adenocarcinoma	Malignant	1	1	1	0
E1	F	40	Pancreas	Duct adenocarcinoma	Malignant	1	1	1	1
E2	F	40	Pancreas	Duct adenocarcinoma	Malignant	1	2	2	2
E3	F	55	Pancreas	Duct adenocarcinoma with necrosis (sparse)	Malignant	2	0	1	1
E4	F	55	Pancreas	Duct adenocarcinoma with necrosis (sparse)	Malignant	2	0	0	1
E5	F	63	Pancreas	Duct adenocarcinoma	Malignant	2	1	1	1
E6	F	63	Pancreas	Duct adenocarcinoma	Malignant	2	2	1	2
E7	M	67	Pancreas	Duct adenocarcinoma (sparse)	Malignant	2	0	1	3
E8	M	67	Pancreas	Duct adenocarcinoma	Malignant	3	0	1	3
E9	F	62	Pancreas	Duct adenocarcinoma	Malignant	0	2	2	2
E10	F	62	Pancreas	Duct adenocarcinoma	Malignant	0	2	2	2
E11	M	78	Pancreas	Duct adenocarcinoma	Malignant	1	1	1	2
E12	M	78	Pancreas	Duct adenocarcinoma	Malignant	1	1	2	2
E13	M	65	Pancreas	Duct adenocarcinoma	Malignant	2	1	1	2
E14	M	65	Pancreas	Duct adenocarcinoma	Malignant	2	0	1	2
E15	M	53	Pancreas	Duct adenocarcinoma	Malignant	1	1	1	2
E16	M	53	Pancreas	Duct adenocarcinoma	Malignant	1	2	1	1
F1	F	39	Pancreas	Duct adenocarcinoma	Malignant	0	1	1	2
F2	F	39	Pancreas	Duct adenocarcinoma	Malignant	1	1	1	1
F3	M	60	Pancreas	Duct adenocarcinoma	Malignant	1	1	1	1
F4	M	60	Pancreas	Duct adenocarcinoma	Malignant	1	1	2	2
F5	F	49	Pancreas	Adenosquamous carcinoma	Malignant	1	1	1	1
F6	F	49	Pancreas	Adenosquamous carcinoma	Malignant	1	1	1	1
F7	M	50	Pancreas	Adenosquamous carcinoma	Malignant	1	1	2	2
F8	M	50	Pancreas	Adenosquamous carcinoma	Malignant	1	1	2	2
F9	M	49	Pancreas	Adenosquamous carcinoma	Malignant	1	1	2	1
F10	M	49	Pancreas	Adenosquamous carcinoma	Malignant	2	1	1	1
F11	F	49	Abdominal cavity	Islet cell carcinoma	Malignant	0	0	3	3
F12	F	49	Abdominal cavity	Islet cell carcinoma	Malignant	0	0	3	3
F13	M	66	Epiploon	Metastatic duct adenocarcinoma from	Metastasis	0	2	1	2

				pancreas					
F14	M	66	Epiploon	Metastatic duct adenocarcinoma from pancreas	Metastasis	1	2	1	2
F15	F	51	Abdominal cavity	Metastatic duct adenocarcinoma from pancreas	Metastasis	2	1	2	2
F16	F	51	Abdominal cavity	Metastatic duct adenocarcinoma from pancreas	Metastasis	2	1	2	1
G1	M	60	Liver	Metastatic duct adenocarcinoma from pancreas	Metastasis	1	1	2	2
G2	M	60	Liver	Metastatic duct adenocarcinoma from pancreas	Metastasis	1	1	1	1
G3	M	53	Liver	Metastatic duct adenocarcinoma from pancreas	Metastasis	0	3	1	2
G4	M	53	Liver	Metastatic duct adenocarcinoma from pancreas	Metastasis	1	3	1	3
G5	M	59	Liver	Metastatic duct adenocarcinoma from pancreas	Metastasis	1	0	2	3
G6	M	59	Liver	Metastatic duct adenocarcinoma from pancreas	Metastasis	1	0	1	3
G7	M	62	Lymph node	Metastatic duct adenocarcinoma from pancreas	Metastasis	1	0	2	3
G8	M	62	Lymph node	Metastatic duct adenocarcinoma from pancreas	Metastasis	1	0	2	3
G9	F	60	Pancreas	Islet cell tumor	Benign	2	1	1	1
G10	F	60	Pancreas	Islet cell tumor	Benign	2	1	1	2
G11	M	64	Pancreas	Islet cell tumor	Benign	2	0	3	3
G12	M	64	Pancreas	Islet cell tumor	Benign	2	0	3	3
G13	F	47	Pancreas	Islet cell tumor	Benign	3	0	1	0
G14	F	47	Pancreas	Islet cell tumor	Benign	3	0	1	1
G15	F	37	Pancreas	Islet cell tumor	Benign	1	1	1	2
G16	F	37	Pancreas	Islet cell tumor	Benign	1	1	1	1
H1	M	77	Pancreas	Islet cell tumor	Benign	1	1	3	2
H2	M	77	Pancreas	Islet cell tumor	Benign	1	1	3	2
H3	F	35	Pancreas	Islet cell tumor	Benign	0	0	0	1
H4	F	35	Pancreas	Islet cell tumor	Benign	0	1	1	1
H5	M	40	Pancreas	Islet cell tumor	Benign	2	0	3	1
H6	M	40	Pancreas	Islet cell tumor	Benign	2	0	3	1
H7	F	48	Pancreas	Islet cell tumor (pancreatic tissue)	Benign	2	1	1	1
H8	F	48	Pancreas	Islet cell tumor (pancreatic tissue)	Benign	2	1	1	1
H9	F	59	Pancreas	Islet cell tumor	Benign	1	1	2	2
H10	F	59	Pancreas	Islet cell tumor	Benign	1	0	2	2
H11	F	23	Pancreas	Islet cell tumor	Benign	3	0	1	1
H12	F	23	Pancreas	Islet cell tumor	Benign	3	0	1	1
H13	M	39	Pancreas	Chronic pancreatitis	Inflammation	1	2	1	3
H14	M	39	Pancreas	Chronic pancreatitis	Inflammation	1	1	1	2
H15	F	49	Pancreas	Chronic pancreatitis	Inflammation	3	1	2	2
H16	F	49	Pancreas	Chronic pancreatitis	Inflammation	3	0	2	1
I1	F	65	Pancreas	Mild chronic inflammation	Inflammation	2	0	1	2
I2	F	65	Pancreas	Mild chronic inflammation	Inflammation	1	0	1	2
I3	M	68	Pancreas	Chronic pancreatitis	Inflammation	2	1	2	3
I4	M	68	Pancreas	Chronic pancreatitis	Inflammation	3	1	2	2
I5	M	65	Pancreas	Chronic inflammation (chronic inflammation of fibrous tissue and blood vessel)	Inflammation	3	1	1	1
I6	M	65	Pancreas	Chronic pancreatitis	Inflammation	1	2	2	2
I7	M	67	Pancreas	Chronic pancreatitis	Inflammation	1	2	2	1

I8	M	67	Pancreas	Chronic pancreatitis	Inflammation	1	2	2	1
I9	M	66	Pancreas	Mild chronic inflammation	Inflammation	2	2	2	2
I10	M	66	Pancreas	Mild chronic inflammation	Inflammation	2	2	2	2
I11	F	33	Pancreas	Chronic pancreatitis	Inflammation	3	1	2	2
I12	F	33	Pancreas	Chronic pancreatitis	Inflammation	2	2	2	2
I13	M	62	Pancreas	Chronic pancreatitis	Inflammation	3	0	2	1
I14	M	62	Pancreas	Chronic pancreatitis	Inflammation	3	0	2	1
I15	F	50	Pancreas	Mild chronic inflammation	Inflammation	2	1	1	1
I16	F	50	Pancreas	Mild chronic inflammation	Inflammation	2	2	1	1
J1	F	55	Pancreas	Mild chronic inflammation	Inflammation	2	1	1	1
J2	F	55	Pancreas	Mild chronic inflammation	Inflammation	2	1	1	1
J3	M	53	Pancreas	Cancer adjacent normal pancreatic tissue	NAT	1	2	1	2
J4	M	53	Pancreas	Cancer adjacent normal pancreatic tissue	NAT	1	2	1	2
J5	M	62	Pancreas	Cancer adjacent normal pancreatic tissue	NAT	3	1	2	1
J6	M	62	Pancreas	Cancer adjacent normal pancreatic tissue	NAT	3	1	2	2
J7	M	76	Pancreas	Cancer adjacent normal pancreatic tissue	NAT	3	1	2	1
J8	M	76	Pancreas	Cancer adjacent normal pancreatic tissue	NAT	3	1	1	1
J9	M	72	Pancreas	Cancer adjacent normal pancreatic tissue	NAT	2	1	1	1
J10	M	72	Pancreas	Cancer adjacent normal pancreatic tissue	NAT	2	1	1	1
J11	F	75	Pancreas	Cancer adjacent normal pancreatic tissue	NAT	3	1	1	1
J12	F	75	Pancreas	Cancer adjacent normal pancreatic tissue	NAT	3	1	1	1
J13	M	54	Pancreas	Cancer adjacent normal pancreatic tissue	NAT	3	1	1	1
J14	M	54	Pancreas	Cancer adjacent normal pancreatic tissue	NAT	2	0	0	1
J15	M	65	Pancreas	Cancer adjacent normal pancreatic tissue (sparse)	NAT	2	1	1	1
J16	M	65	Pancreas	Cancer adjacent normal pancreatic tissue	NAT	1	1	1	1
K1	M	60	Pancreas	Cancer adjacent normal pancreatic tissue	NAT	2	1	1	2
K2	M	60	Pancreas	Cancer adjacent normal pancreatic tissue	NAT	1	1	1	2
K3	F	69	Pancreas	Cancer adjacent normal pancreatic tissue	NAT	1	1	1	2
K4	F	69	Pancreas	Cancer adjacent normal pancreatic tissue	NAT	1	1	1	2
K5	F	53	Pancreas	Cancer adjacent normal pancreatic tissue with ductal hyperplasia (sparse)	NAT	3	1	2	1
K6	F	53	Pancreas	Cancer adjacent normal pancreatic tissue	NAT	3	1	1	1
K7	F	69	Pancreas	Cancer adjacent normal pancreatic tissue	NAT	3	1	1	1
K8	F	69	Pancreas	Cancer adjacent normal pancreatic tissue	NAT	3	1	1	1
K9	M	64	Pancreas	Cancer adjacent normal pancreatic tissue	NAT	3	1	1	1
K10	M	64	Pancreas	Cancer adjacent normal pancreatic tissue	NAT	3	1	1	1
K11	M	47	Pancreas	Cancer adjacent normal pancreatic tissue	NAT	1	2	1	2
K12	M	47	Pancreas	Cancer adjacent normal pancreatic tissue	NAT	1	2	1	2
K13	M	47	Pancreas	Cancer adjacent normal pancreatic tissue	NAT	3	2	1	1
K14	M	47	Pancreas	Cancer adjacent normal pancreatic tissue	NAT	2	1	1	2
K15	F	56	Pancreas	Cancer adjacent normal pancreatic tissue	NAT	3	1	1	1
K16	F	56	Pancreas	Cancer adjacent normal pancreatic tissue	NAT	3	1	1	0
L1	M	50	Pancreas	Cancer adjacent normal pancreatic tissue	NAT	1	2	1	1
L2	M	50	Pancreas	Cancer adjacent normal pancreatic tissue	NAT	1	2	1	2
L3	M	67	Pancreas	Cancer adjacent normal pancreatic tissue	NAT	3	1	2	2
L4	M	67	Pancreas	Cancer adjacent normal pancreatic tissue	NAT	2	1	2	3
L5	M	48	Pancreas	Cancer adjacent normal pancreatic tissue	NAT	2	1	1	1
L6	M	48	Pancreas	Cancer adjacent normal pancreatic tissue	NAT	3	1	1	1
L7	M	48	Pancreas	Cancer adjacent normal pancreatic tissue	NAT	3	0	1	1
L8	M	48	Pancreas	Cancer adjacent normal pancreatic tissue	NAT	3	0	1	1
L9	M	60	Pancreas	Cancer adjacent normal pancreatic tissue	NAT	2	1	1	2

L10	M	60	Pancreas	Cancer adjacent normal pancreatic tissue	NAT	2	2	2	2
L11	M	69	Pancreas	Cancer adjacent normal pancreatic tissue	NAT	1	1	1	1
L12	M	69	Pancreas	Cancer adjacent normal pancreatic tissue	NAT	2	2	2	1
L13	M	35	Pancreas	Normal pancreatic tissue	Normal	1	1	1	1
L14	M	35	Pancreas	Normal pancreatic tissue	Normal	1	1	1	1
L15	M	50	Pancreas	Normal pancreatic tissue	Normal	3	1	1	1
L16	M	50	Pancreas	Normal pancreatic tissue	Normal	1	1	0	0
M1	F	35	Pancreas	Normal pancreatic tissue	Normal	1	1	1	1
M2	F	35	Pancreas	Normal pancreatic tissue	Normal	1	1	1	2
M3	M	30	Pancreas	Normal pancreatic tissue	Normal	1	1	2	1
M4	M	30	Pancreas	Normal pancreatic tissue	Normal	1	1	1	1
M5	M	30	Pancreas	Normal pancreatic tissue (small intestine tissue)	Normal	1	1	1	1
M6	M	30	Pancreas	Normal pancreatic tissue (chronic inflammation of fibrous tissue and blood vessel)	Normal	1	1	1	1
M7	M	38	Pancreas	Normal pancreatic tissue	Normal	2	1	1	1
M8	M	38	Pancreas	Normal pancreatic tissue	Normal	1	1	1	1
M9	M	40	Pancreas	Normal pancreatic tissue	Normal	1	1	1	1
M10	M	40	Pancreas	Normal pancreatic tissue	Normal	1	1	1	1
M11	M	35	Pancreas	Normal pancreatic tissue	Normal	1	2	1	2
M12	M	35	Pancreas	Normal pancreatic tissue	Normal	1	2	1	2
M13	M	25	Pancreas	Normal pancreatic tissue	Normal	1	2	0	1
M14	M	25	Pancreas	Normal pancreatic tissue	Normal	0	2	1	0
M15	M	47	Pancreas	Normal pancreatic tissue	Normal	1	1	1	0
M16	M	47	Pancreas	Normal pancreatic tissue	Normal	1	1	1	0

7. BC03119: ***Liver*** *carcinoma and normal tissue*

position	sex	age	organ	pathology	type	TGFß1	TGFß2 (Acris)	TGFß2 (SC)	p-SMAD 2/3
A1	F	40	Liver	Cholangiocellular carcinoma	Malignant	1	0	0	0
A2	M	40	Liver	Cholangiocellular carcinoma	Malignant	1	0	1	1
A3	M	42	Liver	Cholangiocellular carcinoma	Malignant	1	n.a.	1	1
A4	M	49	Liver	Cholangiocellular carcinoma	Malignant	1	n.a.	1	1
A5	F	59	Liver	Cholangiocellular carcinoma	Malignant	0	n.a.	0	0
A6	F	53	Liver	Cholangiocellular carcinoma	Malignant	0	n.a.	0	1
A7	M	45	Liver	Cholangiocellular carcinoma	Malignant	1	n.a.	0	1
A8	F	39	Liver	Cholangiocellular carcinoma	Malignant	n.a.	n.a.	0	1
A9	F	56	Liver	Cholangiocellular carcinoma	Malignant	n.a.	n.a.	0	1
A10	M	35	Liver	Cholangiocellular carcinoma (hyperplasia of liver and bile duct)	Malignant	n.a.	n.a.	0	1
A11	M	46	Liver	Cholangiocellular carcinoma	Malignant	n.a.	0	0	0
A12	M	66	Liver	Cholangiocellular carcinoma	Malignant	n.a.	0	0	0
B1	F	36	Liver	Cholangiocellular carcinoma	Malignant	1	1	1	0
B2	M	58	Liver	Cholangiocellular carcinoma	Malignant	1	1	1	0
B3	F	54	Liver	Cholangiocellular carcinoma with necrosis	Malignant	1	0	1	1
B4	M	52	Liver	Hepatocellular carcinoma	Malignant	1	0	2	2
B5	F	32	Liver	Hepatocellular carcinoma	Malignant	2	1	3	2
B6	M	60	Liver	Hepatocellular carcinoma	Malignant	2	1	3	2
B7	M	38	Liver	Hepatocellular carcinoma	Malignant	2	1	2	2
B8	M	52	Liver	Hepatocellular carcinoma with necrosis	Malignant	3	1	2	2

B9	F	48	Liver	Hepatocellular carcinoma	Malignant	1	0	1	2
B10	M	56	Liver	Hepatocellular carcinoma	Malignant	1	0	2	2
B11	M	35	Liver	Hepatocellular carcinoma	Malignant	2	0	1	2
B12	M	45	Liver	Hepatocellular carcinoma (chronic inflammatory cell infiltrating liver tissue)	Malignant	n.a.	n.a.	n.a.	n.a.
C1	M	35	Liver	Hepatocellular carcinoma	Malignant	2	0	2	1
C2	M	63	Liver	Hepatocellular carcinoma	Malignant	1	0	3	1
C3	M	58	Liver	Hepatocellular carcinoma (liver and lymphocyte infiltrating)	Malignant	1	1	2	1
C4	M	58	Liver	Hepatocellular carcinoma	Malignant	1	1	2	1
C5	F	37	Liver	Hepatocellular carcinoma	Malignant	0	1	1	1
C6	M	57	Liver	Hepatocellular carcinoma	Malignant	3	1	1	2
C7	M	51	Liver	Hepatocellular carcinoma	Malignant	1	0	1	2
C8	M	59	Liver	Hepatocellular carcinoma	Malignant	3	1	1	2
C9	M	43	Liver	Hepatocellular carcinoma	Malignant	1	1	2	3
C10	M	43	Liver	Hepatocellular carcinoma	Malignant	1	1	1	2
C11	M	58	Liver	Hepatocellular carcinoma	Malignant	2	0	2	2
C12	M	48	Liver	Hepatocellular carcinoma	Malignant	1	1	0	1
D1	M	40	Liver	Hepatocellular carcinoma	Malignant	2	1	1	0
D2	F	55	Liver	Hepatocellular carcinoma	Malignant	2	1	3	1
D3	M	55	Liver	Hepatocellular carcinoma	Malignant	3	1	3	1
D4	M	49	Liver	Hepatocellular carcinoma	Malignant	2	1	2	2
D5	F	52	Liver	Hepatocellular carcinoma	Malignant	1	1	1	1
D6	M	48	Liver	Hepatocellular carcinoma	Malignant	2	1	2	1
D7	M	49	Liver	Hepatocellular carcinoma	Malignant	3	1	1	2
D8	M	56	Liver	Hepatocellular carcinoma (sparse cancerous embolus in vas)	Malignant	3	0	1	2
D9	F	45	Liver	Hepatocellular carcinoma	Malignant	2	0	2	3
D10	M	43	Liver	Hepatocellular carcinoma	Malignant	3	1	2	3
D11	M	36	Liver	Hepatocellular carcinoma	Malignant	2	0	2	3
D12	M	63	Liver	Hepatocellular carcinoma	Malignant	2	1	2	2
E1	M	71	Liver	Hepatocellular carcinoma	Malignant	3	0	2	0
E2	M	55	Liver	Hepatocellular carcinoma	Malignant	2	2	3	1
E3	M	48	Liver	Hepatocellular carcinoma	Malignant	1	1	2	2
E4	M	63	Liver	Hepatocellular carcinoma	Malignant	2	1	3	2
E5	M	52	Liver	Hepatocellular carcinoma	Malignant	2	1	3	2
E6	M	48	Liver	Hepatocellular carcinoma	Malignant	2	1	3	2
E7	M	51	Liver	Hepatocellular carcinoma with necrosis	Malignant	1	0	0	2
E8	M	50	Liver	Hepatocellular carcinoma (chronic inflammation of fibrous tissue)	Malignant	3	0	0	2
E9	M	46	Liver	Hepatocellular carcinoma (sparse)	Malignant	1	1	1	3
E10	M	68	Liver	Hepatocellular carcinoma	Malignant	2	1	1	2
E11	F	50	Liver	Hepatocellular carcinoma	Malignant	1	1	1	2
E12	M	19	Liver	Hepatocellular carcinoma	Malignant	1	0	1	2
F1	F	98	Liver	Hepatocellular carcinoma	Malignant	1	0	3	0
F2	M	64	Liver	Hepatocellular carcinoma	Malignant	1	1	2	1
F3	M	50	Liver	Hepatocellular carcinoma	Malignant	1	0	1	2
F4	M	52	Liver	Hepatocellular carcinoma with necrosis	Malignant	2	1	3	2
F5	M	65	Liver	Hepatocellular carcinoma with necrosis	Malignant	3	1	1	2
F6	M	51	Liver	Hepatocellular carcinoma	Malignant	1	0	3	2
F7	M	35	Liver	Hepatocellular carcinoma with necrosis	Malignant	3	0	2	2
F8	M	77	Liver	Hepatocellular carcinoma	Malignant	1	2	3	2
F9	M	39	Liver	Hepatocellular carcinoma	Malignant	2	0	2	3

F10	M	67	Liver	Hepatocellular carcinoma	Malignant	2	1	3	2
F11	M	53	Liver	Hepatocellular carcinoma	Malignant	2	0	0	1
F12	M	60	Liver	Hepatocellular carcinoma	Malignant	1	2	1	1
G1	F	62	Liver	Hepatocellular carcinoma (fibrous tissue and blood vessel)	Malignant	2	1	1	0
G2	M	60	Liver	Hepatocellular carcinoma with necrosis	Malignant	2	0	2	1
G3	M	60	Liver	Hepatocellular carcinoma	Malignant	3	1	2	1
G4	M	35	Liver	Hepatocellular carcinoma	Malignant	2	1	3	2
G5	M	40	Liver	Hepatocellular carcinoma	Malignant	1	1	1	2
G6	M	73	Liver	Hepatocellular carcinoma	Malignant	3	0	1	1
G7	M	37	Liver	Hepatocellular carcinoma (hyperplasia of liver and bile duct)	Malignant	2	1	2	3
G8	M	33	Liver	Hepatocellular carcinoma	Malignant	1	0	1	2
G9	M	50	Liver	Hepatocellular carcinoma	Malignant	2	1	2	2
G10	M	43	Liver	Hepatocellular carcinoma	Malignant	1	1	3	2
G11	M	47	Liver	Hepatocellular carcinoma	Malignant	2	2	3	1
G12	M	53	Liver	Hepatocellular carcinoma	Malignant	1	1	2	2
H1	M	40	Liver	Hepatocellular carcinoma	Malignant	2	0	0	2
H2	F	43	Liver	Hepatocellular carcinoma	Malignant	1	0	2	3
H3	F	49	Liver	Hepatocellular carcinoma	Malignant	1	2	2	3
H4	F	43	Liver	Hepatocellular carcinoma	Malignant	1	3	2	2
H5	M	40	Liver	Hepatocellular carcinoma	Malignant	1	1	2	2
H6	M	62	Liver	Hepatocellular carcinoma	Malignant	2	1	3	1
H7	F	63	Liver	Hepatocellular carcinoma	Malignant	3	1	2	2
H8	M	38	Liver	Hepatocellular carcinoma	Malignant	3	1	2	3
H9	M	37	Liver	Hepatocellular carcinoma	Malignant	2	1	2	3
H10	M	50	Liver	Hepatocellular carcinoma	Malignant	2	1	3	3
H11	F	43	Liver	Hepatocellular carcinoma	Malignant	1	1	2	2
H12	M	61	Liver	Hepatocellular carcinoma	Malignant	2	0	1	3
I1	M	45	Liver	Hepatocellular carcinoma	Malignant	2	1	1	2
I2	F	36	Liver	Hepatocellular carcinoma (sparse)	Malignant	1	n.a.	n.a.	n.a.
I3	M	54	Liver	Hepatocellular carcinoma	Malignant	1	0	1	2
I4	M	52	Liver	Hepatocellular carcinoma	Malignant	1	0	1	2
I5	M	50	Liver	Hepatocellular carcinoma	Malignant	2	0	1	2
I6	M	51	Liver	Hepatocellular carcinoma	Malignant	1	0	2	2
I7	M	51	Liver	Hepatocellular carcinoma	Malignant	1	1	1	1
I8	F	65	Liver	Hepatocellular carcinoma	Malignant	0	2	1	2
I9	M	49	Liver	Hepatocellular carcinoma	Malignant	1	0	1	2
I10	F	69	Liver	Hepatocellular carcinoma (inflammatory cell infiltrating liver tissue)	Malignant	2	2	3	2
I11	M	55	Liver	Hepatocellular carcinoma with necrosis	Malignant	1	1	1	2
I12	M	64	Liver	Hepatocellular carcinoma with necrosis	Malignant	1	2	2	2
J1	F	52	Liver	Hepatocellular carcinoma	Malignant	0	0	0	1
J2	M	38	Liver	Hepatocellular carcinoma	Malignant	1	0	0	1
J3	M	3	Liver	Normal hepatic tissue (mild fatty degeneration of liver tissue)	Normal	1	0	1	1
J4	M	50	Liver	Normal hepatic tissue	Normal	2	1	2	2
J5	M	19	Liver	Normal hepatic tissue	Normal	2	1	2	0
J6	F	40	Liver	Normal hepatic tissue	Normal	2	3	3	1
J7	M	43	Liver	Normal hepatic tissue	Normal	2	1	2	0
J8	F	21	Liver	Normal hepatic tissue	Normal	2	1	3	1
J9	F	21	Liver	Normal hepatic tissue	Normal	2	1	3	1
J10	M	47	Liver	Normal hepatic tissue	Normal	2	1	2	1

J11	F	21	Liver	Normal hepatic tissue	Normal	2	1	2	1
J12	M	3	Liver	Normal hepatic tissue (mild congestion of liver tissue)	Normal	1	1	2	0

8. CO1503: ***Colon Cancer*** *tissue array*

position	sex	age	organ	pathology	type	TGFß1	TGFß2 (Acris)	TGFß2 (SC)	p-SMAD 2/3
A1	F	51	Colon	Adenocarcinoma	Malignant	0	0	1	2
A2	M	80	Colon	Adenocarcinoma	Malignant	1	0	1	1
A3	F	46	Colon	Adenocarcinoma	Malignant	1	1	1	2
A4	M	64	Colon	Adenocarcinoma	Malignant	2	1	2	2
A5	M	34	Colon	Mucinous adenocarcinoma	Malignant	0	n.a.	1	0
A6	M	51	Colon	Adenocarcinoma	Malignant	n.a.	n.a.	1	1
A7	F	60	Colon	Adenocarcinoma	Malignant	n.a.	n.a.	2	1
A8	M	68	Colon	Adenocarcinoma	Malignant	n.a.	n.a.	2	1
A9	F	43	Colon	Adenocarcinoma	Malignant	n.a.	n.a.	1	0
A10	F	64	Colon	Adenocarcinoma	Malignant	n.a.	n.a.	1	0
A11	F	78	Colon	Adenocarcinoma	Malignant	n.a.	n.a.	1	1
A12	F	46	Colon	Adenocarcinoma	Malignant	n.a.	n.a.	1	1
A13	F	52	Colon	Adenocarcinoma	Malignant	n.a.	n.a.	1	1
A14	M	38	Colon	Adenocarcinoma	Malignant	n.a.	n.a.	1	0
A15	M	44	Colon	Adenocarcinoma	Malignant	n.a.	n.a.	0	0
B1	F	51	Colon	Adenocarcinoma	Malignant	1	1	1	2
B2	M	80	Colon	Adenocarcinoma	Malignant	2	0	0	0
B3	F	46	Colon	Adenocarcinoma	Malignant	1	1	1	2
B4	M	64	Colon	Adenocarcinoma	Malignant	2	1	2	2
B5	M	34	Colon	Mucinous adenocarcinoma	Malignant	1	1	n.a.	1
B6	M	51	Colon	Adenocarcinoma	Malignant	1	1	1	2
B7	F	60	Colon	Adenocarcinoma	Malignant	n.a.	1	2	2
B8	M	68	Colon	Adenocarcinoma	Malignant	n.a.	n.a.	2	2
B9	F	43	Colon	Adenocarcinoma	Malignant	n.a.	n.a.	2	0
B10	F	64	Colon	Adenocarcinoma	Malignant	n.a.	n.a.	1	0
B11	F	78	Colon	Adenocarcinoma	Malignant	n.a.	n.a.	1	1
B12	F	46	Colon	Adenocarcinoma	Malignant	n.a.	n.a.	1	1
B13	F	52	Colon	Adenocarcinoma	Malignant	n.a.	n.a.	1	1
B14	M	38	Colon	Adenocarcinoma	Malignant	n.a.	n.a.	1	1
B15	M	44	Colon	Adenocarcinoma	Malignant	n.a.	n.a.	1	2
C1	M	66	Colon	Adenocarcinoma	Malignant	1	2	2	1
C2	F	45	Colon	Mucinous adenocarcinoma	Malignant	1	2	2	1
C3	F	74	Colon	Mucinous adenocarcinoma	Malignant	1	1	2	2
C4	F	63	Colon	Adenocarcinoma	Malignant	1	1	3	2
C5	F	50	Colon	Adenocarcinoma	Malignant	1	2	2	1
C6	F	64	Colon	Adenocarcinoma	Malignant	0	1	1	2
C7	F	63	Colon	Adenocarcinoma	Malignant	n.a.	0	1	2
C8	F	79	Colon	Adenocarcinoma	Malignant	n.a.	2	2	3
C9	F	58	Colon	Adenocarcinoma	Malignant	n.a.	n.a.	1	2
C10	F	74	Colon	Mucinous adenocarcinoma	Malignant	n.a.	n.a.	0	1
C11	M	46	Colon	Adenocarcinoma	Malignant	n.a.	2	2	2
C12	M	72	Colon	Adenocarcinoma	Malignant	n.a.	n.a.	2	3
C13	M	59	Colon	Undifferentiated carcinoma	Malignant	n.a.	n.a.	1	1

C14	M	53	Colon	Adenocarcinoma	Malignant	n.a.	n.a.	1	1
C15	F	57	Colon	Adenocarcinoma	Malignant	n.a.	n.a.	1	3
D1	M	66	Colon	Adenocarcinoma	Malignant	1	1	1	1
D2	F	45	Colon	Mucinous adenocarcinoma	Malignant	1	1	1	1
D3	F	74	Colon	Mucinous adenocarcinoma	Malignant	1	0	1	1
D4	F	63	Colon	Adenocarcinoma	Malignant	1	1	2	2
D5	F	50	Colon	Adenocarcinoma	Malignant	1	2	2	2
D6	F	64	Colon	Adenocarcinoma	Malignant	0	1	1	3
D7	F	63	Colon	Adenocarcinoma	Malignant	0	1	3	2
D8	F	79	Colon	Adenocarcinoma	Malignant	n.a.	2	2	3
D9	F	58	Colon	Adenocarcinoma	Malignant	n.a.	1	1	3
D10	F	74	Colon	Mucinous adenocarcinoma (smooth muscle)	Malignant	n.a.	1	1	2
D11	M	46	Colon	Adenocarcinoma	Malignant	n.a.	2	2	2
D12	M	72	Colon	Adenocarcinoma	Malignant	n.a.	1	2	2
D13	M	59	Colon	Adenocarcinoma	Malignant	n.a.	1	1	1
D14	M	53	Colon	Adenocarcinoma	Malignant	n.a.	1	1	2
D15	F	57	Colon	Adenocarcinoma	Malignant	n.a.	n.a.	1	3
E1	F	70	Colon	Adenocarcinoma	Malignant	3	1	1	1
E2	M	45	Colon	Adenocarcinoma	Malignant	1	1	1	1
E3	F	69	Colon	Adenocarcinoma	Malignant	1	1	2	1
E4	M	82	Colon	Adenocarcinoma (fibrofatty tissue)	Malignant	1	1	n.a.	1
E5	M	71	Colon	Adenocarcinoma	Malignant	1	1	1	3
E6	M	40	Colon	Adenocarcinoma	Malignant	1	0	2	2
E7	M	51	Colon	Adenocarcinoma with necrosis	Malignant	2	0	1	1
E8	F	53	Colon	Mucinous adenocarcinoma	Malignant	1	1	1	1
E9	M	49	Colon	Adenocarcinoma	Malignant	0	1	1	2
E10	M	55	Colon	Adenocarcinoma	Malignant	n.a.	0	1	2
E11	M	67	Colon	Adenocarcinoma	Malignant	n.a.	1	1	0
E12	M	72	Colon	Adenocarcinoma	Malignant	n.a.	2	2	3
E13	M	36	Colon	Adenocarcinoma	Malignant	n.a.	1	1	1
E14	M	43	Colon	Adenocarcinoma	Malignant	n.a.	0	1	1
E15	F	50	Colon	Adenocarcinoma	Malignant	n.a.	1	1	1
F1	F	70	Colon	Adenocarcinoma	Malignant	2	1	1	1
F2	M	45	Colon	Adenocarcinoma	Malignant	1	1	1	1
F3	F	69	Colon	Adenocarcinoma	Malignant	1	0	2	1
F4	M	82	Colon	Adenocarcinoma	Malignant	2	0	1	1
F5	M	71	Colon	Adenocarcinoma	Malignant	1	1	2	2
F6	M	40	Colon	Adenocarcinoma	Malignant	1	1	2	2
F7	M	51	Colon	Adenocarcinoma with necrosis (sparse)	Malignant	3	1	2	1
F8	F	53	Colon	Mucinous adenocarcinoma (tumoral necrosis)	Malignant	1	0	0	0
F9	M	49	Colon	Adenocarcinoma	Malignant	1	1	1	2
F10	M	55	Colon	Adenocarcinoma	Malignant	1	1	1	2
F11	M	67	Colon	Mucinous adenocarcinoma	Malignant	1	1	0	0
F12	M	72	Colon	Adenocarcinoma	Malignant	0	2	2	2
F13	M	36	Colon	Adenocarcinoma	Malignant	0	1	1	1
F14	M	43	Colon	Adenocarcinoma	Malignant	n.a.	1	1	0
F15	F	50	Colon	Adenocarcinoma	Malignant	n.a.	1	1	2
G1	M	51	Colon	Adenocarcinoma	Malignant	2	1	1	2
G2	M	46	Colon	Adenocarcinoma	Malignant	2	1	2	3
G3	M	37	Colon	Adenocarcinoma	Malignant	2	2	3	3
G4	M	70	Colon	Mucinous adenocarcinoma	Malignant	1	1	1	2

G5	F	48	Colon	Adenocarcinoma	Malignant	2	1	1	3
G6	F	34	Colon	Adenocarcinoma	Malignant	1	2	1	2
G7	M	50	Colon	Adenocarcinoma with necrosis	Malignant	2	0	1	1
G8	F	48	Colon	Adenocarcinoma	Malignant	1	0	1	2
G9	F	73	Colon	Adenocarcinoma	Malignant	2	0	1	1
G10	M	30	Colon	Adenocarcinoma	Malignant	3	0	0	1
G11	M	48	Colon	Adenocarcinoma	Malignant	1	1	1	1
G12	F	76	Colon	Adenocarcinoma	Malignant	1	0	1	2
G13	M	40	Colon	Adenocarcinoma	Malignant	1	1	1	1
G14	M	65	Colon	Adenocarcinoma with necrosis	Malignant	2	0	0	1
G15	F	81	Colon	Signet-ring cell carcinoma	Malignant	0	0	1	1
H1	M	51	Colon	Adenocarcinoma	Malignant	1	n.a.	1	2
H2	M	46	Colon	Adenocarcinoma	Malignant	1	n.a.	1	2
H3	M	37	Colon	Adenocarcinoma	Malignant	2	n.a.	3	3
H4	M	70	Colon	Mucinous adenocarcinoma (sparse)	Malignant	1	n.a.	2	1
H5	F	48	Colon	Adenocarcinoma	Malignant	2	0	1	3
H6	F	34	Colon	Adenocarcinoma	Malignant	1	0	1	2
H7	M	50	Colon	Adenocarcinoma	Malignant	1	n.a.	1	2
H8	F	48	Colon	Adenocarcinoma	Malignant	2	n.a.	1	1
H9	F	73	Colon	Adenocarcinoma	Malignant	2	0	2	1
H10	M	30	Colon	Adenocarcinoma	Malignant	3	0	0	1
H11	M	48	Colon	Adenocarcinoma	Malignant	1	1	1	1
H12	F	76	Colon	Adenocarcinoma	Malignant	1	0	1	1
H13	M	40	Colon	Adenocarcinoma	Malignant	2	0	1	2
H14	M	65	Colon	Adenocarcinoma with necrosis	Malignant	2	0	0	0
H15	F	81	Colon	Signet-ring cell carcinoma	Malignant	1	1	1	1
I1	M	56	Colon	Mucinous adenocarcinoma	Malignant	1	n.a.	0	1
I2	F	60	Colon	Adenocarcinoma	Malignant	2	n.a.	2	1
I3	M	74	Colon	Adenocarcinoma	Malignant	1	n.a.	1	2
I4	M	55	Colon	Adenocarcinoma	Malignant	1	n.a.	1	2
I5	F	60	Colon	Adenocarcinoma	Malignant	2	n.a.	2	0
I6	M	56	Colon	Adenocarcinoma	Malignant	1	n.a.	1	2
I7	M	70	Colon	Adenocarcinoma	Malignant	1	n.a.	1	0
I8	F	82	Colon	Adenocarcinoma	Malignant	1	n.a.	1	1
I9	M	33	Colon	Adenocarcinoma	Malignant	2	n.a.	1	2
I10	F	69	Colon	Metastatic adenocarcinoma from ovary	Metastasis	1	1	2	2
J1	M	56	Colon	Mucinous adenocarcinoma	Malignant	0	n.a.	1	1
J2	F	60	Colon	Adenocarcinoma	Malignant	2	n.a.	1	1
J3	M	74	Colon	Adenocarcinoma	Malignant	1	n.a.	1	2
J4	M	55	Colon	Adenocarcinoma	Malignant	2	n.a.	1	1
J5	F	60	Colon	Adenocarcinoma	Malignant	1	n.a.	2	0
J6	M	56	Colon	Adenocarcinoma	Malignant	2	n.a.	1	2
J7	M	70	Colon	Adenocarcinoma	Malignant	2	n.a.	1	0
J8	F	82	Colon	Adenocarcinoma	Malignant	1	n.a.	1	1
J9	M	33	Colon	Adenocarcinoma	Malignant	n.a.	n.a.	1	2
J10	F	69	Colon	Metastatic adenocarcinoma from ovary	Metastasis	n.a.	n.a.	3	1
I11	M	82	Colon	Adenoma	Benign	0	2	2	2
I12	M	70	Colon	Adenoma	Benign	1	2	2	2
I13	M	31	Colon	Polyp	Polyp	3	2	2	1
I14	F	27	Colon	Polyp	Polyp	1	0	1	3
J11	M	82	Colon	Adenoma	Benign	n.a.	n.a.	2	2
J12	M	70	Colon	Adenoma	Benign	1	n.a.	2	2

J13	M	31	Colon	Polyp	Polyp	2	n.a.	2	1
J14	F	27	Colon	Polyp	Polyp	1	n.a.	1	2
I15	M	25	Colon	Normal colon tissue	Normal	2	1	1	1
J15	M	25	Colon	Normal colon tissue	Normal	1	n.a.	1	1

*9. HN483: **Multiple head and neck cancer** with normal tissue array*

position	sex	age	organ	pathology	type	TGFß1	TGFß2 (Acris)	TGFß2 (SC)	p-SMAD 2/3
A1	M	67	Palate	Squamous cell carcinoma of right palate	Malignant	0	0	1	2
A2	M	50	Tongue	Squamous cell carcinoma of root of tongue	Malignant	1	1	1	2
A3	M	41	Lower lip	Squamous cell carcinoma	Malignant	1	1	2	2
A4	M	70	Lower lip	Squamous cell carcinoma	Malignant	1	0	1	2
A5	M	51	Upper jaw	Squamous cell carcinoma of right upper jaw	Malignant	n.a.	n.a.	1	1
A6	M	43	Maxillary sinus	Squamous cell carcinoma of maxillary sinus	Malignant	1	1	2	1
A7	F	41	Upper jaw	Squamous cell carcinoma	Malignant	1	n.a.	1	1
A8	M	51	Lower lip	Squamous cell carcinoma	Malignant	n.a.	n.a.	0	1
B1	M	57	Upper jaw	Squamous cell carcinoma of right upper jaw	Malignant	2	0	2	1
B2	M	61	Gingiva	Squamous cell carcinoma of left mandibular gingiva	Malignant	2	1	1	1-0
B3	M	54	Larynx	Squamous cell carcinoma	Malignant	1	1	1	1
B4	M	67	Larynx	Squamous cell carcinoma	Malignant	1	1	2	2
B5	M	50	Tongue	Squamous cell carcinoma	Malignant	1	0	0	2
B6	F	36	Gingiva	Squamous cell carcinoma of right mandibular gingiva	Malignant	1	0	0	1
B7	M	67	Larynx	Squamous cell carcinoma	Malignant	1	1	2	1
B8	M	63	Larynx	Squamous cell carcinoma	Malignant	1	1	2	1
C1	M	72	Larynx	Squamous cell carcinoma	Malignant	1	1	2	2
C2	M	50	Larynx	Squamous cell carcinoma	Malignant	2	0	2	1
C3	M	56	Epiglottis	Squamous cell carcinoma	Malignant	1	0	2	3
C4	M	54	Larynx	Squamous cell carcinoma	Malignant	0	0	0	2
C5	M	43	Submaxilla	Squamous cell carcinoma of right submaxilla	Malignant	1	2	1	2
C6	M	50	Parotid gland	Squamous cell carcinoma with necrosis of right parotid gland	Malignant	1	1	2	1
C7	M	62	Larynx	Squamous cell carcinoma (chronic inflammation of fibrous tissue)	Malignant	2	0	1	1
C8	M	55	Larynx	Squamous cell carcinoma	Malignant	1	1	1	1
D1	M	49	Larynx	Squamous cell carcinoma	Malignant	1	0	2	1
D2	M	58	Larynx	Squamous cell carcinoma	Malignant	1	0	1	1
D3	M	61	Larynx	Squamous cell carcinoma	Malignant	1	2	3	2
D4	M	57	Larynx	Squamous cell carcinoma (chronic inflammation of larync tissue)	Malignant	1	1	2	1
D5	F	58	Larynx	Squamous cell carcinoma with necrosis	Malignant	1	1	2	2
D6	M	56	Laryngeal pharynx	Squamous cell carcinoma	Malignant	1	0	3	3
D7	F	52	Larynx	Squamous cell carcinoma with necrosis	Malignant	1	1	2	2
D8	M	55	Larynx	Squamous cell carcinoma	Malignant	2	1	2	2
E1	M	42	Larynx	Squamous cell carcinoma	Malignant	1	0	2	1
E2	M	52	Larynx	Squamous cell carcinoma	Malignant	1	2	2	2
E3	F	62	Maxillary	Squamous cell carcinoma	Malignant	1	2	1	2

			sinus						
E4	M	41	Maxillary sinus	Squamous cell carcinoma	Malignant	0	2	1	2
E5	M	50	Gingiva	Squamous cell carcinoma	Malignant	1	1	1	2
E6	M	65	Larynx	Squamous cell carcinoma	Malignant	1	1	1	2
E7	M	59	Larynx	Squamous cell carcinoma	Malignant	1	1	2	1
E8	M	41	Nasopharynx	Squamous cell carcinoma	Malignant	2	0	0	2
F1	M	50	Tongue	Normal tongue tissue	Normal	1	1	2	2
F2	F	42	Tongue	Normal tongue tissue	Normal	0	0	1	1
F3	M	46	Tongue	Normal tongue tissue	Normal	n.a.	1	1	1
F4	F	21	Tongue	Normal tongue tissue	Normal	n.a.	n.a.	2	1
F5	M	43	Pharynx	Normal pharynx tissue	Normal	n.a.	0	1	1
F6	F	15	Laryngopharynx	Normal Laryngopharynx tissue	Normal	n.a.	1	2	1
F7	M	45	Larynx	Normal pharynx tissue	Normal	n.a.	1	2	1
F8	M	16	Epiglottis	Normal epiglottis tissue (cartilage, fibrous tissue and blood vessel)	Normal	0	0	1	1

*10. ME2082b: **Malignant Melanoma** tissue array*

position	sex	age	organ	pathology	type	TGFß1	TGFß2 (Acris)	TGFß2 (SC)	p-SMAD 2/3
A1	M	40	Pleura	Malignant melanoma of right chest wall	Malignant	1	0	1	0
A2	M	50	Esophagus	Malignant melanoma of esophagus	Malignant	0	0	1	0
A3	M	64	Esophagus	Malignant melanoma of esophagus	Malignant	1	0	2	2
A4	M	71	Intestine	Malignant melanoma of small intestine	Malignant	2	2	3	3
A5	F	70	Tongue	Malignant melanoma of left parotid	Malignant	2	1	2	3
A6	F	38	Rectum	Malignant melanoma of rectum	Malignant	1	0	1	3
A7	F	67	Rectum	Malignant melanoma of rectum	Malignant	0	0	1	3
A8	F	70	Rectum	Malignant melanoma with necrosis of anus	Malignant	2(Fibrine +)	2(Fibrine +)	0(Fibrine +)	0
A9	M	66	Rectum	Malignant melanoma of rectum	Malignant	2	0	0	0
A10	F	54	Rectum	Malignant melanoma of rectum	Malignant	2	1	2	2
A11	F	82	Rectum	Malignant melanoma of rectum	Malignant	0	1	2	2
A12	F	52	Rectum	Malignant melanoma of rectum	Malignant	0	1	2	3
A13	F	72	Rectum	Malignant melanoma of rectum	Malignant	0	0	2	3
A14	F	69	Rectum	Malignant melanoma of rectum	Malignant	0	0	1	1
A15	M	64	Rectum	Malignant melanoma of rectum	Malignant	1	1	0	2
A16	F	52	Rectum	Malignant melanoma of rectum (sparse)	Malignant	1	0	1	1 (cytopl.)
B1	F	42	Rectum	Malignant melanoma of rectum	Malignant	0	0	1	1
B2	M	47	Rectum	Malignant melanoma of rectum	Malignant	1	1	2	3
B3	M	75	Rectum	Malignant melanoma of rectum	Malignant	2	1	1	3
B4	M	67	Rectum	Malignant melanoma of rectum	Malignant	0	0	2	2
B5	F	66	Rectum	Malignant melanoma of rectum	Malignant	3	0	1	3
B6	M	55	Stomach	Malignant melanoma of stomach	Malignant	2	1	2	2
B7	M	55	Stomach	Malignant melanoma of stomach	Malignant	0	0	3	2
B8	F	44	Uterine cervix	Malignant melanoma of cervix	Malignant	1	0	2	3
B9	F	57	Vulva	Malignant melanoma of cunnus	Malignant	0	0	0	0
B10	F	45	Vulva	Malignant melanoma of cunnus	Malignant	0	0	0	0
B11	F	72	Vulva	Malignant melanoma of cunnus	Malignant	0	0	3	2

B12	F	44	Vulva	Malignant melanoma of cunnus	Malignant	0	0	2	2
B13	F	42	Vulva	Malignant melanoma of cunnus	Malignant	0	0	1	3
B14	F	62	Vulva	Malignant melanoma of vagina	Malignant	2	0	0	0
B15	F	41	Vulva	Malignant melanoma of cunnus	Malignant	2	1	0	2
B16	F	38	Vulva	Malignant melanoma of cunnus	Malignant	0	0	0	1
C1	M	52	Skin	Malignant melanoma of right heel	Malignant	0	0	1	2
C2	M	71	Skin	Malignant melanoma of right groin	Malignant	1	1	3	1
C3	F	62	Skin	Malignant melanoma of right thumb	Malignant	0	0	1	3
C4	F	46	Skin	Malignant melanoma of thigh	Malignant	0	0	1	2
C5	M	66	Skin	Malignant melanoma of right thigh (sparse)	Malignant	1	1	2	3
C6	F	66	Skin	Malignant melanoma of chest wall	Malignant	1	0	1	3
C7	M	56	Skin	Malignant melanoma of abdominal wall	Malignant	0	1	1	1
C8	M	61	Skin	Malignant melanoma of right sole	Malignant	1	0	3	3
C9	F	62	Urethra	Malignant melanoma of urethra (fibrous tissue and blood vessel)	Malignant	2	0	n.a.	n.a.
C10	M	52	Skin	Malignant melanoma of left crissum	Malignant	2	0	1	2
C11	F	47	Skin	Malignant melanoma of right upper arm	Malignant	1	1	2	3
C12	M	57	Skin	Malignant melanoma of left shoulder	Malignant	1	0	1	1
C13	M	64	Skin	Malignant melanoma of sole	Malignant	0	0	1	2
C14	M	53	Skin	Malignant melanoma of right sole	Malignant	0	0	1	0
C15	F	59	Skin	Malignant melanoma of left upper limb (tumoral necrosis)	Malignant	1	0	2	1
C16	F	73	Skin	Malignant melanoma of right big toe	Malignant	3	0	1	2
D1	M	65	Skin	Malignant melanoma of scalp	Malignant	0	0	0	0
D2	M	37	Skin	Malignant melanoma of right upper arm	Malignant	2	1	1	1
D3	F	83	Skin	Malignant melanoma of right little finger	Malignant	1	0	2	2
D4	F	59	Skin	Malignant melanoma of right rump	Malignant	0	0	0	0
D5	M	42	Skin	Malignant melanoma of left sole	Malignant	0	1	1	2
D6	M	65	Skin	Malignant melanoma of right thumb	Malignant	0	0	2	2
D7	M	76	Skin	Malignant melanoma of right medial malleolus	Malignant	0	0	1	1
D8	F	54	Skin	Malignant melanoma of left heel	Malignant	1	0	3	1
D9	M	41	Skin	Malignant melanoma of left forearm	Malignant	2	0	1	1
D10	M	55	Skin	Malignant melanoma of right forearm	Malignant	0	0	2	2
D11	F	43	Skin	Malignant melanoma of left leg	Malignant	0	0	0	0
D12	M	65	Skin	Malignant melanoma of right sole	Malignant	0	2	2	2
D13	M	31	Skin	Malignant melanoma of scalp	Malignant	0	0	1	1
D14	F	41	Skin	Malignant melanoma with keratinization of scalp (sparse)	Malignant	n.a.	n.a.	n.a.	n.a.
D15	M	74	Skin	Malignant melanoma of left foot	Malignant	0	0	1	2
D16	M	21	Skin	Malignant melanoma of back	Malignant	0	1	1	0
E1	F	32	Skin	Malignant melanoma of right lumbar part	Malignant	1	1	1	0
E2	F	63	Skin	Malignant melanoma of right foot	Malignant	1	0	1	1
E3	M	51	Skin	Malignant melanoma of back	Malignant	0	0	2	1
E4	M	73	Skin	Malignant melanoma of right sole	Malignant	0	2	3	2
E5	M	55	Skin	Malignant melanoma of sole	Malignant	0	0	2	1
E6	M	72	Skin	Malignant melanoma of left heel	Malignant	0	0	3	0
E7	M	60	Skin	Malignant melanoma of right rump	Malignant	0	0	2	2
E8	M	40	Skin	Malignant melanoma of right chest wall	Malignant	2	2	2	3
E9	F	77	Skin	Malignant melanoma of sole	Malignant	2	2	2	1
E10	F	56	Skin	Malignant melanoma with tumoral necrosis of left leg	Malignant	0	1	n.a.	n.a.
E11	M	42	Skin	Malignant melanoma of right sole	Malignant	0	0	2	1

E12	M	51	Skin	Malignant melanoma of left oxter	Malignant	2	0	1	0
E13	M	31	Skin	Malignant melanoma of left sole	Malignant	0	0	0	2
E14	M	61	Skin	Malignant melanoma of left oxter	Malignant	3	0	1	1
E15	M	49	Skin	Malignant melanoma of left rump	Malignant	0	0	1	3
E16	F	46	Skin	Malignant melanoma of right big toe	Malignant	0	1	2(nuclear)	1
F1	M	80	Skin	Malignant melanoma of right sole	Malignant	1	0	2	1
F2	F	74	Skin	Malignant melanoma of right heel	Malignant	0	0	1	1
F3	M	41	Skin	Malignant melanoma of left leg	Malignant	1	0	2	1
F4	M	57	Skin	Malignant melanoma of right sole	Malignant	2	0	2	2
F5	M	51	Skin	Malignant melanoma of chest wall	Malignant	0	0	0	3
F6	M	51	Skin	Malignant melanoma of left upper arm	Malignant	2	0	1	0
F7	M	52	Skin	Malignant melanoma of abdomen	Malignant	1	0	2	3
F8	M	45	Skin	Malignant melanoma of crissum	Malignant	2	0	3	3
F9	M	61	Skin	Malignant melanoma of right groin	Malignant	3	1	2	2
F10	F	61	Skin	Malignant melanoma of left sole	Malignant	0	1	2	1
F11	F	59	Skin	Malignant melanoma of anus	Malignant	1	0	2	2
F12	M	49	Skin	Malignant melanoma of left foot	Malignant	0	0	0	0
F13	F	50	Skin	Malignant melanoma of back	Malignant	0	1	1	1
F14	M	36	Skin	Malignant melanoma of back	Malignant	1	1	2	3
F15	M	46	Scrotum	Malignant melanoma of scrotum	Malignant	1	1	1	1
F16	M	55	Scrotum	Malignant melanoma of scrotum (saprse)	Malignant	n.a.	n.a.	n.a.	n.a.
G1	M	78	Nose	Malignant melanoma of left nasal cavity	Malignant	0	0	1	0
G2	M	50	Nose	Malignant melanoma of right nose	Malignant	2	1	1	1
G3	F	47	Nose	Malignant melanoma of right nasal cavity	Malignant	1	0	3	2
G4	F	38	Nose	Malignant melanoma of nasal cavity	Malignant	0	1	1	1
G5	M	56	Nose	Malignant melanoma of nasal cavity (tumoral necrosis)	Malignant	n.a.	n.a.	n.a.	n.a.
G6	M	63	Nose	Malignant melanoma of nasal cavity	Malignant	3	2	2	1
G7	M	45	Skin	Malignant melanoma of right sole	Malignant	0	0	n.a.	n.a.
G8	F	43	Fibrous tissue	Malignant melanoma of right upper abdominal wall	Malignant	1	1	1	1
G9	F	46	Adipose tissue	Malignant melanoma of right chest wall	Malignant	0	0	0	n.a.
G10	F	42	Skin	Malignant melanoma of left thumb	Malignant	0	0	1	1
G11	F	58	Skin	Malignant melanoma of left rump	Malignant	0	0	3	2
G12	F	88	Skin	Malignant melanoma of left sole	Malignant	0	0	2(nuclear)	2
G13	M	7	Striated muscle	Malignant melanoma of sacrococcygeal region	Malignant	0	0	3	2
G14	M	50	Striated muscle	Malignant melanoma of left shoulder	Malignant	1	0	1	1
G15	M	71	Soft tissue	Malignant melanoma of left rump	Malignant	0	0	1	3
G16	F	45	Soft tissue	Malignant melanoma of left thigh	Malignant	0	1	2	2
H1	M	49	Soft tissue	Malignant melanoma of left thigh	Malignant	0	0	1	0
H2	F	42	Soft tissue	Malignant melanoma of right thigh	Malignant	2	2	2	1
H3	F	67	Soft tissue	Malignant melanoma of right armpit	Malignant	1	1	2	2
H4	F	67	Eye	Malignant melanoma of right eye (sparse)	Malignant	2	0	2	0
H5	F	46	Eye	Malignant melanoma of right eye	Malignant	0	0	0	0
H6	F	49	Eye	Malignant melanoma of right eye	Malignant	1	2	2	2
H7	M	52	Eye	Malignant melanoma of left eye	Malignant	1	1	1	2
H8	M	44	Eye	Malignant melanoma of left eye	Malignant	0	0	0	0
H9	M	57	Eye	Malignant melanoma of left eye	Malignant	1	2	1	0
H10	F	35	Eye	Malignant melanoma of right eye	Malignant	0	0	1	0

H11	F	62	Eye	Malignant melanoma of right eye	Malignant	0	2	1	0
H12	M	67	Eye	Malignant melanoma of right eye	Malignant	0	0	0	0
H13	F	76	Eye	Malignant melanoma of right eye	Malignant	0	3	2	2
H14	M	41	Eye	Malignant melanoma of right eye	Malignant	0	0	0	0
H15	F	37	Eye	Malignant melanoma of left eye	Malignant	1	2	2	1
H16	M	57	Eye	Malignant melanoma of right eye	Malignant	0	0	1	0
I1	M	70	Lymph node	Metastatic malignant melanoma of groin	Metastasis	1	0	1	3
I2	M	46	Lymph node	Metastatic malignant melanoma of right groin	Metastasis	0	0	2	1
I3	M	44	Lymph node	Metastatic malignant melanoma of right oxter	Metastasis	1	1	2	2
I4	M	65	Lymph node	Metastatic malignant melanoma of right groin	Metastasis	0	0	1	0
I5	M	38	Lymph node	Metastatic malignant melanoma of left armpit	Metastasis	1	0	1	2
I6	M	55	Lymph node	Metastatic malignant melanoma of left groin	Metastasis	0	0	0	0
I7	F	40	Lymph node	Metastatic malignant melanoma of right groin	Metastasis	3	0	0	0
I8	M	70	Lymph node	Metastatic malignant melanoma of armpit	Metastasis	2	0	1	2
I9	M	68	Lymph node	Metastatic malignant melanoma of neck	Metastasis	0	0	0	1
I10	F	60	Lymph node	Metastatic malignant melanoma of right groin (fibrous tissue and blood vessel)	Metastasis	0	0	0	2
I11	M	65	Lymph node	Metastatic malignant melanoma of groin	Metastasis	1	0	2	2
I12	F	61	Lymph node	Metastatic malignant melanoma of right groin	Metastasis	0	0	1	0
I13	F	42	Lymph node	Metastatic malignant melanoma of groin	Metastasis	1	2	1	2
I14	F	43	Lymph node	Metastatic malignant melanoma of groin	Metastasis	2	1	2	2
I15	F	56	Lymph node	Metastatic malignant melanoma of right groin	Metastasis	2	0	2	2
I16	F	56	Lymph node	Metastatic malignant melanoma of groin	Metastasis	0	1	1	1
J1	F	49	Lymph node	Metastatic malignant melanoma of neck	Metastasis	0	0	1	0
J2	M	49	Lymph node	Metastatic malignant melanoma of left oxter	Metastasis	0	0	0	2
J3	M	45	Lymph node	Metastatic malignant melanoma of right oxter	Metastasis	3	0	0	1
J4	F	57	Lymph node	Metastatic malignant melanoma of right groin	Metastasis	0	0	2	1
J5	M	57	Lymph node	Metastatic malignant melanoma of neck	Metastasis	0	0	0	2
J6	F	29	Lymph node	Metastatic malignant melanoma of neck	Metastasis	0	1	1	0
J7	F	31	Lymph node	Metastatic malignant melanoma of neck	Metastasis	0	0	1	0
J8	M	62	Lymph node	Metastatic malignant melanoma of left groin	Metastasis	0	1	2	1
J9	F	63	Lymph node	Metastatic malignant melanoma of right groin	Metastasis	1	1	2	0
J10	F	61	Lymph node	Metastatic malignant melanoma of right groin	Metastasis	2	0	2	1
J11	M	68	Lymph node	Metastatic malignant melanoma of right armpit	Metastasis	0	1	1	0
J12	F	60	Lymph node	Metastatic malignant melanoma of neck	Metastasis	0	0	1	1
J13	M	52	Lymph	Metastatic malignant melanoma of left	Metastasis	2	0	3	1

			node	groin					
J14	F	53	Lymph node	Metastatic malignant melanoma of right groin	Metastasis	2	0	1	2
J15	F	68	Lymph node	Metastatic malignant melanoma of left groin	Metastasis	1	1	1	1
J16	F	68	Lymph node	Metastatic malignant melanoma of right groin	Metastasis	0	1	2	1
K1	M	55	Lymph node	Metastatic malignant melanoma of left thigh	Metastasis	0	0	0	0
K2	F	58	Lymph node	Metastatic malignant melanoma of left groin	Metastasis	1	0	2	1
K3	F	38	Lymph node	Metastatic malignant melanoma of right groin	Metastasis	2	2	2	1
K4	M	49	Lymph node	Metastatic malignant melanoma of neck	Metastasis	0	0	1	2
K5	F	46	Lymph node	Metastatic malignant melanoma of right neck (sparse)	Metastasis	0	2	3	2
K6	M	50	Esophagus	Metastatic malignant melanoma of beside esophagus	Metastasis	0	0	2	0
K7	M	50	Tongue	Metastatic malignant melanoma of right beside parotid (lymph node tissue)	Metastasis	0	0	n.a.	n.a.
K8	F	47	Skin	Metastatic malignant melanoma of oxter	Metastasis	2	2	2	0
K9	M	38	Skin	Metastatic malignant melanoma of oxter	Metastasis	1	0	3	2
K10	F	45	Skin	Metastatic malignant melanoma of left chest wall	Metastasis	0	0	1	2
K11	F	41	Skin	Metastatic malignant melanoma of groin (sparse)	Metastasis	1	0	1	0
K12	F	38	Skin	Metastatic malignant melanoma of left upper arm	Metastasis	0	1	1	1
K13	M	58	Skin	Metastatic malignant melanoma of left groin	Metastasis	0	0	2	0
K14	F	45	Spleen	Metastatic malignant melanoma of spleen	Metastasis	1	0	1	3
K15	F	52	Spleen	Metastatic malignant melanoma of spleen	Metastasis	2	0	1	2
K16	F	41	Soft tissue	Metastatic malignant melanoma of groin	Metastasis	0	0	0	0
L1	M	39	Bone	Metastatic malignant melanoma of left femur inferior segment	Metastasis	0	0	1	1
L2	M	62	Scrotum	Metastatic malignant melanoma of groin	Metastasis	0	3	2	2
L3	F	55	Cerebrum	Metastatic malignant melanoma of right occipital lobe of brain	Metastasis	1	1	3	0
L4	M	63	Adipose tissue	Metastatic malignant melanoma of right neck	Metastasis	1	0	2	1
L5	M	48	Skin	Metastatic malignant melanoma of neck	Metastasis	0	1	2	2
L6	F	55	Skin	Metastatic malignant melanoma of left groin	Metastasis	2	0	2	1
L7	M	49	Skin	Metastatic malignant melanoma of cavitas pelvis	Metastasis	0	0	1	1
L8	M	72	Skin	Metastatic malignant melanoma of right groin	Metastasis	3	0	3	0
L9	M	62	Skin	Metastatic malignant melanoma of left neck	Metastasis	0	1	3	1
L10	F	35	Soft tissue	Metastatic malignant melanoma of left oxter	Metastasis	1	0	1	0
L11	F	61	Soft tissue	Metastatic malignant melanoma of right groin	Metastasis	0	0	2	0
L12	F	80	Soft tissue	Metastatic malignant melanoma of right armpit	Metastasis	0	1	1	2
L13	M	56	Soft tissue	Metastatic malignant melanoma of left preauricula	Metastasis	0	1	1	1
L14	F	72	Soft tissue	Metastatic malignant melanoma of left groin	Metastasis	0	0	0	0
L15	F	41	Soft tissue	Metastatic malignant melanoma of right groin	Metastasis	0	0	1	2

L16	M	62	Soft tissue	Metastatic malignant melanoma of groin	Metastasis	1	1	3	1
M1	F	44	Breast	Cancer adjacent normal skin tissue	NAT	Basal cell lamina of the epidermis and vessels partly positive	Basal cell lamina of the epidermis partly positive	Basal cell lamina of the epidermis partly positive	Epithelial cell nuclei partly positive
M2	F	44	Breast	Cancer adjacent normal skin tissue	NAT				
M3	F	48	Breast	Cancer adjacent normal skin tissue	NAT				
M4	F	43	Breast	Cancer adjacent normal skin tissue	NAT				
M5	F	41	Breast	Cancer adjacent normal skin tissue	NAT				
M6	F	43	Breast	Cancer adjacent normal skin tissue	NAT				
M7	F	37	Skin	Cancer adjacent normal skin tissue	NAT				
M8	M	56	Skin	Cancer adjacent normal skin tissue	NAT				
M9	M	35	Skin	Normal skin tissue	Normal	Basal cell lamina of the epidermis and vessels partly positive	Basal cell lamina of the epidermis partly positive	Basal cell lamina of the epidermis partly positive	Epithelial cell nuclei partly positive
M10	M	47	Skin	Normal skin tissue	Normal				
M11	F	18	Skin	Normal skin tissue	Normal				
M12	M	25	Skin	Normal skin tissue	Normal				
M13	M	30	Skin	Normal skin tissue	Normal				
M14	M	43	Skin	Normal skin tissue	Normal				
M15	M	45	Skin	Normal skin tissue	Normal				
M16	F	21	Skin	Normal skin tissue	Normal				

*11a. GL803a: **Brain tumor** and adjacent tissue array*

position	sex	age	organ	pathology	grade	type	TGFß1	TGFß2 (Acris)	TGFß2 (SC)	p-SMAD 2/3
A1	F	14	Brain	Astrocytoma	1	Neoplastic	1	1	1	0
A2	F	15	Brain	Astrocytoma	1	Neoplastic	1	0	1	1
A3	M	41	Brain	Astrocytoma	2	Neoplastic	1	0	1	1
A4	F	34	Brain	Astrocytoma	2	Neoplastic	1	0	1	1
A5	M	73	Brain	Astrocytoma	2	Neoplastic	2	1	1	1
A6	M	36	Brain	Astrocytoma	2	Neoplastic	1	2	1	0
A7	M	51	Brain	Astrocytoma	2	Neoplastic	1	0	0	0
A8	F	16	Brain	Astrocytoma	2	Neoplastic	n.a.	n.a.	n.a.	n.a.
A9	M	25	Brain	Astrocytoma	2	Neoplastic	1	0	0	0
A10	M	33	Brain	Astrocytoma	2	Neoplastic	2	1	0	0
B1	M	62	Brain	Astrocytoma (tumoral necrosis)	-	Neoplastic	2	0	1	0
B2	F	39	Brain	Astrocytoma	1	Neoplastic	1	1	1	2
B3	M	53	Brain	Astrocytoma	3	Neoplastic	2	0	1	3
B4	M	57	Brain	Astrocytoma	3	Neoplastic	1	1	2	2
B5	M	52	Brain	Astrocytoma	3	Neoplastic	2	1	2	2
B6	M	41	Brain	Glioblastoma	4	Neoplastic	2	1	1	2
B7	M	59	Brain	Astrocytoma (sparse)	4	Neoplastic	1	1	1	2
B8	F	36	Brain	Astrocytoma	**3**--4	Neoplastic	1	1	1	2
B9	M	41	Brain	Astrocytoma	**3**--4	Neoplastic	2	0	2	1
B10	M	45	Brain	Glioblastoma	4	Neoplastic	3	0	1	1
C1	M	57	Brain	Glioblastoma	4	Neoplastic	2	1	1	1
C2	F	19	Brain	Glioblastoma	4	Neoplastic	2	1	2	1
C3	M	66	Brain	Glioblastoma	4	Neoplastic	1	0	1	1
C4	M	43	Brain	Glioblastoma	4	Neoplastic	3	0	2	1
C5	F	22	Brain	Glioblastoma	4	Neoplastic	0	n.a.	2	2
C6	M	49	Brain	Glioblastoma	4	Neoplastic	1	0	0	1
C7	F	64	Brain	Astrocytoma/**Glioblastoma**	3--**4**	Neoplastic	3	0	2	2
C8	M	60	Brain	Astrocytoma/**Glioblastoma**	3--**4**	Neoplastic	2	1	1	2

C9	F	43	Brain	Glioblastoma	4	Neoplastic	3	1	3	3
C10	F	27	Brain	Astrocytoma	**3**--4	Neoplastic	1	0	1	1
D1	F	66	Brain	Glioblastoma	4	Neoplastic	2	0	3	2
D2	F	50	Brain	Glioblastoma	4	Neoplastic	2	1	3	2
D3	F	31	Brain	Glioblastoma	4	Neoplastic	2	0	1	2
D4	F	52	Brain	Astrocytoma/**Glioblastoma**	3--**4**	Neoplastic	2	0	1	2
D5	M	58	Brain	Glioblastoma	4	Neoplastic	2	0	2	2
D6	F	41	Brain	Glioblastoma	4	Neoplastic	3	0	1	2
D7	M	24	Brain	Glioblastoma	4	Neoplastic	2	1	3	2
D8	F	42	Brain	Glioblastoma	4	Neoplastic	3	2	1	2
D9	F	59	Brain	Glioblastoma	4	Neoplastic	2	0	2	3
D10	M	71	Brain	Glioblastoma	4	Neoplastic	1	1	1	3
E1	F	37	Brain	Glioblastoma	4	Neoplastic	3	0	1	1
E2	F	10	Brain	Glioblastoma	4	Neoplastic	1	1	1	2
E3	F	44	Brain	Glioblastoma	4	Neoplastic	1	1	1	1
E4	M	43	Brain	Glioblastoma	4	Neoplastic	3	0	2	2
E5	F	6	Brain	Glioblastoma	4	Neoplastic	2	1	1	1
E6	M	64	Brain	Glioblastoma	4	Neoplastic	1	1	1	2
E7	M	25	Brain	Glioblastoma	4	Neoplastic	1	1	0	2
E8	M	43	Brain	Glioblastoma	4	Neoplastic	0	0	2	2
E9	M	20	Brain	Glioblastoma	4	Neoplastic	2	0	1	3
E10	F	68	Brain	Glioblastoma	4	Neoplastic	1	1	1	1
F1	M	30	Brain	Glioblastoma	4	Neoplastic	1	2	1	1
F2	M	35	Brain	Glioblastoma	4	Neoplastic	1	0	2	1
F3	M	42	Brain	Glioblastoma	4	Neoplastic	1	1	0	1
F4	M	43	Brain	Glioblastoma	4	Neoplastic	1	0	1	1
F5	M	55	Brain	Glioblastoma	4	Neoplastic	1	1	1	1
F6	M	46	Brain	Oligo-astrocytoma	-	Neoplastic	1	1	1	2
F7	M	45	Brain	Oligo-astrocytoma	-	Neoplastic	1	1	1	1
F8	F	48	Brain	Oligo-astrocytoma	-	Neoplastic	1	1	1	1
F9	F	50	Brain	Oligodendroglioma	?/**2**	Neoplastic	1	0	1	1
F10	F	46	Brain	Oligodendroglioma	2	Neoplastic	1	1	1	1
G1	M	41	Brain	Oligodendroglioma	2	Neoplastic	3	1	2	2
G2	M	29	Brain	Oligodendroglioma	2	Neoplastic	1	1	1	2
G3	F	30	Brain	Oligodendroglioma	2	Neoplastic	1	2	1	2
G4	M	39	Brain	Oligodendroglioma	2	Neoplastic	1	1	1	3
G5	M	40	Brain	Anaplastic oligodendroglioma	-	Neoplastic	1	0	1	1
G6	M	55	Brain	Anaplastic oligodendroglioma	-	Neoplastic	3	1	2	0
G7	M	43	Brain	Anaplastic oligodendroglioma	-	Neoplastic	1	1	2	2
G8	M	37	Brain	Malignant oligodendroglioma	3	Neoplastic	1	2	1	2
G9	F	14	Brain	Medulloblastoma	-	Neoplastic	0	1	1	2
G10	M	11	Brain	Medulloblastoma	-	Neoplastic	2	0	1	3
H1	M	3	Brain	Medulloblastoma	-	Neoplastic	1	1	2	1
H2	M	41	Brain	Ependymoma	-	Neoplastic	n.a.	0	1	1
H3	F	4	Brain	Ependymoma	-	Neoplastic	n.a.	1	2	1
H4	M	55	Brain	Malignant ependymoma	-	Neoplastic	n.a.	1	2	1
H5	M	41	Brain	Anaplastic ependymoma	-	Neoplastic	n.a.	0	1	2
H6	F	30	Brain	Cancer adjacent normal brain tissue	-	NAT	1	0	1	1
H7	F	49	Brain	Cancer adjacent normal brain tissue	-	NAT	1	1	2	1
H8	M	30	Brain	Cancer adjacent normal brain	-	NAT	1	0	2	1

				tissue						
H9	M	39	Brain	Cancer adjacent normal brain tissue	-	NAT	1	0	2	1
H10	M	52	Brain	Cancer adjacent normal brain tissue	-	NAT	2	1	2	2

Cases with equivocal designation by the supplier were reevaluated and the most likely grading by own examination is highlighted in bold type and underlined. Type was changed from "malignant" to "neoplastic" what appears more appropriate in the context of also low-grade brain tumors contained on the array.

11b. GL2083a: ***Brain tumor*** *tissue array*

position	sex	age	organ	pathology	grade	type	TGFß1	TGFß2 (Acris)	TGFß2 (SC)	p-SMAD 2/3
A1	F	52	Cerebrum	Astrocytoma	1	Neoplastic	1	0	1	1
A2	M	44	Cerebrum	Astrocytoma	1	Neoplastic	1	0	1	1
A3	M	36	Cerebrum	Astrocytoma	1	Neoplastic	0	1	2	1
A4	F	38	Cerebrum	Astrocytoma	1	Neoplastic	1	0	2	1
A5	M	48	Cerebrum	Astrocytoma	1	Neoplastic	0	0	2	0
A6	M	53	Cerebrum	Astrocytoma	1--**2**	Neoplastic	1	0	2	0
A7	F	41	Cerebrum	Astrocytoma	1--**2**	Neoplastic	0	0	2	1
A8	M	41	Cerebrum	Astrocytoma	**1**--2	Neoplastic	0	0	2	1
A9	M	50	Cerebrum	Astrocytoma	1	Neoplastic	0	0	1	0
A10	M	48	Cerebrum	Astrocytoma	1--**2**	Neoplastic	1	0	1	1
A11	F	44	Cerebrum	Astrocytoma	1	Neoplastic	1	0	1	1
A12	M	51	Cerebrum	Astrocytoma	1--**2**	Neoplastic	1	0	1	2
A13	M	37	Cerebrum	Astrocytoma	1	Neoplastic	1	0	2	2
A14	M	31	Cerebrum	Astrocytoma	1	Neoplastic	1	1	2	1
A15	M	38	Cerebrum	Astrocytoma	1	Neoplastic	1	1	2	2
A16	M	40	Cerebrum	Astrocytoma	1	Neoplastic	1	0	2	1
B1	F	53	Cerebrum	Astrocytoma	1	Neoplastic	1	0	0	1
B2	F	40	Cerebrum	Astrocytoma	1--**2**	Neoplastic	1	0	0	2
B3	F	54	Cerebrum	Astrocytoma	1	Neoplastic	1	0	0	1
B4	M	57	Cerebrum	Astrocytoma	1--**2**	Neoplastic	1	0	1	1
B5	F	40	Cerebrum	Astrocytoma	2	Neoplastic	1	0	0	1
B6	M	43	Cerebrum	Astrocytoma	1	Neoplastic	0	0	1	2
B7	F	44	Cerebrum	Astrocytoma	1	Neoplastic	1	0	1	1
B8	M	40	Cerebrum	Astrocytoma	1	Neoplastic	1	1	1	3
B9	M	37	Cerebrum	Astrocytoma	2	Neoplastic	1	0	0	2
B10	F	40	Cerebrum	Astrocytoma	2	Neoplastic	1	0	0	1
B11	M	41	Cerebrum	Astrocytoma	2	Neoplastic	0	0	1	2
B12	F	36	Cerebrum	Astrocytoma	2	Neoplastic	1	0	1	2
B13	M	47	Cerebrum	Astrocytoma	1--**2**	Neoplastic	0	1	1	2
B14	M	42	Cerebrum	Astrocytoma	1--**2**	Neoplastic	1	1	2	2
B15	F	28	Cerebrum	Astrocytoma	1	Neoplastic	0	1	1	1
B16	M	60	Cerebrum	Astrocytoma	2	Neoplastic	1	0	1	1
C1	M	38	Cerebrum	Astrocytoma	1--**2**	Neoplastic	1	0	0	1
C2	M	30	Cerebrum	Astrocytoma	1	Neoplastic	0	0	0	1
C3	F	59	Cerebrum	Astrocytoma	2	Neoplastic	1	1	1	1
C4	F	42	Cerebrum	Astrocytoma	2	Neoplastic	1	0	1	1
C5	M	33	Cerebrum	Astrocytoma	2	Neoplastic	1	0	2	1
C6	M	48	Cerebrum	Astrocytoma	2	Neoplastic	1	0	2	2

C7	M	51	Cerebrum	Astrocytoma	2	Neoplastic	0	0	1	1
C8	M	36	Cerebrum	Astrocytoma	2	Neoplastic	0	0	0	2
C9	M	47	Cerebrum	Astrocytoma	2	Neoplastic	0	0	0	2
C10	M	47	Cerebrum	Astrocytoma	2	Neoplastic	1	0	0	2
C11	M	56	Cerebrum	Astrocytoma	2	Neoplastic	1	0	1	2
C12	F	34	Cerebrum	Astrocytoma	2	Neoplastic	1	0	1	2
C13	F	51	Cerebrum	Astrocytoma	**1**--2	Neoplastic	1	0	1	1
C14	M	41	Cerebrum	Astrocytoma	2	Neoplastic	1	1	1	1
C15	M	38	Cerebrum	Astrocytoma	1	Neoplastic	1	0	1	1
C16	M	52	Cerebrum	Astrocytoma	1	Neoplastic	0	1	1	1
D1	F	41	Cerebrum	Astrocytoma	2	Neoplastic	0	1	0	1
D2	F	30	Cerebrum	Astrocytoma	1	Neoplastic	0	0	1	1
D3	M	47	Cerebrum	Astrocytoma	2	Neoplastic	1	1	2	0
D4	F	45	Cerebrum	Astrocytoma	2	Neoplastic	1	0	2	2
D5	F	39	Cerebrum	Astrocytoma	2	Neoplastic	0	0	1	2
D6	M	23	Cerebrum	Astrocytoma	2	Neoplastic	0	0	1	2
D7	M	63	Cerebrum	Astrocytoma	2	Neoplastic	0	0	0	1
D8	M	49	Cerebrum	Astrocytoma	1--**2**	Neoplastic	0	0	0	1
D9	F	52	Cerebrum	Astrocytoma **(infiltrated Cortex)**	**2**/-	**(Non-)** Neoplastic	1	1	1	1
D10	F	40	Cerebrum	Astrocytoma	1--**2**	Neoplastic	1	1	1	1
D11	M	53	Cerebrum	Astrocytoma	2	Neoplastic	1	0	0	2
D12	M	51	Cerebrum	Astrocytoma	2	Neoplastic	0	1	0	2
D13	F	55	Cerebrum	Astrocytoma	2	Neoplastic	2	1	1	2
D14	M	39	Cerebrum	Astrocytoma	1	Neoplastic	1	1	2	1
D15	F	37	Cerebrum	Astrocytoma (brain tissue sparse)	-	Neoplastic	2	0	3	1
D16	M	42	Cerebrum	Astrocytoma	2	Neoplastic	1	1	1	3
E1	F	35	Cerebrum	Astrocytoma	1--**2**	Neoplastic	1	0	0	0
E2	F	47	Cerebrum	Astrocytoma	2	Neoplastic	0	2	0	1
E3	F	47	Cerebrum	Astrocytoma	2	Neoplastic	0	0	1	0
E4	M	51	Cerebrum	Astrocytoma	2	Neoplastic	1	0	1	1
E5	F	36	Cerebrum	Astrocytoma	1	Neoplastic	1	0	0	2
E6	M	49	Cerebrum	Astrocytoma (brain tissue)	-	Neoplastic	0	1	1	2
E7	F	44	Cerebrum	Astrocytoma	2	Neoplastic	1	0	1	1
E8	M	39	Cerebrum	Astrocytoma	2	Neoplastic	1	2	3	2
E9	M	53	Cerebrum	Astrocytoma	2	Neoplastic	1	1	1	2
E10	M	37	Cerebrum	Astrocytoma	1--**2**	Neoplastic	1	1	2	1
E11	F	45	Cerebrum	Astrocytoma	2	Neoplastic	1	0	1	2
E12	M	35	Cerebrum	Astrocytoma	2	Neoplastic	3	0	1	2
E13	M	50	Cerebrum	Astrocytoma	2	Neoplastic	1	0	2	1
E14	M	62	Cerebrum	Astrocytoma	2	Neoplastic	1	1	2	2
E15	M	43	Cerebrum	Astrocytoma	1--**2**	Neoplastic	1	0	1	2
E16	M	45	Cerebrum	Astrocytoma	2	Neoplastic	0	1	1	2
F1	M	51	Cerebrum	Astrocytoma	1--**2**	Neoplastic	1	1	0	0
F2	M	37	Cerebrum	Astrocytoma	2	Neoplastic	1	1	0	1
F3	F	52	Cerebrum	Astrocytoma	2	Neoplastic	1	2	1	0
F4	F	48	Cerebrum	Astrocytoma	2	Neoplastic	0	0	1	0
F5	F	46	Cerebrum	Astrocytoma	2	Neoplastic	1	0	1	1
F6	M	36	Cerebrum	Astrocytoma	2	Neoplastic	0	1	1	1
F7	M	52	Cerebrum	Astrocytoma	2	Neoplastic	1	0	2	1
F8	M	28	Cerebrum	Astrocytoma	1--**2**	Neoplastic	1	1	3	2
F9	F	51	Cerebrum	Astrocytoma	2	Neoplastic	0	0	1	2

F10	M	44	Cerebrum	Astrocytoma	2	Neoplastic	0	1	1	2
F11	F	48	Cerebrum	Astrocytoma	2	Neoplastic	1	0	1	3
F12	M	58	Cerebrum	Astrocytoma	2	Neoplastic	0	0	1	2
F13	M	60	Cerebrum	Astrocytoma	2	Neoplastic	1	1	2	2
F14	M	48	Cerebrum	Astrocytoma	2	Neoplastic	1	0	2	2
F15	M	40	Cerebrum	Astrocytoma	2	Neoplastic	1	1	1	3
F16	M	43	Cerebrum	Astrocytoma	2	Neoplastic	1	0	1	2
G1	M	37	Cerebrum	Astrocytoma	2	Neoplastic	1	1	0	1
G2	F	44	Cerebrum	Astrocytoma	2	Neoplastic	0	0	1	1
G3	M	51	Cerebrum	Astrocytoma	3	Neoplastic	0	0	1	1
G4	F	41	Cerebrum	Astrocytoma	2	Neoplastic	1	0	1	1
G5	F	48	Cerebrum	Astrocytoma	2	Neoplastic	0	1	2	1
G6	F	41	Cerebrum	Astrocytoma	2	Neoplastic	1	1	1	3
G7	F	51	Cerebrum	Astrocytoma	2	Neoplastic	1	0	2	2
G8	M	51	Cerebrum	Astrocytoma	2	Neoplastic	1	1	2	1
G9	M	56	Cerebrum	Astrocytoma	2	Neoplastic	0	0	1	2
G10	M	52	Cerebrum	Astrocytoma	2	Neoplastic	1	0	1	2
G11	F	52	Cerebrum	Astrocytoma	2	Neoplastic	1	0	2	3
G12	M	51	Cerebrum	Astrocytoma	2	Neoplastic	1	0	2	0
G13	F	49	Cerebrum	Astrocytoma	2	Neoplastic	1	0	2	2
G14	M	37	Cerebrum	Astrocytoma	3	Neoplastic	1	1	3	1
G15	M	44	Cerebrum	Astrocytoma	2	Neoplastic	1	0	2	2
G16	M	34	Cerebrum	Astrocytoma	3	Neoplastic	2	0	2	2
H1	M	36	Cerebrum	Astrocytoma	3	Neoplastic	1	0	0	1
H2	M	46	Cerebrum	Astrocytoma	3	Neoplastic	0	0	0	1
H3	F	40	Cerebrum	Astrocytoma	3	Neoplastic	0	0	0	1
H4	F	36	Cerebrum	Astrocytoma	3	Neoplastic	0	1	1	2
H5	F	64	Cerebrum	Astrocytoma	3	Neoplastic	0	0	1	2
H6	M	49	Cerebrum	Astrocytoma	3	Neoplastic	0	1	1	2
H7	M	48	Cerebrum	Astrocytoma	3	Neoplastic	1	0	1	2
H8	F	51	Cerebrum	Astrocytoma	3	Neoplastic	2	0	1	0
H9	M	54	Cerebrum	Astrocytoma	3	Neoplastic	1	1	2	2
H10	M	30	Cerebrum	Astrocytoma	3	Neoplastic	1	1	2	1
H11	F	32	Cerebrum	Astrocytoma	3	Neoplastic	1	1	3	1
H12	M	52	Cerebrum	Astrocytoma	3	Neoplastic	0	0	2	1
H13	M	70	Cerebrum	Astrocytoma (brain tissue)	-	Neoplastic	1	1	3	1
H14	M	51	Cerebrum	Astrocytoma	3	Neoplastic	0	0	2	2
H15	F	51	Cerebrum	Astrocytoma	3	Neoplastic	2	0	3	3
H16	M	56	Cerebrum	Astrocytoma	3	Neoplastic	2	0	3	2
I1	F	66	Cerebrum	Astrocytoma	3	Neoplastic	1	0	1	1
I2	M	63	Cerebrum	Astrocytoma	3	Neoplastic	1	0	0	0
I3	F	33	Cerebrum	Glioblastoma	4	Neoplastic	1	0	1	1
I4	M	58	Cerebrum	Glioblastoma	4	Neoplastic	1	0	1	2
I5	F	58	Cerebrum	Glioblastoma	4	Neoplastic	1	0	1	2
I6	M	40	Cerebrum	Glioblastoma	4	Neoplastic	1	1	2	1
I7	F	70	Cerebrum	Glioblastoma	4	Neoplastic	2	1	1	1
I8	F	27	Cerebrum	Giant cell glioblastoma	4	Neoplastic	1	0	2	2
I9	M	25	Cerebrum	Glioblastoma	4	Neoplastic	1	1	2	2
I10	F	65	Cerebrum	Glioblastoma	4	Neoplastic	1	1	3	2
I11	M	39	Cerebrum	Glioblastoma	4	Neoplastic	1	0	2	2
I12	M	29	Cerebrum	Glioblastoma	4	Neoplastic	1	1	2	2
I13	F	68	Cerebrum	Glioblastoma	4	Neoplastic	1	0	2	1

I14	M	19	Cerebrum	Glioblastoma	4	Neoplastic	1	1	3	2
I15	F	46	Cerebrum	Glioblastoma	4	Neoplastic	1	1	2	1
I16	M	51	Cerebrum	Giant cell glioblastoma	4	Neoplastic	0	1	3	1
J1	F	54	Cerebrum	Glioblastoma	4	Neoplastic	1	1	0	1
J2	M	44	Cerebrum	Glioblastoma	4	Neoplastic	1	0	0	1
J3	M	9	Cerebrum	Glioblastoma	4	Neoplastic	1	1	0	1
J4	M	34	Cerebrum	Glioblastoma	4	Neoplastic	1	0	0	0
J5	M	49	Cerebrum	Astrocytoma (sparse)	-	Neoplastic	3	0	1	1
J6	F	42	Cerebrum	Glioblastoma	4	Neoplastic	0	1	1	2
J7	M	52	Cerebrum	Glioblastoma	4	Neoplastic	1	0	1	2
J8	M	61	Cerebrum	Glioblastoma	4	Neoplastic	1	0	2	2
J9	M	55	Cerebrum	Glioblastoma	4	Neoplastic	1	0	3	2
J10	F	60	Cerebrum	Astrocytoma	**1**--2	Neoplastic	0	1	2	1
J11	F	61	Cerebrum	Glioblastoma	4	Neoplastic	1	1	1	1
J12	F	55	Cerebrum	Glioblastoma	4	Neoplastic	0	1	1	1
J13	M	26	Cerebrum	Glioblastoma	4	Neoplastic	2	0	1	2
J14	M	49	Cerebrum	Glioblastoma	4	Neoplastic	1	1	2	3
J15	F	10	Cerebrum	Glioblastoma	4	Neoplastic	1	1	2	3
J16	F	46	Cerebrum	Glioblastoma	4	Neoplastic	1	0	2	1
K1	F	49	Cerebrum	Glioblastoma	4	Neoplastic	1	0	0	1
K2	M	51	Cerebrum	Glioblastoma	4	Neoplastic	1	0	0	1
K3	M	45	Cerebrum	Glioblastoma	4	Neoplastic	1	0	0	0
K4	M	37	Cerebrum	Oligo-astrocytoma	2	Neoplastic	1	1	1	2
K5	M	52	Cerebrum	Oligo-astrocytoma	2	Neoplastic	0	0	0	1
K6	M	50	Cerebrum	Oligo-astrocytoma	2	Neoplastic	1	0	0	2
K7	M	46	Cerebrum	Oligo-astrocytoma	2	Neoplastic	0	2	1	2
K8	F	55	Cerebrum	Oligo-astrocytoma	2	Neoplastic	1	2	1	2
K9	F	50	Cerebrum	Oligo-astrocytoma	2	Neoplastic	1	1	1	2
K10	F	48	Cerebrum	Oligo-astrocytoma	2	Neoplastic	1	0	1	2
K11	M	33	Cerebrum	Oligodendroglioma	2	Neoplastic	0	1	1	2
K12	M	39	Cerebrum	Oligodendroglioma	2	Neoplastic	0	0	1	3
K13	M	46	Cerebrum	Oligodendroglioma	2	Neoplastic	2	0	0	2
K14	M	73	Cerebrum	Oligodendroglioma	2	Neoplastic	1	1	0	3
K15	F	46	Cerebrum	Oligodendroglioma	2	Neoplastic	0	1	1	3
K16	F	46	Cerebrum	Oligodendroglioma	2	Neoplastic	1	1	2	2
L1	M	66	Cerebrum	Oligodendroglioma	2	Neoplastic	0	1	0	1
L2	F	30	Cerebrum	Oligodendroglioma	2	Neoplastic	1	0	0	2
L3	F	47	Cerebrum	Oligodendroglioma	2	Neoplastic	1	0	0	0
L4	F	41	Cerebrum	Ependymoma	2	Neoplastic	1	0	0	1
L5	F	55	Cerebrum	Ependymoma	2	Neoplastic	1	0	1	1
L6	M	41	Cerebrum	Ependymoma	2	Neoplastic	1	0	1	2
L7	M	72	Cerebrum	Anaplastic ependymoma	3	Neoplastic	0	0	1	2
L8	M	51	Cerebrum	Anaplastic ependymoma	3	Neoplastic	2	0	n.a.	0
L9	M	41	Cerebrum	Ependymoma	2	Neoplastic	1	1	1	1
L10	M	26	Cerebrum	Anaplastic ependymoma	3	Neoplastic	1	0	1	1
L11	M	48	Cerebrum	Anaplastic ependymoma	3	Neoplastic	1	0	1	1
L12	M	55	Cerebrum	Anaplastic ependymoma	3	Neoplastic	1	0	1	1
L13	F	29	Cerebrum	Anaplastic ependymoma	3	Neoplastic	1	1	0	0
L14	M	51	Cerebrum	Anaplastic ependymoma	3	Neoplastic	1	0	0	2
L15	M	44	Cerebrum	Ganglioglioma	-	Neoplastic	1	1	1	1
L16	F	50	Cerebrum	Gliosarcoma	-	Neoplastic	0	0	1	1
M1	M	52	Cerebrum	Cancer adjacent normal brain	-	NAT	2	0	0	1

				tissue						
M2	M	43	Cerebrum	Cancer adjacent normal brain tissue	-	NAT	1	0	0	2
M3	F	47	Cerebrum	Cancer adjacent normal brain tissue	-	NAT	1	0	0	2
M4	F	49	Cerebrum	Cancer adjacent normal brain tissue	-	NAT	1	0	1	2
M5	M	38	Cerebrum	Cancer adjacent normal brain tissue	-	NAT	1	0	1	2
M6	M	30	Cerebrum	Cancer adjacent normal brain tissue	-	NAT	2	0	2	1
M7	M	67	Cerebrum	Cancer adjacent normal brain tissue	-	NAT	1	1	2	1
M8	M	38	Cerebrum	Cancer adjacent normal brain tissue	-	NAT	1	1	1	2
M9	M	32	Cerebrum	Normal brain tissue	-	Normal	1	0	1	1
M10	F	50	Cerebrum	Normal brain tissue	-	Normal	1	1	1	1
M11	F	42	Cerebrum	Normal brain tissue	-	Normal	1	0	1	2
M12	F	38	Cerebrum	Normal brain tissue	-	Normal	1	1	1	1
M13	F	28	Cerebrum	Normal brain tissue	-	Normal	1	0	1	1
M14	F	24	Cerebrum	Normal brain tissue	-	Normal	1	0	1	2
M15	F	20	Cerebrum	Normal brain tissue	-	Normal	1	0	1	1
M16	M	49	Cerebrum	Normal brain tissue	-	Normal	2	1	1	1

Cases with equivocal designation by the supplier were reevaluated and the most likely grading by own examination is highlighted in bold type and underlined. Type was changed from "malignant" to "neoplastic" what appears more appropriate in the context of also low-grade brain tumors contained on the array.

*12a. LY2086: **Lymphoma** tumor tissue array*

position	sex	age	organ	pathology	type	TGFß1	TGFß2 (Acris)	TGFß2 (SC)	p-SMAD 2/3
A1	F	63	Lymph node	Diffuse B-cell lymphoma of right oxter	Malignant	2	0	0	0
A2	M	77	Lymph node	Diffuse B-cell lymphoma of right oxter	Malignant	1	0	1	1
A3	M	68	Lymph node	Diffuse large B-cell lymphoma of left groin	Malignant	1	1	2	2
A4	M	15	Lymph node	Nodular diffuse B-cell lymphoma of right medialis elbow joint	Malignant	n.a.	n.a.	n.a.	n.a.
A5	M	51	Lymph node	Diffuse B-cell lymphoma of right groin	Malignant	1	0	0	1
A6	M	28	Lymph node	Diffuse B-cell lymphoma	Malignant	2	0	2	2
A7	F	57	Lymph node	Diffuse B-cell lymphoma of right neck	Malignant	1	1	1	1
A8	F	29	Lymph node	Diffuse B-cell lymphoma of left oxter	Malignant	0	1	2	1
A9	F	78	Lymph node	Diffuse small B-cell lymphoma of left face	Malignant	0	n.a.	1	1
A10	F	56	Lymph node	Diffuse B-cell lymphoma of mesentery	Malignant	0	n.a.	1	0
A11	M	43	Lymph node	Nodular B-cell lymphoma of mesentery	Malignant	0	n.a.	1	0
A12	F	50	Lymph node	Diffuse B-cell lymphoma of stomach	Malignant	1	1	1	0
A13	M	31	Lymph node	Diffuse large B-cell lymphoma of left leg	Malignant	1	1	0	0

A14	M	67	Lymph node	Diffuse large B-cell lymphoma of right groin	Malignant	1	1	0	1
A15	F	39	Lymph node	Diffuse B-cell lymphoma of neck	Malignant	1	1	0	0
A16	F	50	Lymph node	Diffuse B-cell lymphoma of neck	Malignant	2	1	0	0
B1	M	45	Lymph node	Diffuse B-cell lymphoma of left groin	Malignant	3	0	1	0
B2	M	13	Lymph node	Diffuse B-cell lymphoma of right inferior belly	Malignant	1	1	1	2
B3	M	60	Lymph node	Diffuse B-cell lymphoma of right abdominal part	Malignant	1	1	1	3
B4	F	64	Lymph node	Diffuse large B-cell lymphoma of right armpit	Malignant	3	1	1	2
B5	M	52	Lymph node	Diffuse B-cell lymphoma of right groin	Malignant	n.a.	n.a.	n.a.	n.a.
B6	F	62	Lymph node	Diffuse large B-cell lymphoma of left armpit	Malignant	2	1	0	2
B7	F	75	Lymph node	Diffuse large B-cell lymphoma of left oxter	Malignant	1	0	n.a.	3
B8	F	50	Lymph node	Diffuse B-cell lymphoma of left pars palatalis	Malignant	1	1	1	3
B9	F	40	Lymph node	Large B-cell lymphoma of right oxter	Malignant	1	1	1	3
B10	M	10	Lymph node	Diffuse small B-cell lymphoma of left neck	Malignant	1	0	1	2
B11	M	30	Lymph node	Diffuse B-cell lymphoma of left neck	Malignant	1	1	1	2
B12	M	40	Lymph node	Diffuse B-cell lymphoma of mediastinum	Malignant	3	1	1	1
B13	F	76	Lymph node	Diffuse B-cell lymphoma	Malignant	1	n.a.	n.a.	3
B14	M	76	Lymph node	Large B-cell lymphoma of left abdominal part	Malignant	1	1	1	3
B15	M	52	Lymph node	Diffuse B-cell lymphoma over clavicle	Malignant	1	1	1	1
B16	F	36	Lymph node	Diffuse B-cell lymphoma of right breast	Malignant	1	1	0	0
C1	M	55	Lymph node	Diffuse B-cell lymphoma of oxter (sparse)	Malignant	2	1	1	1
C2	M	62	Lymph node	Diffuse B-cell lymphoma of right neck	Malignant	1	1	2	2
C3	F	68	Lymph node	Diffuse B-cell lymphoma of right groin	Malignant	1	1	1	2
C4	M	85	Lymph node	Diffuse B-cell lymphoma	Malignant	2	n.a.	2	3
C5	F	16	Lymph node	Diffuse B-cell lymphoma of neck	Malignant	3	1	2	2
C6	F	52	Lymph node	Diffuse B-cell lymphoma of right armpit	Malignant	2	1	0	2
C7	M	53	Lymph node	Diffuse B-cell lymphoma of spleen	Malignant	1	1	2	3
C8	M	59	Lymph node	Diffuse B-cell lymphoma of left thigh	Malignant	1	0	3	2
C9	M	8	Lymph node	Diffuse B-cell lymphoma of left oxter	Malignant	1	1	1	2
C10	M	50	Lymph node	Diffuse B-cell lymphoma of left oxter	Malignant	2	1	1	1
C11	M	51	Lymph node	Diffuse B-cell lymphoma of mediastinum	Malignant	3	0	0	1
C12	M	50	Lymph node	Diffuse large B-cell lymphoma of right groin	Malignant	3	1	1	1
C13	F	61	Lymph node	Diffuse B-cell lymphoma of right groin	Malignant	1	1	1	2

C14	M	40	Lymph node	Diffuse B-cell lymphoma of right armpit	Malignant	1	1	1	1
C15	M	67	Lymph node	Diffuse B-cell lymphoma	Malignant	1	1	1	2
C16	M	24	Lymph node	Diffuse B-cell lymphoma of neck	Malignant	3	0	1	1
D1	F	48	Lymph node	Diffuse B-cell lymphoma of left groin	Malignant	3	0	1	1
D2	M	52	Lymph node	Diffuse B-cell lymphoma of groin	Malignant	1	1	0	2
D3	F	51	Lymph node	Diffuse B-cell lymphoma of submaxilla	Malignant	0	1	1	2
D4	M	51	Lymph node	Diffuse B-cell lymphoma of right oxter	Malignant	1	1	3	1
D5	M	71	Lymph node	Diffuse B-cell lymphoma of neck	Malignant	2	1	1	1
D6	M	69	Lymph node	Diffuse B-cell lymphoma of right oxter	Malignant	1	0	1	2
D7	M	42	Lymph node	Diffuse B-cell lymphoma of left groin	Malignant	2	1	2	0
D8	F	41	Lymph node	Diffuse B-cell lymphoma of left oxter	Malignant	1	1	3	2
D9	M	38	Lymph node	Diffuse B-cell lymphoma of groin	Malignant	1	1	2	1
D10	M	65	Lymph node	Diffuse large B-cell lymphoma of oxter	Malignant	3	1	1	1
D11	M	36	Lymph node	Diffuse B-cell lymphoma of right armpit	Malignant	1	1	1	2
D12	F	43	Lymph node	Diffuse B-cell lymphoma of left groin	Malignant	1	1	1	1
D13	M	38	Lymph node	Diffuse B-cell lymphoma over clavicle	Malignant	2	0	2	2
D14	M	36	Lymph node	Diffuse B-cell lymphoma of left groin	Malignant	3	1	2	3
D15	M	56	Lymph node	Diffuse B-cell lymphoma	Malignant	1	1	1	2
D16	M	38	Lymph node	Diffuse B-cell lymphoma	Malignant	1	1	1	1
E1	M	53	Lymph node	Diffuse B-cell lymphoma of neck	Malignant	1	0	0	1
E2	M	40	Lymph node	Diffuse B-cell lymphoma of left groin	Malignant	2	0	2	1
E3	M	30	Lymph node	Diffuse B-cell lymphoma of left armpit	Malignant	2	0	2	2
E4	F	48	Lymph node	Diffuse large B-cell lymphoma of left groin	Malignant	1	0	1	3
E5	F	44	Lymph node	Diffuse large B-cell lymphoma of left groin	Malignant	1	0	2	2
E6	M	40	Lymph node	Diffuse B-cell lymphoma of left neck	Malignant	2	1	2	2
E7	M	39	Lymph node	Diffuse B-cell lymphoma of right groin	Malignant	2	1	2	1
E8	M	36	Lymph node	Diffuse large B-cell lymphoma of left groin	Malignant	3	1	1	1
E9	F	42	Lymph node	Diffuse B-cell lymphoma of right lower limb	Malignant	3	0	1	3
E10	F	36	Lymph node	Diffuse large B-cell lymphoma of left groin	Malignant	n.a.	1	n.a.	n.a.
E11	F	40	Lymph node	Diffuse B-cell lymphoma of right armpit	Malignant	1	1	2	3
E12	M	58	Lymph node	Diffuse B-cell lymphoma of neck	Malignant	2	1	2	1
E13	M	62	Lymph node	Diffuse large B-cell lymphoma of right groin	Malignant	1	1	1	2

E14	F	68	Lymph node	Diffuse B-cell lymphoma of submaxilla	Malignant	2	2	1	2
E15	M	66	Lymph node	Diffuse B-cell lymphoma of right neck	Malignant	1	1	1	2
E16	F	47	Lymph node	Diffuse B-cell lymphoma of right submaxilla	Malignant	1	n.a.	n.a.	1
F1	M	47	Lymph node	Diffuse B-cell lymphoma of right neck	Malignant	n.a.	n.a.	n.a.	n.a.
F2	M	47	Lymph node	Diffuse B-cell lymphoma of right neck	Malignant	1	0	2	2
F3	M	68	Lymph node	Diffuse B-cell lymphoma of right groin	Malignant	1	0	2	2
F4	F	58	Lymph node	Diffuse B-cell lymphoma over right clavicle	Malignant	2	1	0	3
F5	M	32	Lymph node	Diffuse B-cell lymphoma of left groin	Malignant	0	1	0	1
F6	M	44	Lymph node	Diffuse B-cell lymphoma of right groin	Malignant	1	1	1	1
F7	M	41	Lymph node	Diffuse B-cell lymphoma of left neck	Malignant	1	1	0	1
F8	M	39	Lymph node	Large B-cell lymphoma of right groin	Malignant	2	0	1	1
F9	F	48	Lymph node	Diffuse B-cell lymphoma of right groin	Malignant	3	n.a.	1	1
F10	M	71	Lymph node	Diffuse B-cell lymphoma of abdominal part	Malignant	1	1	0	2
F11	M	27	Lymph node	Diffuse B-cell lymphoma of neck	Malignant	1	1	1	3
F12	M	51	Lymph node	Diffuse B-cell lymphoma of right neck	Malignant	2	0	2	1
F13	F	54	Lymph node	Diffuse B-cell lymphoma of oxter	Malignant	3	1	2	1
F14	F	40	Lymph node	Diffuse B-cell lymphoma of right lower limb	Malignant	3	1	1	2
F15	F	48	Lymph node	Diffuse large B-cell lymphoma of right oxter	Malignant	2	1	2	3
F16	F	45	Lymph node	Diffuse B-cell lymphoma of left groin	Malignant	1	1	1	2
G1	F	70	Lymph node	Diffuse B-cell lymphoma of right neck	Malignant	3	0	3	2
G2	M	33	Lymph node	Diffuse B-cell lymphoma over left clavicle	Malignant	3	0	2	1
G3	F	40	Lymph node	Diffuse large B-cell lymphoma of left groin	Malignant	2	0	2	1
G4	F	43	Lymph node	Diffuse B-cell lymphoma of small intestine	Malignant	3	1	1	2
G5	M	3	Lymph node	Diffuse B-cell lymphoma of left neck	Malignant	2	1	1	1
G6	M	57	Lymph node	Diffuse B-cell lymphoma of right groin	Malignant	1	1	2	2
G7	M	72	Lymph node	Diffuse B-cell lymphoma of neck	Malignant	1	0	2	3
G8	F	64	Lymph node	Diffuse large B-cell lymphoma of left groin	Malignant	1	1	2	3
G9	M	65	Lymph node	Diffuse large B-cell lymphoma of groin	Malignant	n.a.	n.a.	n.a.	n.a.
G10	M	55	Lymph node	Diffuse large B-cell lymphoma of left neck	Malignant	2	1	1	3
G11	M	56	Lymph node	Nodular B-cell lymphoma of left submaxilla	Malignant	2	1	2	3
G12	F	65	Lymph node	Diffuse B-cell lymphoma of neck	Malignant	1	1	2	3
G13	F	44	Lymph node	Diffuse large B-cell lymphoma of left neck	Malignant	3	1	1	2

G14	M	74	Lymph node	Diffuse B-cell lymphoma of left popliteal fossa	Malignant	2	1	1	1
G15	M	54	Lymph node	Diffuse B-cell lymphoma of right armpit	Malignant	3	1	2	2
G16	M	62	Lymph node	Diffuse B-cell lymphoma of left groin	Malignant	1	1	1	1
H1	F	52	Lymph node	Diffuse B-cell lymphoma of left neck	Malignant	3	0	1	1
H2	M	63	Lymph node	Diffuse B-cell lymphoma of left armpit	Malignant	1	0	1	1
H3	F	50	Lymph node	Diffuse B-cell lymphoma	Malignant	1	0	1	2
H4	M	72	Lymph node	Diffuse B-cell lymphoma of groin	Malignant	1	1	1	2
H5	M	39	Lymph node	Diffuse B-cell lymphoma of right armpit	Malignant	1	1	1	3
H6	M	30	Lymph node	Diffuse B-cell lymphoma of left neck	Malignant	3	1	2	1
H7	F	41	Lymph node	Diffuse B-cell lymphoma of neck	Malignant	3	1	2	0
H8	F	36	Lymph node	Diffuse B-cell lymphoma of groin	Malignant	3	1	1	1
H9	M	67	Lymph node	Diffuse small B-cell lymphoma of right armpit	Malignant	2	0	n.a.	2
H10	F	51	Lymph node	Diffuse B-cell lymphoma of left thyroid gland	Malignant	1	2	1	1
H11	M	48	Lymph node	Diffuse B-cell lymphoma	Malignant	n.a.	n.a.	n.a.	n.a.
H12	M	80	Lymph node	Diffuse B-cell lymphoma of left neck	Malignant	1	1	1	3
H13	M	67	Lymph node	B-cell lymphoma of right armpit	Malignant	2	1	1	1
H14	M	45	Lymph node	Diffuse large B-cell lymphoma of left groin	Malignant	1	1	1	1
H15	F	43	Retroperit oneum	Diffuse B-cell lymphoma of retroperitoneum	Malignant	2	0	2	0
H16	F	35	Retroperit oneum	Diffuse B-cell lymphoma of retroperitoneum	Malignant	3	0	2	0
I1	M	3	Retroperit oneum	Diffuse B-cell lymphoma of retroperitoneum	Malignant	1	0	1	0
I2	M	42	Retroperit oneum	Diffuse large B-cell lymphoma of retroperitoneum	Malignant	2	0	1	3
I3	M	50	Retroperit oneum	Diffuse B-cell lymphoma of retroperitoneum	Malignant	2	0	1	2
I4	F	38	Retroperit oneum	Diffuse B-cell lymphoma of retroperitoneum	Malignant	2	0	1	2
I5	F	38	Lymph node	Burkitt-like lymphoma of left pelvic cavity	Malignant	2	1	1	3
I6	F	50	Lymph node	Burkitt-like lymphoma of left pelvic cavity	Malignant	1	1	1	1
I7	M	71	Lymph node	Burkitt-like lymphoma of submaxilla	Malignant	1	1	2	3
I8	M	66	Lymph node	Mantle cell lymphoma of right oxter	Malignant	1	n.a.	0	2
I9	M	52	Lymph node	T-cell-rich B-cell lymphoma of left neck	Malignant	2	1	2	2
I10	M	55	Lymph node	T-cell lymphoma of right oxter	Malignant	2	1	1	2
I11	M	52	Lymph node	T-cell lymphoma over left clavicle	Malignant	2	1	1	2
I12	F	44	Lymph node	T-cell lymphoma of left neck	Malignant	1	1	1	1
I13	M	68	Lymph node	T-cell lymphoma of right groin	Malignant	1	1	2	3

I14	M	76	Lymph node	T-cell lymphoma of groin	Malignant	2	0	2	2
I15	F	65	Lymph node	T-cell lymphoma of occiput	Malignant	1	1	2	1
I16	F	40	Lymph node	T-cell lymphoma of right lower limb	Malignant	3	1	2	2
J1	M	38	Lymph node	T-cell lymphoma of left groin	Malignant	1	0	1	0
J2	F	63	Lymph node	T-cell lymphoma of right inferior belly	Malignant	2	0	2	1
J3	F	36	Lymph node	T-cell lymphoma of left groin	Malignant	1	0	1	1
J4	M	57	Lymph node	T-cell lymphoma of neck	Malignant	1	1	2	2
J5	F	60	Lymph node	T-cell lymphoma of left neck	Malignant	2	1	2	2
J6	M	17	Lymph node	T-cell lymphoma over left clavicle	Malignant	2	2	1	1
J7	M	19	Lymph node	T-cell lymphoma of right neck (salivary gland tissue)	Malignant	2	1	3	2
J8	M	32	Lymph node	T-cell lymphoma	Malignant	1	1	2	2
J9	M	30	Lymph node	Lymphoepithelioid lymphoma of right armpit	Malignant	1	1	3	1
J10	M	64	Lymph node	Angioimmunoblastic T-cell lymphoma of right groin	Malignant	1	1	3	3
J11	F	40	Lymph node	T-cell lymphoma of right armpit	Malignant	1	1	2	3
J12	F	71	Lymph node	Diffuse T-cell lymphoma of left oxter	Malignant	1	1	3	3
J13	M	45	Lymph node	T-cell lymphoma of hibateral groin	Malignant	1	1	1	1
J14	M	41	Lymph node	T-cell lymphoma of groin	Malignant	1	1	1	2
J15	M	51	Lymph node	Angioimmunoblastic T-cell lymphoma of groin	Malignant	1	1	3	2
J16	M	45	Lymph node	Angioimmunoblastic T-cell lymphoma of right groin	Malignant	1	1	3	2
K1	F	42	Lymph node	Angioimmunoblastic T-cell lymphoma of neck	Malignant	1	0	0	0
K2	M	21	Lymph node	Follicular B-cell lymphoma of left oxter	Malignant	3	0	1	2
K3	F	38	Lymph node	Follicular B-cell lymphoma of right armpit	Malignant	1	1	1	3
K4	M	60	Lymph node	Follicular B-cell lymphoma of groin	Malignant	n.a.	n.a.	n.a.	2
K5	F	50	Lymph node	Follicular B-cell lymphoma of right neck	Malignant	2	0	2	1
K6	F	68	Lymph node	Follicular centrocytic lymphoma of left groin	Malignant	1	1	1	3
K7	M	50	Lymph node	Lymphoplasmacytoid lymphoma of anterior chest wall	Malignant	1	1	3	3
K8	M	63	Lymph node	Lymphoplasmacytoid lymphoma of left armpit	Malignant	2	1	2	2
K9	F	63	Lymph node	Lymphoplasmacytoid lymphoma of right abdominal part	Malignant	3	1	2	2
K10	M	31	Lymph node	Lymphoplasmacytoid lymphoma over left clavicle	Malignant	3	1	1	1
K11	F	40	Lymph node	Anaplastic large cell lymphoma of right groin	Malignant	3	1	2	2
K12	M	45	Lymph node	Anaplastic large cell lymphoma of oxter	Malignant	1	n.a.	3	3
K13	F	76	Lymph node	Anaplastic large cell lymphoma of left neck	Malignant	1	0	3	1

K14	M	64	Lymph node	Anaplastic large cell lymphoma of right groin	Malignant	1	1	3	3
K15	M	45	Lymph node	Anaplastic large cell lymphoma of left oxter	Malignant	2	1	3	2
K16	M	71	Lymph node	Diffuse anaplastic large cell lymphoma of right neck	Malignant	1	1	2	2
L1	F	23	Lymph node	Anaplastic large cell lymphoma of left groin	Malignant	1	n.a.	0	0
L2	F	69	Lymph node	Angioimmunoblastic T-cell lymphoma of right parotid gland	Malignant	1	1	1	2
L3	M	49	Lymph node	Angioimmunoblastic T-cell lymphoma of neck	Malignant	2	1	1	2
L4	F	48	Lymph node	Small lymphocytic lymphoma of neck	Malignant	2	0	2	3
L5	F	8	Lymph node	Nodular sclerosis Hodgkin's lymphoma of right submaxilla	Malignant	1	1	1	2
L6	M	4	Lymph node	Mixed cellularity Hodgkin's lymphoma of right neck	Malignant	1	0	1	1
L7	M	3	Lymph node	Lymphocytic predominance Hodgkin's lymphoma of right neck	Malignant	1	1	1	1
L8	M	24	Lymph node	Mixed cellularity Hodgkin's lymphoma of neck	Malignant	1	0	3	2
L9	F	73	Lymph node	Mixed cellularity Hodgkin's lymphoma of right neck	Malignant	1	1	2	1
L10	F	65	Lymph node	Nodular sclerosis Hodgkin's lymphoma of left groin	Malignant	3	0	3	1
L11	M	18	Lymph node	Mixed cellularity Hodgkin's lymphoma of right oxter	Malignant	1	1	1	1
L12	M	30	Lymph node	Lymphocytic depletion Hodgkin's lymphoma of right oxter	Malignant	1	0	2	1
L13	F	37	Lymph node	Mixed cellularity Hodgkin's lymphoma of neck	Malignant	2	1	2	1
L14	M	72	Lymph node	Lymphocytic depletion Hodgkin's lymphoma of left groin	Malignant	1	1	2	2
L15	M	27	Lymph node	Nodular sclerosis Hodgkin's lymphoma of neck	Malignant	1	1	2	1
L16	F	33	Lymph node	Lymphocytic predominance Hodgkin's lymphoma of left neck	Malignant	1	1	n.a.	1
M1	M	35	Lymph node	Normal lymph node tissue	Normal	0	n.a.	0	0
M2	M	35	Lymph node	Normal lymph node tissue	Normal	1	n.a.	1	0
M3	M	50	Lymph node	Normal lymph node tissue	Normal	3	n.a.	0	0
M4	F	21	Lymph node	Normal lymph node tissue	Normal	0	n.a.	1	0
M5	F	41	Lymph node	Normal lymph node tissue	Normal	2	n.a.	2	2
M6	M	30	Lymph node	Normal lymph node tissue	Normal	n.a.	n.a.	2	1
M7	F	18	Lymph node	Normal lymph node tissue	Normal	n.a.	n.a.	2	2
M8	M	35	Lymph node	Normal lymph node tissue	Normal	n.a.	n.a.	3	1
M9	M	30	Lymph node	Normal lymph node tissue (cataplasia)	Normal	n.a.	0	1	2
M10	M	32	Lymph node	Normal lymph node tissue (sparse)	Normal	n.a.	1	3	0
M11	M	40	Lymph node	Normal lymph node tissue	Normal	1	2	2	0
M12	M	25	Lymph node	Normal lymph node tissue	Normal	2	1	3	0
M13	M	28	Lymph node	Normal lymph node tissue	Normal	2	1	2	1

M14	F	15	Lymph node	Normal lymph node tissue	Normal	2	1	2	1
M15	M	16	Lymph node	Normal lymph node tissue	Normal	3	1	1	1
M16	M	45	Lymph node	Normal lymph node tissue	Normal	2	1	2	0

*12b. LM803: **Lymphoma** and normal lymph node tissue array*

position	sex	age	organ	pathology	type	TGFß1	TGFß2 (Acris)	TGFß2 (SC)	p-SMAD 2/3
A1	F	65	Lymph node	Nodular sclerosis Hodgkin lymphoma of left groin	Malignant	0	0	1	0
A2	F	65	Lymph node	Nodular sclerosis Hodgkin lymphoma of left groin	Malignant	0	0	1	1
A3	M	27	Lymph node	Nodular sclerosis Hodgkin lymphoma of left groin	Malignant	1	0	0	1
A4	M	27	Lymph node	Nodular sclerosis Hodgkin lymphoma of left groin	Malignant	n.a.	0	0	1
A5	M	17	Lymph node	Nodular sclerosis Hodgkin lymphoma of right neck	Malignant	n.a.	1	2	2
A6	M	17	Lymph node	Nodular sclerosis Hodgkin lymphoma of right neck	Malignant	n.a.	1	2	2
A7	M	68	Lymph node	Nodular sclerosis Hodgkin lymphoma of parotid gland	Malignant	n.a.	1	1	1
A8	M	68	Lymph node	Nodular sclerosis Hodgkin lymphoma of parotid gland (sparse)	Malignant	n.a.	1	1	1
A9	F	8	Lymph node	Nodular sclerosis Hodgkin lymphoma under submaxilla	Malignant	n.a.	0	0	1
A10	F	8	Lymph node	Nodular sclerosis Hodgkin lymphoma under submaxilla	Malignant	n.a.	1	0	0
B1	F	31	Lymph node	Mixed-cellularity Hodgkin lymphoma over clavicle	Malignant	1	0	0	1
B2	F	31	Lymph node	Mixed-cellularity Hodgkin lymphoma over clavicle	Malignant	1	1	1	1
B3	M	32	Lymph node	Mixed-cellularity Hodgkin lymphoma of neck	Malignant	0	1	0	1
B4	M	32	Lymph node	Mixed-cellularity Hodgkin lymphoma of neck	Malignant	1	1	0	1
B5	M	18	Lymph node	Mixed-cellularity Hodgkin lymphoma of right oxter	Malignant	1	0	1	1
B6	M	18	Lymph node	Mixed-cellularity Hodgkin lymphoma of right oxter	Malignant	n.a.	0	0	1
B7	M	50	Lymph node	Mixed-cellularity Hodgkin lymphoma of right armpit	Malignant	n.a.	1	1	1
B8	M	50	Lymph node	Mixed-cellularity Hodgkin lymphoma of right armpit	Malignant	n.a.	0	1	1
B9	F	48	Lymph node	Mixed-cellularity Hodgkin lymphoma of right groin	Malignant	2	1	2	2
B10	F	48	Lymph node	Mixed-cellularity Hodgkin lymphoma of right groin	Malignant	1	1	1	2
C1	F	42	Lymph node	Mixed-cellularity Hodgkin lymphoma of neck	Malignant	1	1	1	1
C2	F	42	Lymph node	Mixed-cellularity Hodgkin lymphoma of neck	Malignant	1	1	1	1
C3	F	41	Lymph node	Mixed-cellularity Hodgkin lymphoma of neck	Malignant	1	1	1	1
C4	F	41	Lymph node	Mixed-cellularity Hodgkin lymphoma of neck	Malignant	1	1	1	1
C5	M	17	Lymph node	Mixed-cellularity Hodgkin lymphoma of right neck	Malignant	1	1	0	0
C6	M	17	Lymph	Mixed-cellularity Hodgkin lymphoma of right	Malignant	1	1	0	0

			node	neck					
C7	M	4	Lymph node	Mixed-cellularity Hodgkin lymphoma of right neck	Malignant	0	1	1	1
C8	M	4	Lymph node	Mixed-cellularity Hodgkin lymphoma of right neck	Malignant	1	0	0	1
C9	F	73	Lymph node	Mixed-cellularity Hodgkin lymphoma of right neck	Malignant	1	1	1	1
C10	F	73	Lymph node	Mixed-cellularity Hodgkin lymphoma of right neck	Malignant	1	0	1	1
D1	M	26	Lymph node	Lymphocyte-predominant Hodgkin lymphoma of left armpit	Malignant	1	1	1	2
D2	M	26	Lymph node	Lymphocyte-predominant Hodgkin lymphoma of left armpit	Malignant	1	1	0	2
D3	M	45	Lymph node	Diffuse large B-Cell lymphoma of left forehead and temple (sparse)	Malignant	1	2	1	3
D4	M	45	Lymph node	Diffuse large B-Cell lymphoma of left forehead and temple	Malignant	1	2	1	3
D5	F	40	Lymph node	Diffuse large B-Cell lymphoma of left groin	Malignant	1	0	0	1
D6	F	40	Lymph node	Diffuse large B-Cell lymphoma of left groin	Malignant	1	0	0	1
D7	M	60	Lymph node	Diffuse large B-Cell lymphoma of left neck	Malignant	1	1	0	1
D8	M	60	Lymph node	Diffuse large B-Cell lymphoma of left neck	Malignant	1	1	1	1
D9	F	43	Lymph node	Diffuse large B-Cell lymphoma of left groin	Malignant	1	2	1	2
D10	F	43	Lymph node	Diffuse large B-Cell lymphoma of left groin	Malignant	1	2	1	2
E1	F	63	Lymph node	Diffuse B-cell lymphoma of neck	Malignant	2	1	3	2
E2	F	63	Lymph node	Diffuse B-cell lymphoma of neck	Malignant	2	1	3	2
E3	F	51	Lymph node	Diffuse B-cell lymphoma of colon	Malignant	2	1	3	2
E4	F	51	Lymph node	Diffuse B-cell lymphoma of colon	Malignant	1	1	3	2
E5	M	51	Lymph node	Diffuse B-cell lymphoma	Malignant	1	1	0	2
E6	M	51	Lymph node	Diffuse B-cell lymphoma	Malignant	1	0	0	2
E7	F	30	Lymph node	Follicular-diffuse B-cell lymphoma of right parotid region	Malignant	1	1	1	1
E8	F	30	Lymph node	Follicular-diffuse B-cell lymphoma of right parotid region	Malignant	2	1	1	1
E9	F	43	Lymph node	Follicular-diffuse B-cell lymphoma of abdominal part	Malignant	1	1	3	1
E10	M	43	Lymph node	Follicular-diffuse B-cell lymphoma of abdominal part	Malignant	2	1	2	1
F1	M	77	Lymph node	Follicular B-cell lymphoma of right neck	Malignant	2	1	3	1
F2	M	77	Lymph node	Follicular B-cell lymphoma of right neck	Malignant	1	1	3	2
F3	M	57	Lymph node	Diffuse B-cell lymphoma of left palate	Malignant	1	1	3	2
F4	M	57	Lymph node	Diffuse B-cell lymphoma of left palate	Malignant	1	1	3	2
F5	M	69	Lymph node	Follicular-diffuse B-cell lymphoma of ileum	Malignant	2	2	3	2
F6	M	69	Lymph node	Follicular-diffuse B-cell lymphoma of ileum	Malignant	1	2	3	2
F7	F	13	Lymph node	Diffuse T-cell lymphoma over clavicle	Malignant	1	2	0	2
F8	F	13	Lymph	Diffuse T-cell lymphoma over clavicle	Malignant	1	1	0	1

			node						
F9	F	62	Lymph node	Diffuse T-cell lymphoma of left oxter	Malignant	1	1	1	1
F10	F	62	Lymph node	Diffuse T-cell lymphoma of left oxter	Malignant	1	1	1	1
G1	F	28	Lymph node	Cancer adjacent normal lymph node tissue of breast (reactive hyperplasia)	NAT	1	1	1	1
G2	F	28	Lymph node	Cancer adjacent normal lymph node tissue of breast (reactive hyperplasia)	NAT	2	0	1	1
G3	F	50	Lymph node	Cancer adjacent normal lymph node tissue of oxter	NAT	2	1	1	2
G4	F	50	Lymph node	Cancer adjacent normal lymph node tissue of oxter	NAT	2	1	1	2
G5	F	27	Lymph node	Cancer adjacent normal lymph node tissue of breast (reactive hyperplasia)	NAT	3	1	1	2
G6	F	27	Lymph node	Cancer adjacent normal lymph node tissue of breast (reactive hyperplasia)	NAT	3	1	1	2
G7	F	37	Lymph node	Cancer adjacent normal lymph node tissue of armpit	NAT	2	1	1	1
G8	F	37	Lymph node	Cancer adjacent normal lymph node tissue of armpit	NAT	2	1	1	1
G9	F	68	Lymph node	Cancer adjacent normal lymph node tissue of breast (reactive hyperplasia)	NAT	2	1	0	1
G10	F	68	Lymph node	Cancer adjacent normal lymph node tissue of breast (reactive hyperplasia)	NAT	2	1	1	1
H1	M	38	Lymph node	Normal lymph node tissue	Normal	3	0	1	0
H2	M	38	Lymph node	Normal lymph node tissue	Normal	3	0	1	0
H3	M	35	Lymph node	Normal lymph node tissue	Normal	1	0	1	0
H4	M	35	Lymph node	Normal lymph node tissue	Normal	1	n.a.	1	0
H5	M	40	Lymph node	Normal lymph node tissue	Normal	1	n.a.	1	0
H6	M	40	Lymph node	Normal lymph node tissue	Normal	1	n.a.	1	0
H7	M	35	Lymph node	Normal lymph node tissue	Normal	2	1	2	1
H8	M	35	Lymph node	Normal lymph node tissue	Normal	0	n.a.	2	1
H9	F	15	Lymph node	Normal lymph node tissue	Normal	1	1	1	0
H10	F	15	Lymph node	Normal lymph node tissue	Normal	1	1	1	0

*13.BM483: Tumor tissue array (**Myeloma**)*

position	sex	age	organ	pathology	type	TGFß1	TGFß2 (Acris)	TGFß2 (SC)	p-SMAD 2/3
A1	F	44	Bone	Myeloma of right fourth rib	Malignant	2	0	n.d.	1
A2	F	44	Bone	Myeloma of right fourth rib	Malignant	0	0	n.d.	1
A3	M	69	Bone	Myeloma of left clavicle	Malignant	3	0	n.d.	3
A4	M	69	Bone	Myeloma of left clavicle	Malignant	3	0	n.d.	3
A5	M	39	Bone	Myeloma of eighth thoracic vertebrae	Malignant	1	1	n.d.	2
A6	M	39	Bone	Myeloma of eighth thoracic vertebrae	Malignant	2	1	n.d.	2
A7	M	57	Bone	Myeloma of right pubis	Malignant	1	2	n.d.	1
A8	M	57	Bone	Myeloma of right pubis	Malignant	2	2	n.d.	1
B1	M	64	Bone	Myeloma of right clavicle	Malignant	1	0	n.d.	3

B2	M	64	Bone	Myeloma of right clavicle	Malignant	1	0	n.d.	3
B3	M	37	Bone	Myeloma of rib	Malignant	0	0	n.d.	2
B4	M	37	Bone	Myeloma of rib	Malignant	0	0	n.d.	2
B5	M	57	Bone	Myeloma of sternum	Malignant	0	0	n.d.	1
B6	M	57	Bone	Myeloma of sternum	Malignant	1	0	n.d.	2
B7	F	46	Bone marrow	Myeloma of third rib	Malignant	1	1	n.d.	3
B8	F	46	Bone marrow	Myeloma of third rib	Malignant	1	1	n.d.	3
C1	F	43	Bone	Myeloma of left clavicle (sparse)	Malignant	0	1	n.d.	2
C2	F	43	Bone	Myeloma of left clavicle	Malignant	0	1	n.d.	2
C3	F	56	Bone	Myeloma of chest wall	Malignant	1	1 (nuclear)	n.d.	3
C4	F	56	Bone	Myeloma of chest wall	Malignant	1	1	n.d.	2
C5	F	94	Bone	Primitive neuroectodermal tumor (PNET) of right hip bone	Malignant	0	2	n.d.	2
C6	F	94	Bone	Primitive neuroectodermal tumor (PNET) of right hip bone	Malignant	0	2	n.d.	3
C7	F	15	Bone	Ewing's sarcoma of pelvis	Malignant	3	1	n.d.	2
C8	F	15	Bone	Ewing's sarcoma of pelvis (skeletal muscle)	Malignant	1	1	n.d.	2
D1	M	62	Bone	Diffuse large B-cell lymphoma of right femur	Malignant	1	1	n.d.	1
D2	M	62	Bone	Diffuse large B-cell lymphoma of right femur	Malignant	1	1	n.d.	1
D3	M	41	Bone	Cancer adjacent normal bone tissue	NAT	Tu-0, BM -1	0	n.d.	3
D4	M	41	Bone	Cancer adjacent normal bone tissue	NAT	Tu-0, BM -1	0	n.d.	3
D5	M	75	Bone	Cancer adjacent normal bone marrow tissue	NAT	Tu-0, BM -1	0	n.d.	2
D6	M	75	Bone	Cancer adjacent normal bone tissue	NAT	Tu-0, BM -1	0	n.d.	2
D7	M	72	Bone	Cancer adjacent normal bone marrow tissue	NAT	Tu-1, BM-3	0	n.d.	2
D8	M	72	Bone	Cancer adjacent normal bone tissue	NAT	Tu-1, BM-3	0	n.d.	2
E1	F	68	Bone	Cancer adjacent normal bone marrow tissue	NAT	Tu-1, BM-3	0	n.d.	1
E2	F	68	Bone	Cancer adjacent normal bone marrow tissue	NAT	Tu-1, BM-3	0	n.d.	2
E3	M	63	Bone	Cancer adjacent normal bone tissue	NAT	Tu-0, BM-3	0	n.d.	3
E4	M	63	Bone	Cancer adjacent normal bone tissue	NAT	n.a.	n.a.	n.d.	n.a.
E5	F	66	Bone	Cancer adjacent normal bone tissue	NAT	Tu-1, BM-3	0	n.d.	2
E6	F	66	Bone	Cancer adjacent normal bone tissue	NAT	Tu-0, BM-3	0	n.d.	2
E7	M	58	Bone	Cancer adjacent normal bone tissue	NAT	Tu-1, BM-3	0	n.d.	2
E8	M	58	Bone	Cancer adjacent normal bone tissue	NAT	Tu-2, BM-2	0	n.d.	2
F1	F	50	Bone	Cancer adjacent normal bone tissue	NAT	Tu-0, BM-3	0	n.d.	2
F2	F	50	Bone	Cancer adjacent normal bone tissue	NAT	Tu-1, BM-3	0	n.d.	2
F3	F	43	Bone	Cancer adjacent normal bone tissue	NAT	Tu-0, BM-3	1	n.d.	2
F4	F	43	Bone	Cancer adjacent normal bone tissue	NAT	Tu-1, BM-3	0	n.d.	1
F5	M	62	Bone	Cancer adjacent normal bone tissue	NAT	Tu-1, BM-3	0	n.d.	3

F6	M	62	Bone	Cancer adjacent normal bone tissue	NAT	Tu-1, BM-3	0	n.d.	2
F7	F	68	Bone	Cancer adjacent normal bone marrow tissue	NAT	Tu-0, BM-3	0	n.d.	2
F8	F	68	Bone	Cancer adjacent normal bone tissue	NAT	Tu-0, BM-3	0	n.d.	1

TU = tumor, BM = bone marrow

14. *FDA808ci-1_FDA* **normal organ tissue** *array*

position	sex	age	organ	pathology	type	TGFß1	TGFß2 (Acris)	TGFß2 (SC)	p-SMAD 2/3
A1	F	2	Cerebrum	Normal cerebrum tissue	Normal	1	0	1	1
A2	F	50	Cerebrum	Normal cerebrum tissue	Normal	1	1	1	1
A3	M	32	Cerebrum	Normal cerebrum tissue	Normal	1	1	1	1
A4	F	24	Cerebellum	Normal cerebellum tissue	Normal	1	2	0	2
A5	M	40	Cerebellum	Normal cerebellum tissue	Normal	2	2	0	2
A6	M	32	Cerebellum	Normal cerebellum tissue	Normal	1	2	0	2
A7	F	14	Adrenal gland	Normal adrenal gland tissue	Normal	1	3	0	1
A8	M	27	Adrenal gland	Normal adrenal gland tissue	Normal	2	2	1	1
A9	F	21	Adrenal gland	Normal adrenal gland tissue	Normal	1	2	0	1
A10	-	-	Blk	Blank	Blank				
B1	F	38	Ovary	Cancer adjacent normal ovary tissue	NAT	1	1	2	2
B2	F	36	Ovary	Cancer adjacent normal ovary tissue	NAT	0	0	2	0
B3	F	35	Ovary	Cancer adjacent normal ovary tissue	NAT	1	1	1	2
B4	M	38	Pancreas	Normal pancreas tissue	Normal	1	2	1	1
B5	F	21	Pancreas	Normal pancreas tissue	Normal	2	2	2	2
B6	M	27	Pancreas	Normal pancreas tissue	Normal	3	2	2	1
B7	M	25	Lymph node	Normal lymph node tissue	Normal	1	0	3	2
B8	M	43	Lymph node	Normal lymph node tissue	Normal	2	0	1	2
B9	M	16	Lymph node	Normal lymph node tissue	Normal	3	0	1	1
B10	-	-	Blk	Blank	Blank				
C1	M	15	Hypophysis	Normal hypophysis tissue	Normal	1	1	1	1
C2	M	17	Hypophysis	Cancer adjacent normal hypophysis tissue	NAT	1	2	1	2
C3	M	56	Hypophysis	Cancer adjacent normal hypophysis tissue	NAT	2	2	2	1
C4	M	35	Testis	Normal testis tissue	Normal	1	0	1	2
C5	M	74	Testis	Cancer adjacent normal testis tissue	NAT	1	2	2	3
C6	M	65	Testis	Cancer adjacent normal testis tissue	NAT	1	2	2	3
C7	M	37	Thyroid	Normal thyroid gland tissue	Normal	2	1	1	1
C8	M	22	Thyroid	Normal thyroid gland tissue	Normal	3	2	1	1
C9	F	40	Thyroid	Normal thyroid gland tissue	Normal	2	2	1	1
C10	-	-	Blk	Blank	Blank				
D1	F	35	Breast	Cancer adjacent normal breast duct tissue	NAT	3	1	1	1
D2	F	35	Breast	Normal breast tissue	Normal	1	1	1	1
D3	F	40	Breast	Cancer adjacent normal breast duct tissue	NAT	1	1	1	1
D4	M	26	Spleen	Normal spleen tissue	Normal	3	0	1	1
D5	M	35	Spleen	Normal spleen tissue	Normal	3	1	2	2

D6	M	30	Spleen	Normal spleen tissue	Normal	1	1	2	1
D7	F	15	Tonsil	Normal tonsil tissue	Normal	3	1	3	2
D8	F	18	Tonsil	Normal tonsil tissue	Normal	3	1	3	2
D9	M	47	Tonsil	Normal tonsil tissue	Normal	3	1	2	1
D10	-	-	Blk	Blank	Blank				
E1	M	10 mon.	Thymus gland	Cancer adjacent normal thymus gland tissue	NAT	1	0	1	1
E2	F	15	Thymus gland	Normal thymus gland tissue	Normal	1	1	2	1
E3	M	16	Thymus gland	Normal thymus gland tissue	Normal	1	1	2	1
E4	M	30	Bone marrow	Normal myeloid tissue	Normal	1	0		2
E5	F	61	Bone marrow	Cancer adjacent normal myeloid tissue	NAT	3	1	2	2
E6	M	70	Bone marrow	Cancer adjacent normal myeloid tissue	NAT	2	0	2	2
E7	M	53	Lung	Cancer adjacent normal lung tissue	NAT	1	1	2	1
E8	M	47	Lung	Normal lung tissue	Normal	1	1	2	1
E9	F	21	Lung	Normal lung tissue	Normal	1	2	2	2
E10	F	21	Bladder	Normal bladder tissue	Normal	1	2	2	1
F1	M	56	Heart	Normal cardiac muscle tissue	Normal	1	2	1	1
F2	F	42	Heart	Normal cardiac muscle tissue	Normal	1	1	1	1
F3	F	35	Heart	Normal cardiac muscle tissue	Normal	1	1	2	1
F4	M	35	Esophagus	Normal esophagus tissue	Normal	1	1	3	1
F5	M	24	Esophagus	Normal esophagus tissue	Normal	3	0	2	1
F6	F	42	Esophagus	Normal esophagus tissue	Normal	1	0	2	1
F7	M	48	Stomach	Normal stomach tissue	Normal	3	2	3	2
F8	F	42	Stomach	Normal stomach tissue	Normal	1	1	2	2
F9	M	35	Stomach	Normal stomach tissue	Normal	3	2	2	3
F10	M	30	Bladder	Normal bladder tissue	Normal	1	1	2	1
G1	M	40	Intestine	Normal small intestine tissue	Normal	1	2	1	1
G2	M	25	Intestine	Normal small intestine tissue	Normal	1	1	2	1
G3	M	35	Intestine	Normal small intestine tissue	Normal	2	1	3	1
G4	M	62	Colon	Cancer adjacent normal colon tissue	NAT	2	2	2	1
G5	M	30	Colon	Normal colon tissue	Normal	1	2	2	3
G6	M	35	Colon	Normal colon tissue	Normal	2	2	2	3
G7	M	40	Liver	Normal liver tissue	Normal	3	2	3	2
G8	F	35	Liver	Normal liver tissue	Normal	1	2	3	2
G9	M	35	Liver	Normal liver tissue	Normal	3	3	3	2
G10	M	28	Bladder	Normal bladder tissue	Normal	2	2	2	2
H1	M	35	Salivary gland	Normal salivary gland tissue	Normal	1	3	0	0
H2	M	77	Salivary gland	Cancer adjacent normal salivary gland tissue	NAT	2	3	1	1
H3	M	35	Salivary gland	Normal salivary gland tissue	Normal	1	2	1	1
H4	M	50	Kidney	Normal kidney tissue	Normal	2	3	1	2
H5	M	38	Kidney	Normal kidney tissue	Normal	1	3	1	2
H6	M	47	Kidney	Normal kidney tissue	Normal	1	3	2	3
H7	M	35	Prostate	Cancer adjacent normal prostate tissue	NAT	1	1	1	0
H8	M	43	Prostate	Normal prostate tissue	Normal	1	1	1	1
H9	M	31	Prostate	Normal prostate tissue	Normal	1	2	1	1
H10	M	42	Adrenal gland	Pheochromocytoma (tissue marker)	Malignant	1	1	2	2

Suppl. Table 2. Evaluation of the staining of the tissue arrays. Reported are mean expression scores and standard deviations for the different antigens across each individual array. Also reported is the percentage of immunohistochemically positive tumors (score>0) as well as the percentage of relevantly positive tumors (score>1) on each array. For some arrays further comparisons (e.g. primary vs. metastatic tumors or tumors vs. non-neoplastic tissues) are reported and significance of expression differences was assessed.

1. BR10010: ***Breast cancer*** *and matched metastatic carcinoma tissue array*

This array comprised 100 cores corresponding to 50 breast cancer patients. Both primary tumors and matching metastatic lesions were included on the array.

BR10010: Breast cancer and matched metastatic carcinoma tissue array

	TGF-ß1	TGF-ß2(Acris)	TGF-ß2(SC)	p-Smad2/3
mean/median (primary tumors)	1.68/1.5	1.39/1	1.28/1	1.62/2
mean/median (metastasis)	1.60/2	1.68/2	1.64/2	1.90/2
standard deviation/IQR (primary)	0.83/1	0.72/1	0.81/1	0.75/1
standard deviation/IQR (metastasis)	0.61/1	0.86/1	0.75/1	0.79/0
f-test (primary versus metastasis)	0.03	0.27	0.59	0.75
paired (!) t-test (primary versus metastasis)	0.561	0.063	**0.009**	0.065

	TGF-ß1	TGF-ß2(Acris)	TGF-ß2(SC)	p-Smad2/3
no. tumors	94	88	100	100
no. tumors with score 0	1	7	10	5
no. tumors with score 1	44	37	43	30
no. tumors with score 2	37	34	38	49
no. tumors with score 3	12	10	9	16
no. tumors with score >0	93	81	90	95
% positive tumors	**99**	**92**	**90**	**95**
no tumors with score >1	49	44	47	65
% relevantly positive tumors	**52**	**50**	**47**	**65**

*2. OV2086: **Ovary cancer** survey tissue array*

This array included 208 cores of 104 patients. Different types of ovarian carcinoma tissues were represented, most of them corresponding to ovarian adenocarcinoma.

OV2086: Ovary cancer survey tissue array

	TGF-ß1	TGF-ß2(Acris)	TGF-ß2(SC)	p-Smad2/3
mean/median (malignant)	1.27/1	1.26/1	1.46/1	1.63/2
standard deviation/IQR (malignant)	0.60/0	0.69/1	0.88/1	0.90/1

	TGF-ß1	TGF-ß2(Acris)	TGF-ß2(SC)	p-Smad2/3
no. malignant tumors	200	172	199	201
no. malignant tumors with score 0	5	18	20	26
no. malignant tumors with score 1	148	98	100	55
no. malignant tumors with score 2	36	50	47	88
no. malignant tumors with score 3	11	6	32	32
no. malignant tumors with score >0	195	154	179	175
% positive malignant tumors	**98**	**90**	**90**	**87**
no malignant tumors with score >1	47	56	79	120
% relevantly positive tumors	**24**	**33**	**40**	**60**

3. PR8010: ***Prostate cancer*** *tissue array*

This array contained 80 cores from 80 patients, the broad majority of cases representing prostate adenocarcinoma.

PR8010: Prostate cancer tissue array

	TGF-ß1	TGF-ß2(Acris)	TGF-ß2(SC)	p-Smad2/3
mean/median (tumor)	1.11/1	0.83/1	1.10/1	1.61/2
standard deviation/IQR (tumor)	0.77/0	0.51/0	0.72/0	0.78/1

	TGF-ß1	TGF-ß2(Acris)	TGF-ß2(SC)	p-Smad2/3
no. tumors	70	66	77	77
no. tumors with score 0	12	15	13	4
no. tumors with score 1	43	47	46	32
no. tumors with score 2	10	4	15	31
no. tumors with score 3	5	0	3	10
no. tumors with score >0	58	51	64	73
% positive tumors	**83**	**77**	**83**	**95**
no tumors with score >1	15	4	18	41
% relevantly positive tumors	**21**	**6**	**23**	**53**

*4. LC20813: **Lung cancer** tissue array*

This array comprised 208 cores from 208 patients. Tumor types in their majority included lung adenocarcinoma, squamous cell carcinoma and small cell carcinoma as well as few sampes of less common lung carinoma subentities.

LC20813: Lung cancer tissue array

	TGF-ß1	TGF-ß2(Acris)	TGF-ß2(SC)	p-Smad2/3
mean/median (malignant)	1.44/1	1.25/1	1.34/1	1.53/2
standard deviation/IQR (malignant)	0.72/1	0.79/1	0.83/1	0.86/1

	TGF-ß1	TGF-ß2(Acris)	TGF-ß2(SC)	p-Smad2/3
no. malignant tumors	148	114	198	198
no. malignant tumors with score 0	4	17	33	28
no. malignant tumors with score 1	91	60	77	58
no. malignant tumors with score 2	37	29	75	91
no. malignant tumors with score 3	16	8	13	21
no. malignant tumors with score >0	144	97	165	170
% positive malignant tumors	**97**	**85**	**83**	**86**
no malignant tumors with score >1	53	37	88	112
% relevantly positive tumors	**36**	**32**	**44**	**57**

*5. MS801: **Mesothelioma** tissue array with normal mesothelium*

This array included 80 cores from 40 patients. In addition to 30 mesothelioma tissues, 10 normal mesothelium tissues were included.

MS801: Mesothelioma tissue array with normal mesothelium

	TGF-ß1	TGF-ß2(Acris)	TGF-ß2(SC)	p-Smad2/3
mean/median (tumor)	1.47/1	1.37/1	1.70/2	1.87/2
mean/median (non-neoplastic)	2.54/3	0.94/1	0.92/1	0.77/1
standard deviation/IQR (tumor)	0.75/1	0.99/1	0.85/1	0.91/2
standard deviation/IQR (non-neoplastic)	0.97/0	0.64/0	0.28/0	0.60/1
f-test (tumor versus non-neoplastic)	0.1912	0.0478	0.0002	0.1126
t-test (tumor versus non-neoplastic)	**0.00565**	**0.03604**	**0.00246**	**0.00005**

	TGF-ß1	TGF-ß2(Acris)	TGF-ß2(SC)	p-Smad2/3
no. tumors	58	58	58	58
no. tumors with score 0	2	9	3	4
no. tumors with score 1	33	27	22	15
no. tumors with score 2	16	11	21	22
no. tumors with score 3	7	11	12	17
no. tumors with score >0	56	49	55	54
% positive tumors	**97**	**84**	**95**	**93**
no tumors with score >1	23	22	33	39
% relevantly positive tumors	**40**	**38**	**57**	**67**

6a. PA1921: Mid-advanced stage ***pancreatic cancer*** *tissue array*

This array contained 192 cores of 96 patients, the broad majority of them corresponding to pancreatic adenocarcinoma. Tumors on this array were rather homogeneous in terms of their malignant stage. 77 cases were middle stage (stage II) and only 19 cases in an advanced stage (stage III or IV).

PA1921: Mid-advanced stage pancreatic cancer tissue array

	TGF-ß1	TGF-ß2(Acris)	TGF-ß2(SC)	p-Smad2/3
mean/median (tumor)	1.19/1	1.22/1	1.27/1	1.53/2
standard deviation/IQR (tumor)	0.70/0	0.74/1	0.69/1	0.68/1

	TGF-ß1	TGF-ß2(Acris)	TGF-ß2(SC)	p-Smad2/3
no. tumors	166	188	192	192
no. tumors with score 0	18	27	17	9
no. tumors with score 1	108	101	115	84
no. tumors with score 2	30	52	51	88
no. tumors with score 3	10	8	9	11
no. tumors with score >0	148	161	175	183
% positive tumors	**89**	**86**	**91**	**95**
no tumors with score >1	40	60	60	99
% relevantly positive tumors	**24**	**32**	**31**	**52**

*6b. PA2081a: **Pancreatic disease** spectrum tissue array*

This array contained 208 cores of 103 patients. 52 cases of primary and metastic pancreatic carcinoma are spotted representing all malignant stages. Also pancreatic islet cell tumors, pancreatic inflammation and non-neoplastic pancreatic tissues are represented.

PA2081a: Pancreatic disease spectrum tissue array

	TGF-ß1	TGF-ß2(Acris)	TGF-ß2(SC)	p-Smad2/3
mean/median (adenocarinoma)	1.02/1	1.02/1	1.13/1	1.56/2
mean/median (non-neoplastic)	1.85/2	1.16/1	1.10/1	1.23/1
mean/median (inflammation)	2.05/2	1.14/1	1.59/2	1.64/2
standard deviation/IQR (adenocarcinoma)	0.72/0	0.71/0	0.74/1	0.88/1
standard deviation/IQR (non-neoplastic)	0.92/2	0.49/0	0.43/0	0.61/1
standard deviation/IQR (inflammation)	0.79/1.75	0.77/1	0.50/1	0.66/1
f-test (adenocarcinoma vs. non-neopl.)	0.03143	0.00157	0.00001	0.00251
t-test (adenocarcinoma vs. non-neopl.)	**1.68982E-08**	0.17	0.71	0.010
f-test (adenocarcinoma vs. inflammation)	0.57	0.55	0.05	0.13
t-test (adenocarcinoma vs. inflammation)	**0.000004**	0.52	**0.001**	0.64
f-test (inflammation vs. non-neopl.)	0.424	0.005	0.359	0.641
t-test (inflammation vs. non-neopl.)	0.36	0.89	**0.0003**	**0.01**

	TGF-ß1	TGF-ß2(Acris)	TGF-ß2(SC)	p-Smad2/3
no. tumors (adenocarcinomas only)	104	104	104	104
no. tumors with score 0	22	22	18	12
no. tumors with score 1	62	61	58	37
no. tumors with score 2	14	16	18	35
no. tumors with score 3	4	3	4	15
no. tumors with score >0	80	80	80	87
% positive tumors	**77**	**77**	**77**	**84**
no tumors with score >1	18	19	22	50
% relevantly positive tumors	**17**	**18**	**21**	**48**

*7. BC03119: **Liver** carcinoma and normal tissue*

This array included 120 cores from 120 patients, in their majority hepatocellular carcinoma and in addition 10 non-neoplastic tissues.

BC03119: Liver carcinoma and normal tissue

	TGF-ß1	TGF-ß2(Acris)	TGF-ß2(SC)	p-Smad2/3
mean/median (tumor)	1.59/1	0.71/1	1.55/2	1.65/2
mean/median (non-neoplastic)	1.80/2	1.10/1	2.20/2	0.80/1
standard deviation/IQR (tumor)	0.80/1	0.66/1	0.96/1	0.81/1
standard deviation/IQR (non-neoplastic tissue)	0.42/0	0.74/0	0.63/0.75	0.63/0.75
f-test (tumor versus non-neoplastic)	0.04	0.53	0.17	0.42
t-test (tumor versus non-neoplastic)	0.186	0.137	**0.011**	**0.002**
no. tumors	104	100	108	108
no. tumors with score 0	5	39	16	10
no. tumors with score 1	48	52	37	31
no. tumors with score 2	36	8	35	54
no. tumors with score 3	15	1	20	13
no. tumors with score >0	99	61	92	98
% positive tumors	**95**	**61**	**85**	**91**
no tumors with score >1	51	9	55	67
% relevantly positive tumors	**49**	**9**	**51**	**62**

*8. CO1503: **Colon Cancer** tissue array*

This array contained 150 cores from 75 patients. 61 patients presented with an adenocarcinoma, a few rarer subentities were also represented.

CO1503: Colon Cancer tissue array

	TGF-ß1	TGF-ß2(Acris)	TGF-ß2(SC)	p-Smad2/3
mean/median (malignant tumor)	1.26/1	0.84/1	1.25/1	1.44/1
standard deviation/IQR (malignant tumor)	0.70/1	0.64/1	0.64/1	0.83/1

	TGF-ß1	TGF-ß2(Acris)	TGF-ß2(SC)	p-Smad2/3
no. malignant tumors	94	89	138	140
no. malignant tumors with score 0	10	26	10	17
no. malignant tumors with score 1	54	51	89	59
no. malignant tumors with score 2	26	12	34	50
no. malignant tumors with score 3	4	0	5	14
no. malignant tumors with score >0	84	63	128	123
% positive malignant tumors	**89**	**71**	**93**	**88**
no malignant tumors with score >1	30	12	39	64
% relevantly positive malignat tumors	**32**	**13**	**28**	**46**

*9. HN483: **Multiple head and neck cancer** with normal tissue array*

This array contained 48 cores of 48 patients, 40 squamous cell carcinomas and 8 non-neoplastic tissue samples.

HN483: Multiple head and neck cancer with normal tissue array

	TGF-ß1	TGF-ß2(Acris)	TGF-ß2(SC)	p-Smad2/3
mean/median (tumor)	1.08/1	0.76/1	1.43/1.5	1.59/2
mean/median (non-neoplastic)	0.33/0	0.57/1	1.50/1.5	1.13/1
standard deviation/IQR (tumor)	0.49/0	0.68/1	0.78/1	0.59/1
standard deviation/IQR (non-neoplastic)	0.58/0.5	0.53/1	0.53/1	0.35/0
f-test (tumor versus non-neoplastic)	0.52	0.56	0.30	0.15
t-test (tumor versus non-neoplastic)	0.148	0.441	0.745	**0.009**

	TGF-ß1	TGF-ß2(Acris)	TGF-ß2(SC)	p-Smad2/3
no. tumors	38	37	40	39
no. tumors with score 0	3	14	5	0
no. tumors with score 1	29	18	15	18
no. tumors with score 2	6	5	18	19
no. tumors with score 3	0	0	2	2
no. tumors with score >0	35	23	35	39
% positive tumors	**92**	**62**	**88**	**100**
no tumors with score >1	6	5	20	21
% relevantly positive tumors	**16**	**14**	**50**	**54**

*10. ME2082b: **Malignant Melanoma** tissue array*

This array contained 128 cases of primary malignant melanoma, 64 metastatic malignant melanoms and 8 cases each of adjacent normal skin tissue and normal skin tissue.

ME2082b: Malignant Melanoma tissue array

	TGF-ß1	TGF-ß2(Acris)	TGF-ß2 (SC)	p-Smad2/3
mean/median (all)	0.71/0	0.44/0	1,38/1	1.32/1
mean/median (primary tumors only)	0.73/0	0.46/0	1.39/1	1.45/1
mean/median (metastases only)	0.69/0	0.42/0	1.38/1	1.06/1
standard deviation/IQR (all)	0.89/1	0.69/1	0.87/1	1.01/2
standard deviation/IQR (primary tumors only)	0.89/1	0.70/1	0.87/1	1.05/1
standard deviation/IQR (metastases only)	0.91/1	0.69/1	0.89/1	0.88/2

**primary versus metastatic tumors**	TGF-ß1	TGF-ß2(Acris)	TGF-ß2 (SC)	p-Smad2/3
f-test	0.87	0.85	0.81	0.12
t-test	0.77	0.75	0.97	**0.01**

	TGF-ß1	TGF-ß2(Acris)	TGF-ß2 (SC)	p-Smad2/3
no. primary tumors	125	125	122	121
no. primary tumors with score 0	66	82	19	28
no. primary tumors with score 1	32	30	49	34
no. primary tumors with score 2	22	12	42	36
no. primary tumors with score 3	5	1	12	23
no. primary tumors with score >0	59	43	103	93
% positive primary tumors	**47**	**34**	**84**	**77**
no. primary tumors with score >1	27	13	54	59
% relevantly positive primary tumors	**22**	**10**	**44**	**49**
no. metastatic tumors	64	64	63	63
no. metastatic tumors with score 0	36	43	10	20
no. metastatic tumors with score 1	15	16	26	21
no. metastatic tumors with score 2	10	4	20	20
no. metastatic tumors with score 3	3	1	7	2
no. metastatic tumors with score >0	28	21	53	43
% positive metastatic tumors	**44**	**33**	**84**	**68**
no. metastatic tumors with score >1	13	5	27	22
% relevantly positive metastatic tumors	**20**	**8**	**43**	**35**

*11a. GL803a: **Brain tumor** and adjacent tissue array*

This array contained 80 cores from 80 patients. Diagnoses represented were astrocytomas of all malignancy grades and in lower numbers oligodendrogliomas, ependymomas and medulloblastomas.

GL803a: Brain tumor and adjacent tissue array

	TGF-ß1	TGF-ß2(Acris)	TGF-ß2(SC)	p-Smad2/3
mean/median (tumor)	1.53/1	0.64/1	1.27/1	1.27/1.5
mean/median (NAT)	1.20/1	0.40/0	1.80/2	1.20/1
standard deviation/IQR (tumor)	0.79/1	0.61/1	0.68/1	0.80/1
standard deviation/IQR (NAT)	0.45/0	0.55/1	0.45/0	0.45/0

	TGF-ß1	TGF-ß2(Acris)	TGF-ß2(SC)	p-Smad2/3
no. tumors	70	73	74	74
no. tumors with score 0	3	31	6	7
no. tumors with score 1	37	37	46	30
no. tumors with score 2	20	5	18	30
no. tumors with score 3	10	0	4	7
no. tumors with score >0	67	42	68	67
% positive tumors	**96**	**58**	**92**	**91**
no tumors with score >1	30	5	22	37
% relevantly positive tumors	**43**	**7**	**30**	**50**

*11b. GL2083a: **Brain tumor** tissue array*

This array contained 208 cores from 208 patients. Astrocytomas of all grades were represented as well as a few oligodendrogliomas and ependymomas, 1 ganglioglioma and a gliosarcoma.

GL2083a: Brain tumor tissue array

	TGF-ß1	TGF-ß2(Acris)	TGF-ß2(sc)	p-Smad2/3
mean/median (malignant)	0.80/1	0.40/0	1.15/1	1.44/1
mean/median (NAT)	1.19/1	0.31/0	0.94/1	1.44/1
standard deviation/IQR (malignant)	0.57/1	0.54/1	0.84/1	0.74/1
standard deviation/IQR (NAT)	0.40/0	0.48/1	0.57/0	0.51/1

	TGF-ß1	TGF-ß2(Acris)	TGF-ß2(sc)	p-Smad2/3
no. malignant tumors	192	192	191	192
no. malignant tumors with score 0	53	120	43	17
no. malignant tumors with score 1	127	67	89	86
no. malignant tumors with score 2	10	5	47	77
no. malignant tumors with score 3	2	0	12	12
no. malignant tumors with score >0	139	72	148	175
% positive malignant tumors	**72**	**38**	**77**	**91**
no tumors with score >1	12	5	59	89
% relevantly positive tumors	**6**	**3**	**31**	**46**

*12a. LY2086: **Lymphoma** tumor tissue array*

This array contained 208 cores from 208 patients. Tumor entities comprised in their large majority T-cell lymphoma and in fewer number Hodgkin's lymphoma.

LY2086: Lymphoma tumor tissue array

	TGF-ß1	TGF-ß2(Acris)	TGF-ß2(SC)	p-Smad2/3
mean/median (malignant)	1.56/1	0.73/1	1.40/1	1.65/2
mean/median (NAT)	1.64/2	1.00/1	1.69/2	0.69/0.5
standard deviation/IQR (malignant)	0.81/1	0.48/1	0.80/1	0.89/1
standard deviation/IQR (NAT)	1.03/1	0.53/0	0.95/1	0.79/1

	TGF-ß1	TGF-ß2(Acris)	TGF-ß2(SC)	p-Smad2/3
no. malignant tumors	185	176	180	186
no. malignant tumors with score 0	6	50	19	17
no. malignant tumors with score 1	101	123	87	66
no. malignant tumors with score 2	47	3	57	68
no. malignant tumors with score 3	31	0	17	35
no. malignant tumors with score >0	179	126	161	169
% positive malignant tumors	**97**	**72**	**89**	**91**
no malignant tumors with score >1	78	3	74	103
% relevantly positive tumors	**42**	**2**	**41**	**55**

*12b. LM803: **Lymphoma** and normal lymph node tissue array*

This array comprised 89 cores from 80 patients. In contrast to the lymphoma array LY2086 a far higher number of Hodgkin's lymphoma were included.

LM803: Lymphoma and normal lymph node tissue array

	TGF-ß1	TGF-ß2(Acris)	TGF-ß2(SC)	p-Smad2/3
mean/median (tumor)	1.08/1	0.88/1	1.12/1	1.33/1
mean/median (NAT+normal)	1.75/2	0.75/1	1.05/1	0.80/1
standard deviation/IQR (tumor)	0.49/0	0.58/0	1.06/1.25	0.66/1
standard deviation/IQR (NAT+normal)	0.85/1	0.45/0.25	0.39/0	0.77/1
f-test (tumor versus NAT+normal)	0.00182	0.24989	0.00002	0.35276
t-test (tumor versus NAT+normal)	**0.0001**	0.3309	0.7846	**0.0093**

	TGF-ß1	TGF-ß2(Acris)	TGF-ß2(SC)	p-Smad2/3
no. tumors	50	60	60	60
no. tumors with score 0	4	14	19	4
no. tumors with score 1	38	39	26	34
no. tumors with score 2	8	7	4	20
no. tumors with score 3	0	0	11	2
no. tumors with score >0	46	46	41	56
% positive tumors	**92**	**77**	**68**	**93**
no tumors with score >1	8	7	15	22
% relevantly positive tumors	**16**	**12**	**25**	**37**

*13. BM483: Tumor tissue array (**Myeloma**)*

This array contained 10 cases of myeloma, 1 each of primitive neuroectodermal tumor (PNET), ewing's sarcoma and B-cell lymphoma as well as 11 adjacent normal tissues.

BM483: Tumor tissue array (Myeloma)

	TGF-ß1	TGF-ß2(Acris)	TGF-ß2(SC)	p-Smad2/3
mean/median (malignant)	1.04/1	0.77/1	2.00/2	2.04/2
mean/median (NAT)	0.57/1	0.05/0	1.95/2	2.05/2
standard deviation/IQR (malignant)	0.96/1	0.71/1	0.83/0.25	0.77/1.75
standard deviation/IQR (NAT)	0.60/1	0.22/0	0.62/0	0.59/0

	TGF-ß1	TGF-ß2(Acris)	TGF-ß2(SC)	p-Smad2/3
no. malignant tumors	26	26	24	26
no. malignant tumors with score 0	8	10	2	0
no. malignant tumors with score 1	12	12	2	7
no. malignant tumors with score 2	3	4	14	11
no. malignant tumors with score 3	3	0	6	8
no. malignant tumors with score >0	18	16	22	26
% positive malignant tumors	**69**	**62**	**92**	**100**
no tumors with score >1	6	4	20	19
% relevantly positive tumors	**23**	**15**	**83**	**73**

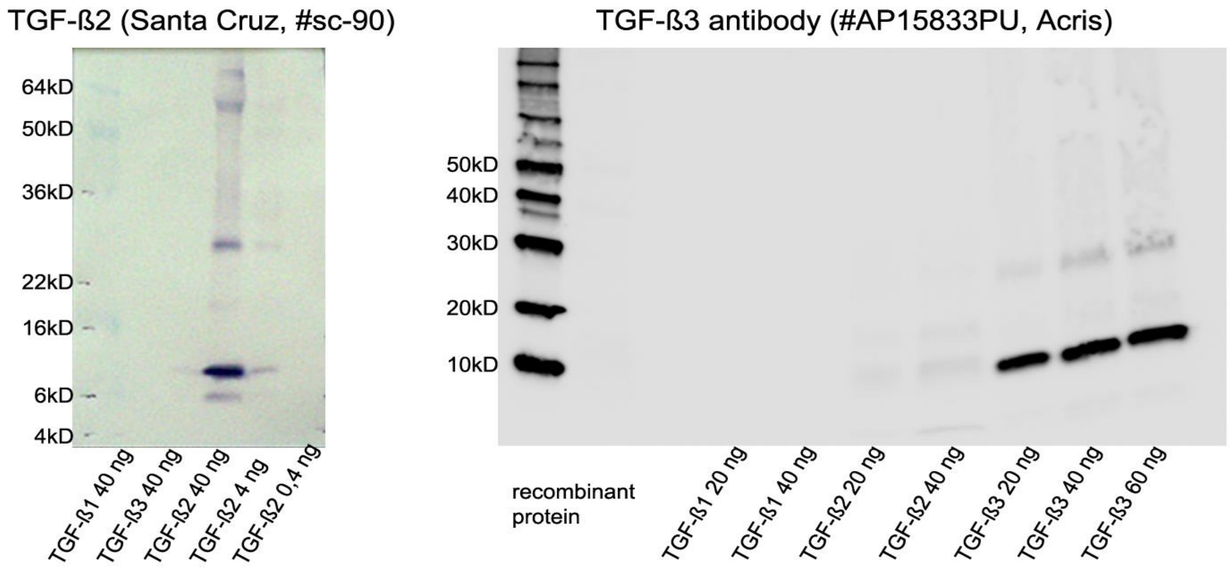

Suppl. Fig. 1. Western Blot with the TGF-ß2 (#sc-90, Santa Cruz) and TGF-ß3 (#AP15833PU; Acris) antibodies. The antibodies specifically detect their mature recombinant proteins (13 kDA), while other TGF-ß isoform proteins are not detected.

a) TGF-ß1 (Acris, #DM1047)

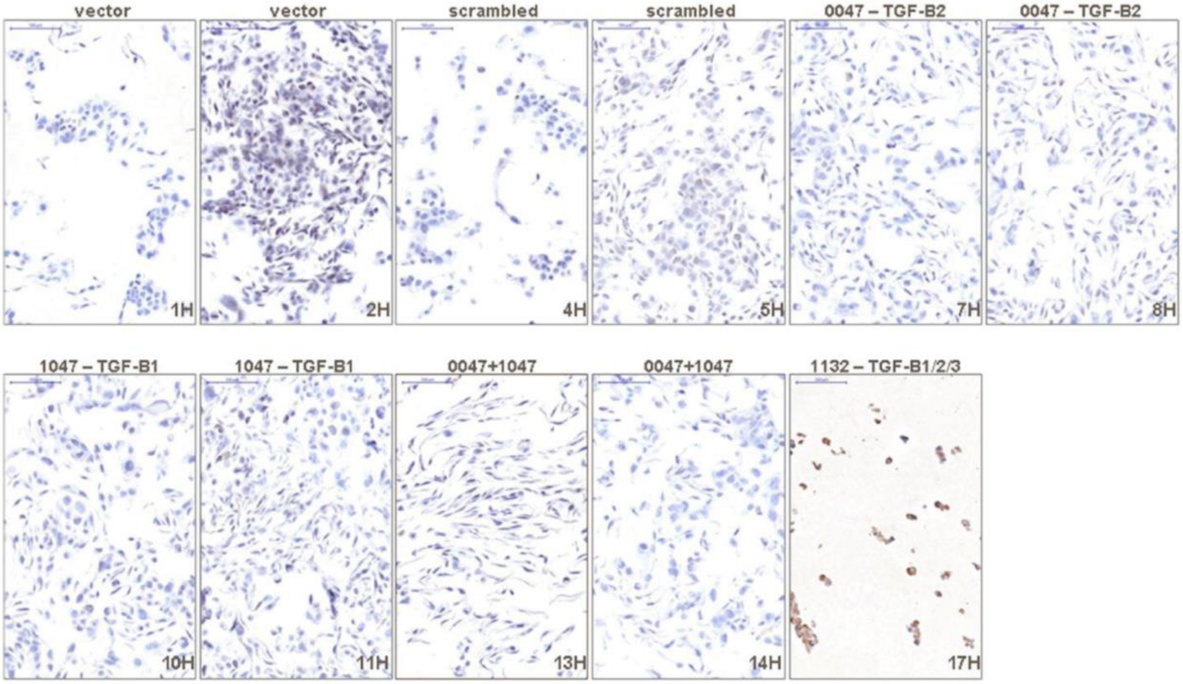

b) TGF-ß2 (Acris, #AP15815PU-S)

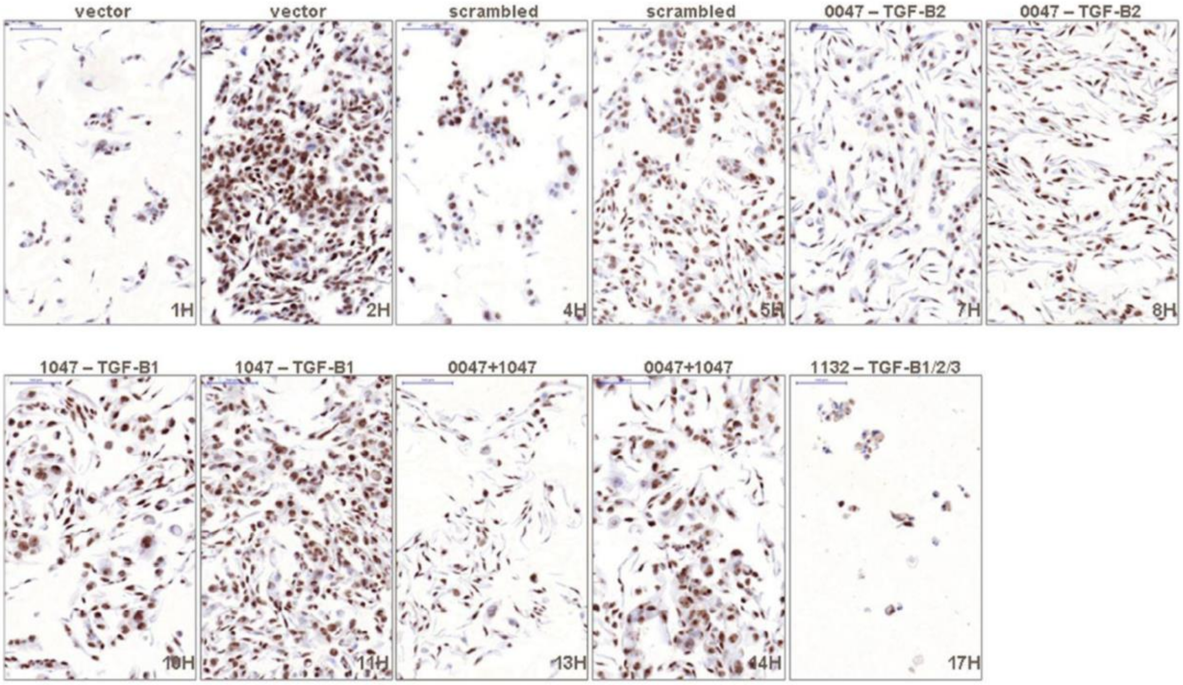

c) TGF-ß2 (Santa Cruz, #sc-90)

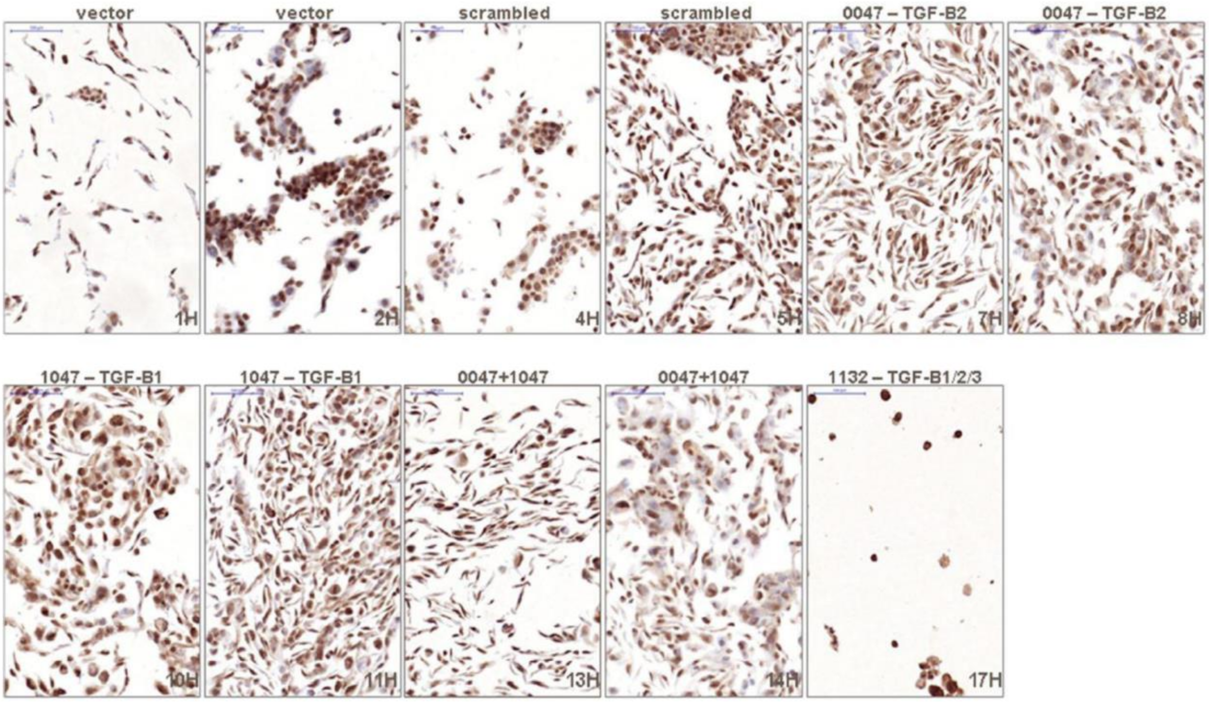

d) p-smad2/3 (Cell Signaling, #3101)

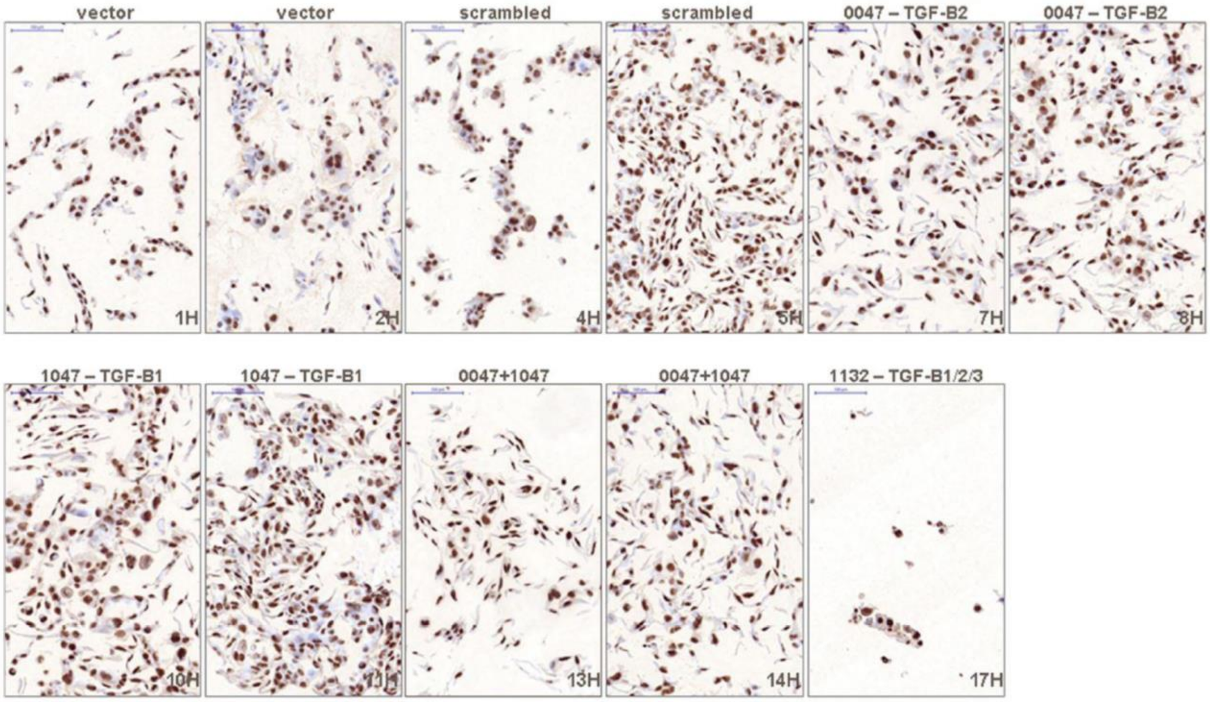

Suppl. Fig. 2. Examples of the immunostaining of the Panc-1 cells after TGF-ß directed antisense oligonucleotide treatment. The samples are labeled according to the designations provided in Table 4b (05630001H-05630017H). None of the antibodies tested showed meaningful differences in staining intensity or number of immunostained cells comparing the different treatment conditions.

IHC rank		tumor type	mean RNA expression levels (normalized counts)			
			TGF-ß1 (M38449_s_at)	TGF-ß2 (M19154_at)	TGFß2 (Y00083_s_at)	summarized counts
15	1	melanoma (n=10)	518.8029816	92.22132165	46.51372303	657.5380262
7, 14	2	lymphoma (n=21)	445.7583659	47.88632752	61.82900614	555.4736996
12, 16	3	glioblastoma (n=10)	434.4905856	53.67501842	53.01794676	541.1835508
8,11	4	pancreatic cancer (n=10)	433.7509922	51.8387372	55.08222447	540.6719539
2	5	breast cancer (n=11)	416.5357252	50.99076521	54.58112188	522.1076123
1	6	mesothelioma (n=10)	414.3056125	51.17197145	56.13678955	521.6143735
5	7	lung cancer (n=10)	349.0043729	52.22546347	49.47681837	450.7066547
13	8	prostate cancer (n=9)	350.1304083	51.77626536	42.15465464	444.0613283
10	9	colorectal cancer (n=10)	345.5460034	43.8339605	49.97468894	439.3546528
4	10	ovarian cancer (n=10)	325.7428922	49.07791618	51.53101096	426.3518194

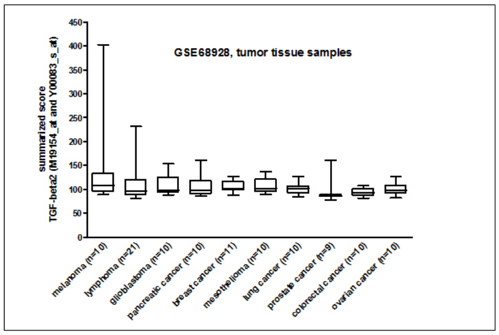

IHC rank		tumor type	mean RNA expression levels (normalized counts)		
			TGF-ß2 (M19154_at)	TGFß2 (Y00083_s_at)	summarized counts
15	1	melanoma (n=10)	92.22132165	46.51372303	138.7350447
7,14	2	lymphoma (n=21)	47.88632752	61.82900614	109.7153337
1	3	mesothelioma (n=10)	51.17197145	56.13678955	107.308761
8,11	4	pancreatic cancer (n=10)	51.8387372	55.08222447	106.9209617
12,16	5	glioblastoma (n=10)	53.67501842	53.01794676	106.6929652
2	6	breast cancer (n=11)	50.99076521	54.58112188	105.5718871
5	7	lung cancer (n=10)	52.22546347	49.47681837	101.7022818
4	8	ovarian cancer (n=10)	49.07791618	51.53101096	100.6089271
13	9	prostate cancer (n=9)	51.77626536	42.15465464	93.93092
10	10	colorectal cancer (n=10)	43.8339605	49.97468894	93.80864944

IHC rank		tumor type	mean RNA expression levels (normalized counts)
			TGF-ß1 (M38449_s_at)
15	1	melanoma (n=10)	518.8029816
7,14	2	lymphoma (n=21)	445.7583659
12,16	3	glioblastoma (n=10)	434.4905856
8,11	4	pancreatic cancer (n=10)	433.7509922
2	5	breast cancer (n=11)	416.5357252
1	6	mesothelioma (n=10)	414.3056125
13	7	prostate cancer (n=9)	350.1304083
5	8	lung cancer (n=10)	349.0043729
10	9	colorectal cancer (n=10)	345.5460034
4	10	ovarian cancer (n=10)	325.7428922

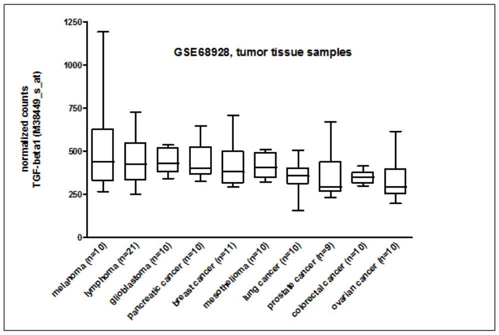

Suppl. Fig. 3. Ranking of tumor types according to TGF-ß ligand mRNA expression. Raw expression data from Gene Expression Omnibus (GEO) dataset GSE68928 were normalized using the R package affylmGUI (R version 3.2.0; affylmGUI Version 1.42.0; limma Version 3.24.14) and employing the RMA (Robust Multi-array Average) method. TGF-beta 1 was represented with one probe set (M38449_s_at) on the array (Affymetrix Human Full Length HuGeneFL Array), while TGF-beta 2 was represented with two probe sets (M19154_at and Y00083_s_at). Normalized signaling intensities for the different probe sets were analyzed in a comparable fashion to the immunohistochemistry stainings on the tissue arrays (summarized count for the TGF-ß1 and both TGF-ß2 probe sets). As there were huge differences in the normalized counts between the TGF-ß1 and the TGF-ß2 probe sets, tumor types were additionally ranked to either TGF-ß1 and or TGF-ß2 expression alone. The different mRNA expression based-ranks did not well overlap with the protein expression (immunohistochemistry)-based ranks of the respective tumor types.
